# Diverse patterns of antibody variable gene repertoire disruption in patients with amyloid light chain (AL) amyloidosis

**DOI:** 10.1371/journal.pone.0235713

**Published:** 2020-07-07

**Authors:** Elaine C. Chen, Samuel Rubinstein, Cinque Soto, Robin G. Bombardi, Samuel B. Day, Luke Myers, Alexey Zaytsev, Mahsa Majedi, R. Frank Cornell, James E. Crowe

**Affiliations:** 1 Department of Pathology Microbiology, and Immunology, Vanderbilt University Medical Center, Nashville, TN, United States of America; 2 The Vanderbilt Vaccine Center, Vanderbilt University Medical Center, Nashville, TN, United States of America; 3 Division of Hematology and Oncology, Department of Medicine, Vanderbilt University Medical Center, Nashville, TN, United States of America; 4 Department of Pediatrics, Vanderbilt University Medical Center, Nashville, TN, United States of America; Chang Gung University, TAIWAN

## Abstract

Immunoglobulin light chain amyloidosis is the most common form of systemic amyloidosis. AL amyloidosis is caused by a misfolded light chain produced by a clonal population of plasma cells. Disease status currently is defined by measuring the absolute quantity of serum free light chain protein, but this measurement often fails to identify the subclinical presence of clonal cells that may merit additional therapy. Next generation sequencing has the sensitivity to measure the relative amount of dominating light chains within the repertoire of a patient, and this technique is in clinical use to identify clonal populations of plasma cells for multiple myeloma, a related disorder. In this proof-of-concept study, we used bone marrow aspirates of AL amyloidosis positive patients and used reverse transcription of the antibody transcriptome followed by next generation sequencing to identify antibody variable-diversity-joining gene sequences for patients with immunoglobulin light chain amyloidosis, and demonstrate that this technology can be used to identify the dominant clone. The data also reveal differing patterns of overall antibody repertoire disruption in different patients. This method merits further study in larger prospective studies to establish its utility in detecting residual disease for patients with immunoglobulin light chain amyloidosis.

## Introduction

Amyloidoses are systemic illnesses caused by the extracellular deposition into tissue of amyloid proteins, which are generally subunits of normal serum proteins consisting largely of beta-pleated sheet regions. The most common amyloidosis in the United States is light chain (AL) amyloidosis, in which the amyloidogenic protein typically is free antibody light chain secreted by a population of plasma cells generally thought to be clonal [1]. The current best practices for determining patient hematologic disease status involve measuring the absolute quantity of free light chain proteins in serum [2, 3]. Free light chain ratio is determined by measuring serum free light chains in patients and identifying the kappa-to-lambda light chain ratio. A complete hematologic remission is defined in part by normalization of the free light chain ratio. However, many patients with complete hematologic responses do not experience an organ response, or they even experience organ progression [3–5]. Furthermore, some patients present with relatively low levels of serum free light chain protein and discordantly advanced organ involvement [6]. One plausible mechanism for this finding is the persistence of small numbers of cells from plasma cell clones that continue to produce amyloidogenic light chain protein, which contributes to progressive organ dysfunction at low concentrations. As many as 60% of AL amyloidosis patients in complete hematologic response may have residual clonal amyloidogenic plasma cell populations, as measured by next generation flow cytometric analysis of circulating white blood cells [7, 8]. More sensitive and specific methods for determining hematologic disease status are needed.

Early in B cell development, immunoglobulin germline genes rearrange to encode the B cell receptor expressed on naïve B cells, which then is modified and diversified by somatic hypermutation after B cells respond to antigen in germinal centers. This process allows B cells and plasma cells in a lineage derived from a single cell (a clone) to persist with one distinct heavy chain and one light chain sequence. Emerging high-throughput sequencing (HTS) technologies have made it possible to sequence the heavy and light chain variable regions of millions or billions of B and T cell receptors from single samples, making it possible to identify and track clonal lymphocyte populations. The ability to track clonal populations of B or T cells has implications for monitoring disease progression over time in settings where one might track the proliferation of an aberrant B cell population involved in cancer progression. The clonoSEQ assay by Adaptive Biotechnologies [9] was approved by the FDA for the detection and monitoring of minimal residual disease (MRD) in bone marrow samples from multiple myeloma or B-cell acute lymphoblastic leukemia (ALL) patients [9]. The Adaptive Biotechnologies assay uses genomic DNA as input and has been shown to provide sufficient sensitivity for clinical utility [9]. While the clonoSEQ assay is the only FDA-approved assay to date using immune repertoire sequencing as a clinical diagnostic test, the technology is relatively expensive and does not provide full-length sequencing of the variable region, which is of interest for following B cell clones that can expand in lineages due to somatic hypermutation. We tested the performance of an alternative reverse transcription-based methodology that uses mRNA-based sequencing (mRNA) to determine if we could identify the dominant clonal populations and features of immune repertoire disruption for patients with AL amyloidosis. The mRNA-based method provides full-length sequencing of the antibody variable region, which is pertinent to following the occurrence of somatic mutations outside of the junctional region targeted in the more size-limited amplicons in current genomic DNA-based methods. We identified the dominant clonotype in each subject studied, we also tracked the clonal population of the dysplastic B cell in one subject over time. With this approach, we identified a subset of patients with dysplastic B-cells that also expressed high levels of a particular heavy chain protein. Interestingly, when we examined the top ten most frequently represented mRNA clonotypes present in the antibody gene transcripts for each subject, we identified somatic variants of several clones, suggesting that AL amyloidosis disease might be driven in part by antigen stimulation. This proof-of-concept study demonstrates the applicability of mRNA-based sequencing for the detection of over-represented clonal populations and important features of the overall immune repertoire in B cells from patients with AL amyloidosis. This will help identify the clonal plasma cells in patients with hematologically active disease.

## Methods

Bone marrow samples were obtained from patients through the Vanderbilt University Medical Center (VUMC) hematologic malignancy tissue bank after written informed consent was obtained. The study was approved by the Vanderbilt University Medical Center Institutional Review Board. Mononuclear cells were isolated from the bone marrow aspirate samples from seven subjects (designated AM1, AM2, AM3, AM4, AM5, AM6, or AM7) with AL amyloidosis with or without multiple myeloma; samples collected at three separate timepoints for subject AM2 were included. Approximately 2 × 10^6^ mononuclear cells per donor sample that had been cryopreserved previously were thawed, washed and counted with a viability dye. The estimated number of B cells in the sample was determined by multiplying the total viable mononuclear cell count in the sample by the percent of plasma cells in each subject. The cells were pelleted in a tabletop centrifuge prior to isolation of total cell RNA from the resulting pellet using the RNeasy Mini Kit (Qiagen), according to the manufacturer’s recommendations. Each resulting pool of purified RNA was split equally by volume to create aliquots for up to three technical replicates for subsequent target enrichment using a previously described 5′RACE enrichment approach. [10] Briefly, a cDNA synthesis primer mix (10 μM each) was combined with template RNA and incubated at 70°C for 2 min, and then the incubation temperature was decreased to 42°C to anneal the synthesis primers (5 min). Primer annealed template RNA then was mixed with 5X First-strand buffer (Clontech), DTT (20 mM from the SMARTScribe synthesis kit; Clontech), 5′ template switch oligo (TSO) adapter containing a Unique Molecular Identifier (10 μM), dNTP mix (10 mM each), 20 units RNAse inhibitor (RNAsin, Promega) and 10X SMARTscribe Reverse Transcriptase (Clontech) prior to incubation at 42°C for 60 min. Immediately following reverse transcription, first-strand cDNA was purified using AMPure SPRIselect beads (Beckman Coulter) at a ratio of 1X per volume RT reaction. Purified cDNA was PCR amplified by mixing with 2X Q5 Master Mix (NEB), dNTP solution (10 mM each), universal forward primer extending off the TSO (10 μM), gene-specific first PCR reverse primer mix (10 μM). PCR 1 cycling was performed as follows: 95°C for 1 min 30s followed by 18 cycles of 95°C for 10s, 60°C for 20s and 72°C for 40s, followed by a final extension of 72°C for 4 min. First-round PCR products were purified using AMPure SPRIselect beads at a ratio of 0.6X per volume PCR 1 reaction (Beckman Coulter). PCR 2 cycling was performed identically to PCR 1 except using extension primers with sample indexes and performing 14 cycles for PCR 2. Final PCR products were purified using AMPure SPRIselect beads at a ratio of 0.6X per volume RT reaction (Beckman Coulter). Concentrations were determined by fluorometric quantitation (Qubit) and pooled by combining equimolar portions of each individual sample. Pooled PCR products were used to generate a sequencing library using the NEBNext Ultra Library Prep Kit for Illumina sequencing by applying standard protocol according to the manufacturer’s recommendations. Libraries were sequenced on an Illumina MiSeq platform using MiSeq Reagent Kit V3 (600 cycles) and symmetric PE-300 sequencing. All primers used in this protocol were modified from a previously described approach [10] and are listed (S2 Table).

The bioinformatics processing of all NGS data was performed using the PyIR sequence processing pipeline that uses IgBLAST [11]. Briefly, we merged all paired-end (PE) reads to generate full-length contigs using the program USEARCH v9.18 [12]. The overlap region (-*fastq_minovlen*) was set to 15 nucleotides, and the maximum number of differences in the overlap region (*-fastq_maxdiffpct*) was set to 10. All merged reads were filtered in the following order using our MongoDB database: (1) Removal of any read that had an average Phred score of less than 30; (2) Removal of any read that had an E-value larger than 10^−6^ for *IGHV*/*IGHJ* germline assignments; (3) Removal of any read that did not have a defined CDR3; (4) Removal of any read containing a stop codon; (5) Removal of any read that was out of frame at the junction region; and (6) Removal of reads for which the nucleotide length from framework 1 through framework 3 was less than 250 nucleotides. All remaining reads were labeled as productive reads (but not error-corrected). To correct for any sequencing errors, we binned all raw reads associated with a productive read, using a Universal Molecular Identifier (UMI), CDR3 length (in amino acids) and variable (V) and joining (J) gene assignment (ignoring allele). The grouped raw reads were re-oriented from 5′ to 3′ and then used to generate a consensus sequence based on a the most frequent nucleotide at each position. The consensus reads from each grouping were then processed again using our PyIR pipeline and subjected to the same set of filters already described above. All remaining error-corrected reads were labeled productive and used for all analysis. Authors E.C.C. and C.S. analyzed the data, and all authors had access to primary data.

### Data sharing statement

The dataset(s) used in this article are available in the Sequence Read Archive (SRA, https://www.ncbi.nlm.nih.gov/sra) under Bioproject number PRJNA637633.

## Results

Four patients had detailed clinical information available for review. The remaining patients consented for tissue bank participation but not linkage with the electronic health record. The four patients with detailed clinical information had active hematologic disease at the time of sample collection. Three had newly diagnosed, untreated AL amyloidosis, and the fourth had relapsed after initial therapy. Organ involvement was proven by biopsy in three patients (AM1, AM2, AM3) and was clinically suspected in a fourth (AM5) on the basis of proven fat pad involvement and a suggestive cardiac presentation and echocardiogram. Organ involvement was as follows: 1) cardiac, renal, and duodenal involvement, 2) cardiac and renal involvement, 3) duodenal involvement alone, and 4) cardiac involvement alone. All patients had active AL amyloidosis disease at the time the samples were acquired (Table 1).

**Table 1:** Results of antibody gene repertoire sequence analysis experiments for bone marrow aspirate specimens from seven patients with AL amyloidosis.

Patient	Clinical tissue status [cardiac (C), renal (R), duodenal (D) or not available (na)]	Number of viable plasma cells* in the sample (x 10^5^)	Light chain variable gene sequences obtained after de-duplication of biological replicates**		Genetic features of the dominant light chain variable gene***		
			Total unique reads	Total unique clonotypes	V genes	J genes	CDR3 (amino acids)
AM1	C, R, D	1.2	3,921	1,683	*IGLV3-21*	*IGLJ3*	QVWDRSSDRPV
AM2 *Time-point 1*	na	5.1	8,601	4,937	***IGLV3-25***	***IGLJ2***	**QSADSSGTYEVI**
AM2 *Time-poin 2*	na	0.7	2,331	1,165	***IGLV3-25***	***IGLJ2***	**QSADSSGTYEVI**
AM2 *Time-point 3*	na	6.8	1,702	630	***IGLV3-25***	***IGLJ2***	**QSADSSGTYEVI**
AM3	C	4.0	4,524	2,885	*IGLV1-47*	*IGLJ1*	AAWDGSLSGYV
AM4	na	0.4	1,788	1,307	***IGLV2-14***	***IGLJ1***	**SS**F**T**SSSSY**V**
AM5	C, R	1.0	577	266	***IGLV2-14***	***IGLJ1***	**SS**Y**T**ITNTL**V**
AM6	na	1.1	1,316	919	*IGKV3-20*	*IGKJ4*	QQYGTSPLT
AM7	na	1.5	475	223	*IGLV6-57*	*IGLJ3*	QSYQGSSGV

* The total number of viable mononuclear cells in the aliquot of cryopreserved bone marrow aspirate sample was multiplied by plasma cell percentage to achieve number of viable plasma cells in the sample.

** Three replicates for: AM1, AM2 Time-point 1, AM2 Time-point 2, AM3, and AM4. Two replicates for: AM2 Time-point 3. One replicate for:AM5, AM6, and AM7.

*** **Bold** or **bold/underlined** entries highlight two different sets of samples with common genetic features.

The medical records of the patients showed that clinical testing revealed the patient bone marrow aspirate mononuclear cell suspensions had a median of 14% plasma cells (range: 6 to 90), and the serum had a median free light chain concentration of 4.11 mg/dL (range, 2.7 to 118) at the time points studied. Three patients had lambda light chain disease, and one had kappa light chain disease; in all cases, immunofixation was free light chain only. Three had Mayo stage III disease, and one had Mayo stage II disease.

The viability of the previously cryopreserved mononuclear cells in the bone marrow aspirate suspension after thawing ranged from 30 to 42%. The estimated number of B cells per donor used in the sequencing reactions ranged between 1.7 × 10^4^ to 2.3 × 10^4^ B cells (Table 1). We sequenced the heavy and light chain immune repertoires of seven donors using Illumina’s paired-end (PE) Miseq platform (see Methods for details). We then used the PyIR bioinformatics pipeline [13] to process and correct errors in the reads. After processing and error correction, we obtained an average of 1,963 unique and productive heavy chain variable gene reads per donor and an average of 2,803 unique and productive light chain variable gene reads per donor (Table 1). We adopted the V3J clonotype definition from Soto *et al*. [14] to group together somatic variants belonging to the same lineage. A V3J clonotype was defined as comprising clones with sequences using the identical CDR3 amino acid sequence and the same V and J germline gene assignments (ignoring allelic distinctions). Thus, any somatic variants sharing these three properties were considered to belong to the same V3J clonotype and thus the same B cell clonal lineage. After grouping somatic variants using the V3J clonotype definition, we obtained an average of 1,393 unique V3J clonotypes per donor for heavy chains and an average of 1,557 unique V3J clonotypes per donor for light chains (Table 1). For downstream analysis of the dominant V3J clonotype, we pooled together all replicate samples from the same subject.

One of the hallmarks of AL amyloidosis is the presence of a single clone that dominates the light chain repertoires. We sought to ask if there is diversity in the light chain variable genes that were over-represented in the next generation sequencing (NGS) of these subjects. One metric for measuring diversity often used in immune repertoire sequencing is Shannon entropy [14]. We computed the Shannon entropy value for the light chain repertoires using the V3J clonotype definition and found values for the light chain repertoire for each subject: AM1 at 1.66, AM2 at 5.02, 1.14, 0.10, AM3 at 4.5, AM4 at 6.03, AM5 at 0.34, AM6 at 1.19, and AM7 at 0.51. For comparison, we analyzed the Shannon entropy in three healthy subjects (designated HIP1, 2, or 3) using large repertoire data sets from Soto *et al*., [14]. The Shannon entropy values for each of the healthy subjects were as follows: HIP1 at 9.80, HIP2 at 9.47, and HIP3 at 8.76. Thus, the repertoire of amyloidogenic patients clearly exhibited a general profound lack of diversity, consistent with the presence of a dominant clonotype.

**Fig 1:**
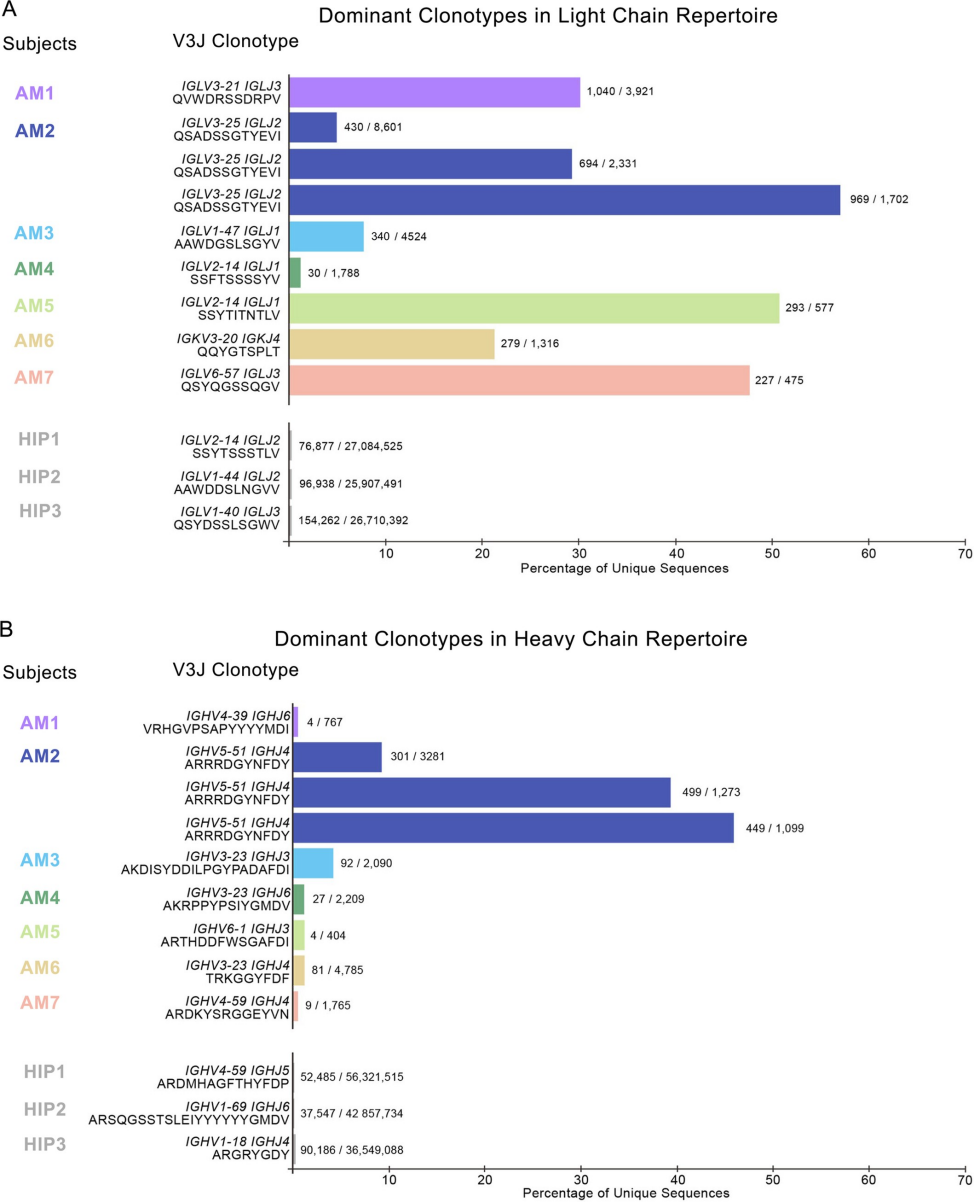
Dominant clonotypes in the light and heavy chain immune repertoires of subjects afflicted with light chain amyloidosis. (A) Most abundant light chain V3J clonotypes in each subject (B) Most abundant heavy V3J clonotypes in each subject. Percentages were obtained by dividing the total number of unique sequences containing the V3J clonotype in each subject by the total number of unique sequences for the entire repertoire of each subject. The somatic variant count for the most prevalent V3J clonotype appears at the end of each bar graph. For comparison, we also included sequencing data from 3 healthy subjects denoted as HIP1, HIP2 or HIP3 [14].

To determine the fraction of heavy and light chain repertoires accounted for by each V3J clonotype, we divided the total number of unique somatic variants associated with each clonotype by the total number of unique somatic variants for that subject (Fig 1). Since AL amyloidosis is a disease affecting the immunoglobulin light chain, we reasoned that the V3J clonotype with the largest number of somatic variants likely corresponded to the variable gene of the aberrant light chain sequence causing the disease. For three of the seven subjects considered here (AM2, AM5 and AM7) a single V3J clonotype accounted for approximately 50% of the light chain repertoire (Fig 1A). If we relaxed the cutoff to just 30% of the total number of unique reads, four of seven repertoires tested (AM1, AM2, AM5 and AM7) had a single dominant V3J clonotype for the light chain repertoire. A comparison with light chain sequencing from three healthy subjects revealed that in those healthy subjects the most prevalent single V3J clonotype accounted for less than 1% of the light chain repertoire (Fig 1A, see subjects HIP1, HIP2 or HIP3). While none of the subjects with AL amyloidosis shared the exact same dominant V3J clonotype in their light chain repertoire, two out of the seven subjects shared the identical V-J gene combination and the identical CDR3 length for their dominant V3J clonotype (Table 1, Fig 1A and S1 Table). The dominant V3J clonotype from the heavy chain sequencing from these seven subjects was much less pronounced (Fig 1B). In fact, four out of seven subjects (AM1, AM5, AM6, AM7) had a similar profile for clonotype dominance as seen in heavy chain sequencing from three healthy donors HIP1, HIP2 or HIP3. However, we identified three subjects with what appeared to be a corresponding heavy chain that was highly over-expressed (Fig 1, subjects AM2, AM3, and AM4). This finding suggests that the dysplastic cell clone in these patients coordinately over-expressed both an antibody light chain and a heavy chain. In contrast, the repertoires of the other subjects studied showed over-expression of an antibody light chain only.

We examined the reproducibility of the findings in the cases where we generated two or three technical replicates on the same RNA extract from a single aspirate sample. In each of the cases (separate replicates in total for light and heavy chains), the technical replicates provided identical results within the replicate group (S1 Table).

In one subject, we tracked the single aberrant V3J clonotype over three years to simulate the use of this technique in a clinical monitoring scenario, and found the clone was present with varying levels over time (see subject AM2 data in Table 1, Fig 1A and S1 Table). The samples were collected with 18 months between the first and second time point, and 4 months between the second and third time point. These data suggest that this method could be used for tracking the persistence of a dominant pathogenic clone in a patient over time.

We constructed a multiple sequence alignment using full length sequences from the dominant V3J clonotype within each donor, and for the three timepoints belonging to subject AM2. The V and J germline gene assignments of these light chain sequences were inferred from IgBlast [11], and aligned with the sequences from each donor. Within each donor, there are consistent somatic mutations throughout the length of the sequence persisting within most of the somatic variants of the dominant V3J clone. In AM1, these mutations include P8S, R19T, T21A in FR1, K30T in CDR1, K38T, V44L in FR2, F61L in FR3, and S92R, H96R in CDR3. In AM2, throughout all three time points, these mutations include: A28D, K31T and Q32K in CDR1, Y50H IN FR2, K51R and S53T in CDR2, P60S, V72A and Y89H in FR3, V100E, an valine insertion at 101, and V102I in the CDR3. There does not seem to be an ongoing hypermutation process during the period of three years when the samples were obtained from AM2. In AM3, these mutations include S25A in FR1, S31I in CR1, Q39H, L40F, T43A, K46N, and Y50H in FR2, G65A and S73F in FR3, D94G in CDR3, and T108I in FR4. In AM4, these mutations include S27G and G31T in CDR2, L48V in FR2, E52A in CDR2, Y93F and T98S in CDR3, and T103P in FR4. In AM5, these mutations include Y32D in CDR1, Y34H and G43D in FR2, S55T in CDR2, G60W, S62P, N63D, G78D, A90T, and Y97F in FR3, S103I, S104T, S105N, Y108L in CDR3, and T102S, K105R in FR4. In AM6, these mutations include R18G, T20N in CDR1, S28T, V29I and S32N in CDR1, A35T and P41F in FR2, A52Sin CDR2, S64T, S87N, V86M in FR3, and S94T in CDR3. In AM7, these mutations include T19I in FR1, S31T in CDR1, N52D in CDR2, S69R in FR3, D95Q, S96G, N98S, and W100G in CDR3. Additionally, every subject’s dominant clone CDR3 was mutated by at least one amino acid residue. (S1– S9 Figs)

**Fig 2:**
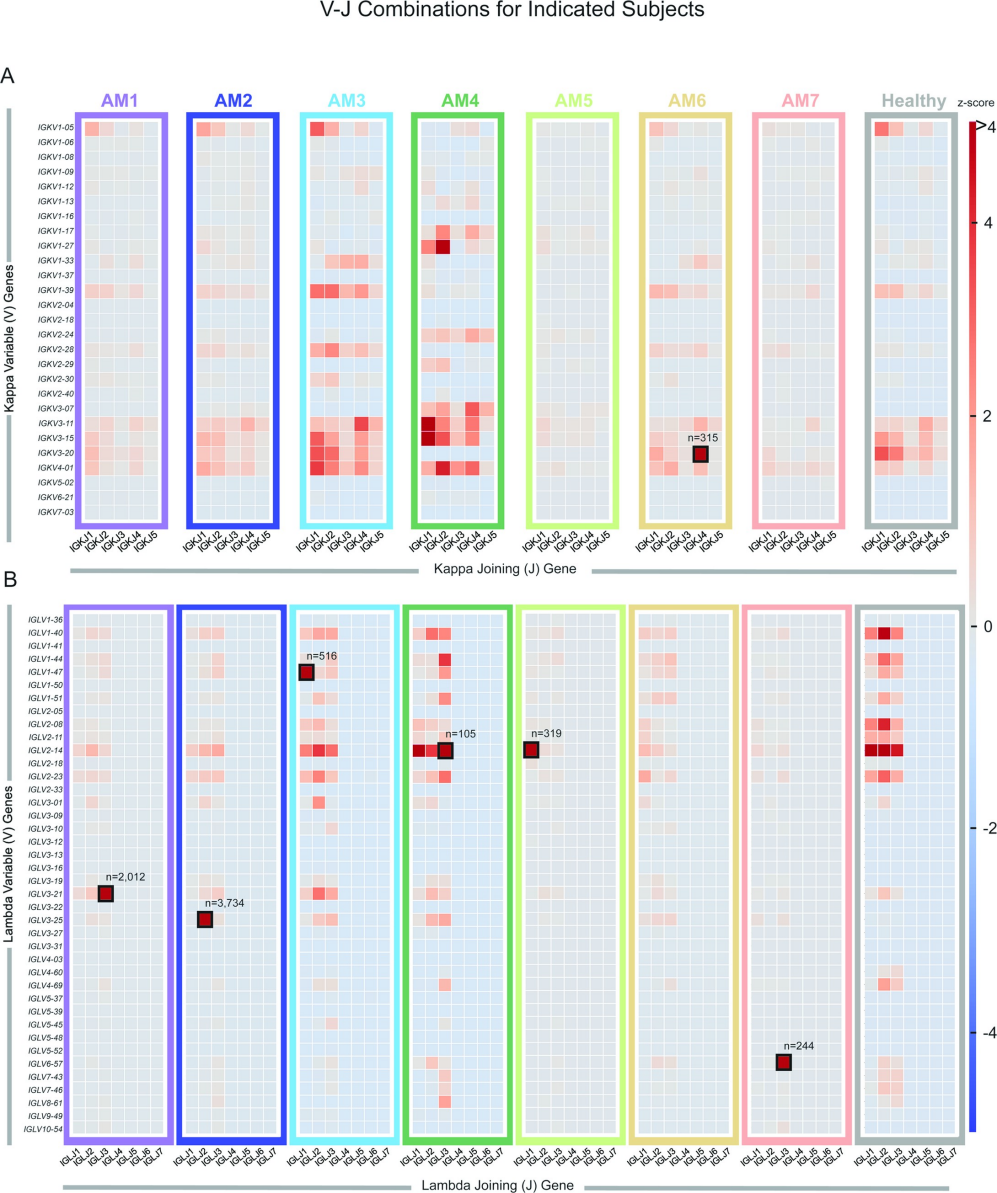
Light chain inferred V and J germline gene usage from repertoires belonging to subjects with amyloidosis. (A) Heatmap based on Vκ/Jκ germline gene usage. (B) Heatmap based on Vλ/Jλ germline gene usage. The frequency counts were derived from the total number of unique V3J clonotypes from each repertoire. The V/J frequency counts were transformed into a Z-score by first subtracting away the average frequency and then normalizing by the standard deviation of each subject. The colored box around each individual heatmap denotes repertoire data from each individual donor. The number of unique somatic variants for each dominant clonotype is indicated by a black box. For comparison we also included sequencing data from 3 healthy subjects from the Human Immunome Project (designated HIP1, HIP2 or HIP3). These data sets were combined and appear as the “healthy” dataset on the plot.

**Fig 3:**
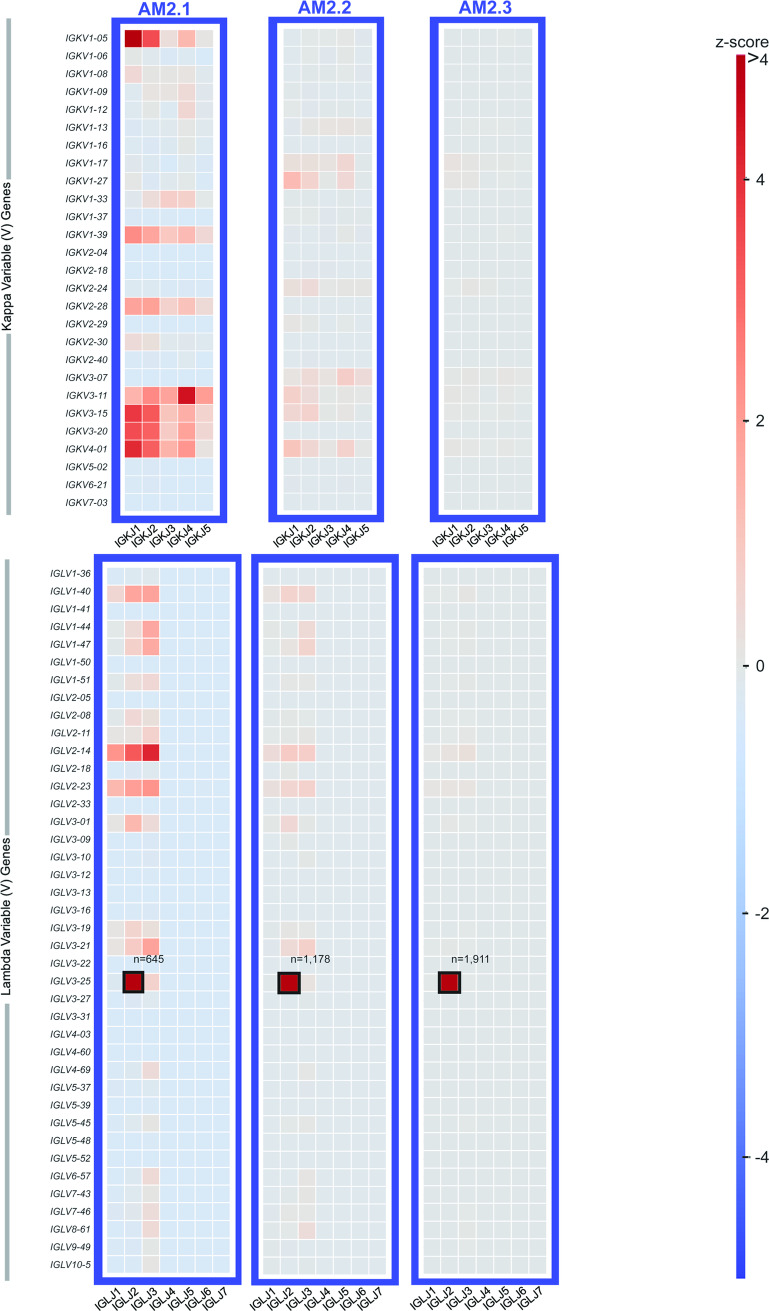
Light chain inferred V and J germline gene usage in the repertoire of subject AM2 over time. Heatmaps were generated from each of three time points at which a biopsy was taken. The heatmaps are based on Vκ/Jκ germline gene usage (upper panel) or Vλ/Jλ germline gene usage (lower panel). The V/J frequency counts were transformed into a Z-score by first subtracting away the average frequency and then normalizing by the standard deviation of each subject. The colored box around each individual heatmap denotes repertoire data from each individual donor. The number of unique somatic variants for each dominant clonotype is indicated by a black box.

**Fig 4:**
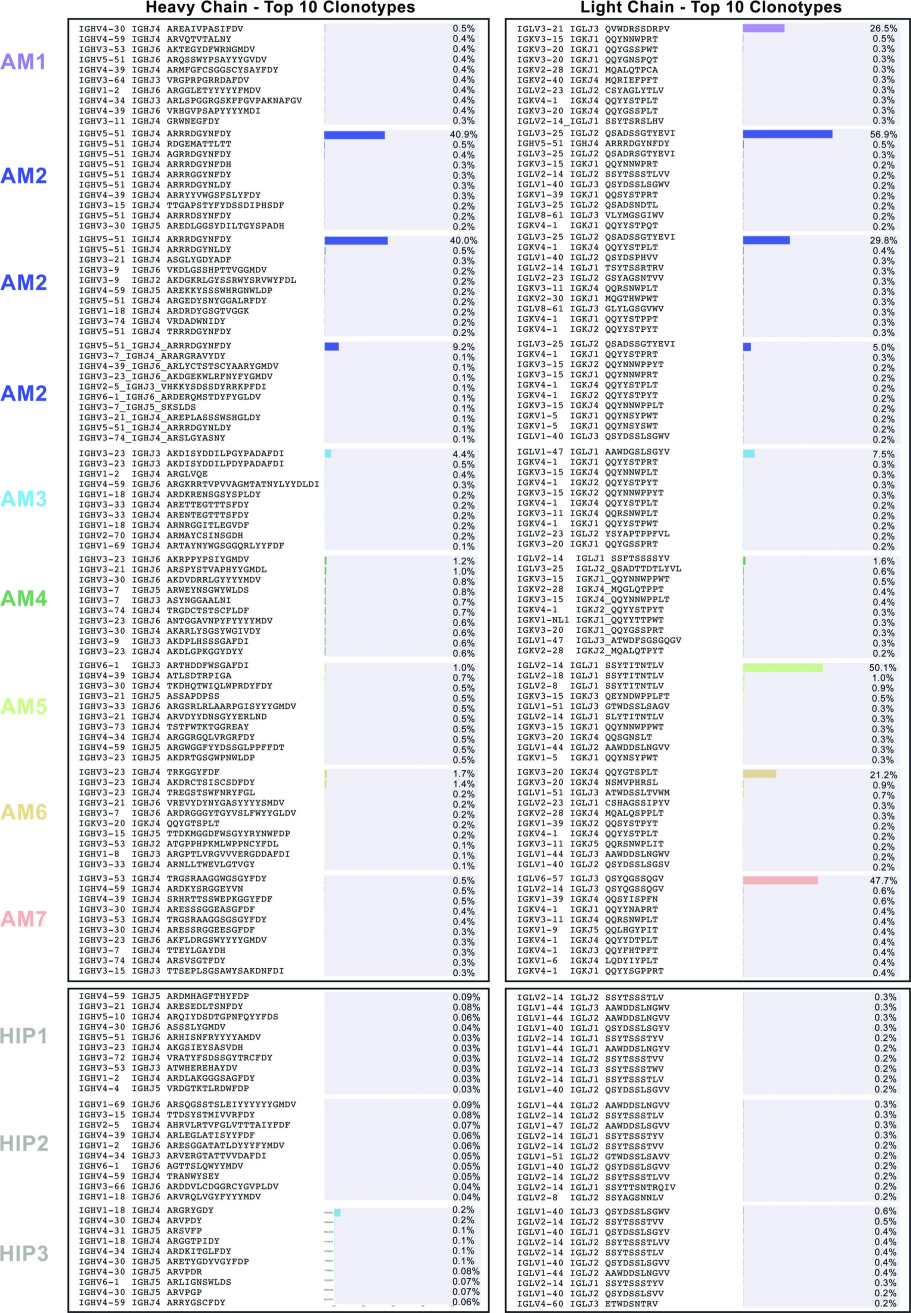
The ten most abundant V3J clonotypes ordered by the number of unique somatic variants. (A) Light chain V3J clonotypes (B) Heavy chain V3J clonotypes. Percentages were obtained by dividing the total number of somatic variants containing the V3J clonotype with the total number of somatic variants for the entire repertoire for each donor. The somatic variant count for the most prevalent V3J clonotype in each donor is shown at the top of each graph.

Heat maps were constructed showing the different V-J gene combinations used in the kappa (Fig 2) or lambda repertoire (Fig 2) for each subject. The number of sequences observed with the same V-J gene combinations are depicted in each heat map as a Z-score, normalized for each subject. The dominant clone described previously (Table 1 and Fig 1), could be recognized easily on the heat maps in Fig 2 (highlighted in the figure by black boxes), with the number of sequences containing the V-J combination denoted above each box. Also, we tracked the V-J combination containing the dominant clone in subject AM2 over the different time points in which biopsies were drawn (designated AM2.1, AM2.2 or AM2.3 in Fig 3A and 3B). The pathogenic clone was easily distinguished at all time points. Every sample contains its respective highly used V and J genes, including the healthy sample. However, despite the healthy control sample having some more highly used IGLV/IGLJ combinations, the healthy control samples do not have any light chain expanded clonotypes (V-J-CDR3). The highest representation of a clonotype within the healthy repertoires occurs at 0.6% of the repertoire, present in subject HIP3 (Fig 4). Additionally, out of all the amyloid patient samples, the dominant clone with the lowest representation is AM4, at about 1.6%, three-fold higher than what was seen in subject HIP3 (Fig 4). This finding shows that, although there are some light chain V-J combinations that are more highly used than others, there is a need to go a level deeper, and examine the V-J-CDR3 clonotype to determine the dysplastic clone present in each patient. The abundance of the top clonotypes within each donor reveals the genotype of the dysplastic clones.

## Discussion

Together, these data show that the dominant V3J clonotype in AL amyloidosis patients can be identified using relatively shallow mRNA-based NGS repertoire sequencing, and the mRNA for the dominant clone can be tracked easily in patients over time. Since we used both heavy and light chain antibody gene sequencing, we also could distinguish between patients who exhibited only a dominant light chain mRNA and those with coordinated over-expression of a single heavy and light chain mRNA. It will be of interest in the future to determine if these diverse genetic features correlate with differences in clinical outcomes or response to therapy.

However, despite these encouraging results, this study does have several limitations. The sample size is relatively small, limiting our ability to draw sweeping conclusions. Many patients had a relatively high burden of hematologic disease for a population of patients with AL amyloidosis. All samples used in this study were obtained at time points during which the patients had active disease. Therefore, additional studies will be needed to determine the sensitivity and specificity of this method for patients with a lower burden of disease. By acquiring bone marrow aspirates of patients ranging from high to low burden of disease, sensitivity can be determined by doing an end-point dilution until the assay no longer detects the dysplastic clone after ranking the top ten clonotypes present in each patient. Specificity can be determined by comparing results from using the mRNA sequencing method discussed in this paper in parallel with the Adaptive Biotechnologies clonoSeq test. The clonoSeq test is currently FDA approved for monitoring measurable residual disease (MRD) in both B-cell acute lymphoblastic leukemia and multiple myeloma, and therefore would be a good way to measure specificity of the assay for detecting the dysplastic clones.

It would also be of interest to use this assay on samples from patients in apparent complete clinical remission to determine if this method can identify the subclinical persistence of residual clones in the bone marrow. Previously, we examined the expressed antibody variable gene repertoires from 10 different human tissues using RNA samples derived from a large number of individuals (see Briney *et al*., [15]). Mao *et al*., also looked at B-cell responses in a variety of tissues [15]. Based on our early work and the work of others, it should be possible to detect differences in clonal B-cell populations within different tissues from individuals with AL. Moreover, we should be able to detect differences in specific features of the repertoire between diseased and non-diseased tissues (*e*.*g*., CDR3 length or somatic mutation). Whether or not these differences are statistically significant when compared to sequencing from whole blood needs to be studied in more detail. Assuming we could obtain tissue samples from the organs of patients with and without AL, we see no limitation in applying our method to a much larger sample size. With potential access to a large bank of amyloidogenic tissues, this technology could be applied to investigate sequence features of light chains that are prominent for aggregating in certain tissues. Studies like this will be informative in patients that have amyloid deposits in less high-risk organs, and therefore subjecting them to close monitoring instead of toxic treatments. Despite these limitations of the current study, the data demonstrate the straightforward use of shallow mRNA-based antibody variable gene sequence analysis on small samples to identify the recombined genes distinguishing a clonal population of plasma cells for each of the patients under study. The data also offer intriguing snapshots of diversity in genetic features of the clones and repertoires in AL amyloidosis patients that were not previously appreciated. There are many methods being developed to measure minimal residual disease, especially for multiple myeloma. Both flow cytometric techniques and next generation sequencing techniques have proven to have great specificity. However, the main differentiator between the two methods is that the flow cytometric assay requires fresh samples and immediate processing after biopsy. Additionally, flow cytometric studies are often operator-dependent. Therefore, this study is valuable in establishing another assay option for assessing minimal disease, especially for AL amyloidosis. This proof-of-concept study suggests it will be of value to apply this method in a larger population of patients with lower burden of disease to define the role of somatic variation and coordinated heavy and light chain over-expression in pathogenesis and response to therapy.

## Supporting information

S1 Table Detailed results of antibody gene repertoire sequence analysis experiments for bone marrow aspirate specimens from seven patients with AL amyloidosis. Each subject’s number of sequence reads, unique clonotype, V and J gene of the dominant clone as well as CDR3 are listed in the table. Information is listed for both heavy and light chain repertoires. Technical replicates were analyzed to ensure the dominant clone remained the dominant clone in all replicate, indicated by the dashes.
(PDF)

S2 Table Primers used for next generation sequencing. Primers used for each step of library prep and sequencing are listed. All primers are a modified version that was provided by Chdakov at the time of sequencing to incorerate Illumina Nextera adapters instead of Truseq adapters [10].
(PDF)

S1 Fig Multiple sequence alignment of dominant V3J clone variants for subject AM1. Somatic variants of the dominant clone were aligned to inferred germline genes to create a multiple sequence alignment.
(PDF)

S2 Fig Multiple sequence alignment of dominant V3J clone variants for subject AM2 timepoint 1. Somatic variants of the dominant clone were aligned to inferred germline genes to create a multiple sequence alignment.
(PDF)

S3 Fig Multiple sequence alignment of dominant V3J clone variants for subject AM2 timepoint 2. Somatic variants of the dominant clone were aligned to inferred germline genes to create a multiple sequence alignment.
(PDF)

S4 Fig Multiple sequence alignment of dominant V3J clone variants for subject AM2 timepoint 3. Somatic variants of the dominant clone were aligned to inferred germline genes to create a multiple sequence alignment.
(PDF)

S5 Fig Multiple sequence alignment of dominant V3J clone variants for subject AM3. Somatic variants of the dominant clone were aligned to inferred germline genes to create a multiple sequence alignment.
(PDF)

S6 Fig Multiple sequence alignment of dominant V3J clone variants for subject AM4. Somatic variants of the dominant clone were aligned to inferred germline genes to create a multiple sequence alignment.
(PDF)

S7 Fig Multiple sequence alignment of dominant V3J clone variants for subject AM5. Somatic variants of the dominant clone were aligned to inferred germline genes to create a multiple sequence alignment.
(PDF)

S8 Fig Multiple sequence alignment of dominant V3J clone variants for subject AM6. Somatic variants of the dominant clone were aligned to inferred germline genes to create a multiple sequence alignment.
(PDF)

S9 Fig Multiple sequence alignment of dominant V3J clone variants for subject AM7. Somatic variants of the dominant clone were aligned to inferred germline genes to create a multiple sequence alignment.
(PDF)
