# Supplementary material for: Diverse patterns of antibody variable gene repertoire disruption in patients with amyloid light chain (AL) amyloidosis
Source: PLoS One. 2020 Jul 7;15(7):e0235713. doi: 10.1371/journal.pone.0235713 (PMC7340310; doi:10.1371/journal.pone.0235713)
Supplement: S1 Table — Each subject’s number of sequence reads, unique clonotype, V and J gene of the dominant clone as well as CDR3 are listed in the table. Information is listed for both heavy and light chain repertoires. Technical replicates were analyzed to ensure the dominant clone remained the dominant clone in all replicate, indicated by the dashes. (PDF) [file pone.0235713.s001.pdf]

Table S1. Detailed results of antibody gene repertoire sequence analysis experiments for bone marrow aspirate specimens from seven patients with AL amyloidosis

Patient	Light chain					Heavy chain				
	Sequences obtained		Genetic features of the dominant sequence			Sequences obtained		Genetic features of the dominant sequence		
	Sequence reads for each of 1-3 technical replicates	Unique clonotypes for each of 1-3 technical replicates	$V_{K\ or\ L}$ gene*	$J_{K\ or\ L}$ gene*	LCDR3 (amino acids)*	Sequence reads for each of 3 technical replicates	Unique clonotypes for each of 3 technical replicates	V_H gene*	J_H gene*	HCDR3 (amino acids)*
AM1	1,963<br>2,296<br>1,953	1,006<br>1,139<br>1,029	*IGLV3-21*<br>–<br>–	*IGLJ3*<br>–<br>–	QVWDRSSDRPV<br>-----------<br>-----------	351<br>391<br>357	340<br>366<br>334	*IGHV4-39*<br>–<br>–	*IGHJ6*<br>–<br>–	VRHGVPSAPYYYYMDI<br>----------------<br>----------------
AM2<br>*Time-point 1*	5,754<br>4,755	3,575<br>2,946	***IGLV3-25***<br>–	***IGLJ2***<br>–	**QSADSSGTYEVI**<br>------------	1,225<br>1,372<br>1,260	1,071<br>1,185<br>1,082	***IGHV5-51***<br>–<br>–	***IGHJ4***<br>–<br>–	**ARRRDGYNFDY**<br>-----------<br>-----------
Time-point 2	1,590<br>843<br>1,018	857<br>476<br>591	–<br>–<br>–	–<br>–<br>–	------------<br>------------<br>------------	470<br>601<br>640	288<br>322<br>344	–<br>–<br>–	–<br>–<br>–	-----------<br>-----------<br>-----------
Time-point 3	82<br>958<br>977	176<br>309<br>299	–<br>–<br>–	–<br>–<br>–	------------<br>------------<br>------------	353<br>456<br>497	185<br>241<br>240	–<br>–<br>–	–<br>–<br>–	-----------<br>-----------<br>-----------
AM3	2,020<br>2,457<br>2,250	1,489<br>1,727<br>1,647	*IGLV1-47*<br>–<br>–	*IGLJ1*<br>–<br>–	AAWDGSLSGYV<br>-----------<br>-----------	288<br>1,060<br>1,069	226<br>931<br>979	*IGHV3-23*<br>–<br>–	*IGHJ3*<br>–<br>–	AKDISYDDILPGYPADAFDI<br>--------------------<br>--------------------
AM4	994<br>788<br>989	817<br>659<br>807	*IGLV2-14*<br>–<br>–	*IGLJ1*<br>–<br>–	SSFTSSSSYV<br>----------<br>----------	614<br>837<br>819	269<br>300<br>291	*IGHV3-23*<br>–<br>–	*IGHJ6*<br>–<br>–	AKRPPYSIYGMDV<br>-------------<br>-------------
AM5	577	266	*IGLV2-14*	*IGLJ1*	SSYTITNTLV	154<br>191<br>113	151<br>187<br>110	*IGHV6-1*<br>–<br>–	*IGHJ3*<br>–<br>–	ARTHDDFWSGAFDI<br>--------------<br>--------------
AM6	1,316	919	*IGKV3-20*	*IGKJ4*	QQYGTSPLT	2,491<br>2,609<br>2,770	1,960<br>2,072<br>2,199	*IGHV3-23*<br>–<br>–	*IGHJ4*<br>–<br>–	TRKGGYFDF<br>---------<br>---------
AM7	475	223	*IGLV6-57*	*IGLJ3*	QSYQGSSGV	828<br>791<br>843	735<br>702<br>755	*IGHV4-59*<br>–<br>–	*IGHJ4*<br>–<br>–	ARDKYSRGGEYVN<br>-------------<br>-------------

* The – symbol indicates the genetic feature is identical to that of the sequence above.

Bolded features show consistency of the dominant clone within one individual over a time of 3 years.
