# Supplementary material for: Diverse patterns of antibody variable gene repertoire disruption in patients with amyloid light chain (AL) amyloidosis
Source: PLoS One. 2020 Jul 7;15(7):e0235713. doi: 10.1371/journal.pone.0235713 (PMC7340310; doi:10.1371/journal.pone.0235713)
Supplement: S2 Table — Primers used for each step of library prep and sequencing are listed. All primers are a modified version that was provided by Chdakov at the time of sequencing to incorerate Illumina Nextera adapters instead of Truseq adapters [10]. (PDF) [file pone.0235713.s002.pdf]

Table S2. Primers used for next generation sequencing		
**Primer**	**Application**	**Sequence**
First-strand cDNA synthesis		
SmartNNNext	5′ – template-switch oligo with sequencing illumina adapter. U = dU, rG-riboG	AGATGUGTAUAAGAGACAGNNNNUNNNNUNNNNUCTT(rG)$_4$
Human IGH cDNA synthesis primer mix		
hIGG_r1	Primer for cDNA synthesis, human IgG heavy-chain mRNA	GAAGTAGTCCTTGACCAGGCA
hIGM_r1	Primer for cDNA synthesis, human IgM heavy-chain mRNA	GTGATGGAGTCGGGAAGGAAG
hIGA_r1	Primer for cDNA synthesis, human IgA heavy-chain mRNA	GCGACGACCACGTTCCCATCT
hIGD_r1	Primer for cDNA synthesis, human IgD heavy-chain mRNA	GGACCACAGGGCTGTTATC
hIGE_r1	Primer for cDNA synthesis, human IgE heavy-chain mRNA	AGTCACGGAGGTGGCATTG
Human IGL cDNA synthesis primer mix		
hIGLC_r1	Primer for cDNA synthesis, human IgL light-chain mRNA	GCTCCCGGGTAGAAGT
hIGKC_r1	Primer for cDNA synthesis, human IgK light-chain mRNA	GCGTTATCCACCTTCC
First PCR amplification		
Common primer	Step-out primer, anneals on the switch adaptor	AGATGTGTATAAGAGACAG
Human IGH reverse primer mix		
Common-hIGGE_r2	Nested primer with sequencing illumina adaptor, human IgG/IgE heavy-chain cDNA	AGATGTGTATAAGAGACAGARGGGGAAGACSGATG
Common-hIGA_r2	Nested primer with sequencing illumina adaptor, human IgA heavy-chain cDNA	AGATGTGTATAAGAGACAGCAGCGGGAAGACCTTG
Common-hIGM_r2	Nested primer with sequencing illumina adaptor, human IgM heavy-chain cDNA	AGATGTGTATAAGAGACAGAGGGGGAAAAGGGTTG
Common-hIGD_r2	Nested primer with sequencing illumina adaptor, human IgD heavy-chain cDNA	AGATGTGTATAAGAGACAGATATGATGGGGAACAC
Human IGL reverse primer mix		
Common-hIGL_r2	Nested primer with sequencing illumina adaptor, human IgL heavy-chain cDNA	AGATGTGTATAAGAGACAGGYGGGAACAGAGTGAC
Common-hIGK_r2	Nested primer with sequencing illumina adaptor, human IgK heavy-chain cDNA	AGATGTGTATAAGAGACAGGATGGTGCAGCCACAG
Second PCR amplification		
F-common	Step-out primer with sequencing and P7 illumina adapters	TCGTCGGCAGCGTCAGATGTGTATAAGAGACAG
R- common	Step-out primer with sequencing and P5 illumina adapters	GTCTCGTGGGCTCGGAGATGTGTATAAGAGACAG
Third PCR amplification		
Fc_i7	Step-out primer with index 1 illumina adapter	CAAGCAGAAGACGGCATACGAGAT[i7]GTCTCGTGGGCTCGG
Fc_i5	Step-out primer with index 2 illumina adapter	AATGATACGGCGACCACCGAGATCTACAC[i5]TCGTCGGCAGCGTC
