# Supplementary material for: Diverse patterns of antibody variable gene repertoire disruption in patients with amyloid light chain (AL) amyloidosis
Source: PLoS One. 2020 Jul 7;15(7):e0235713. doi: 10.1371/journal.pone.0235713 (PMC7340310; doi:10.1371/journal.pone.0235713)

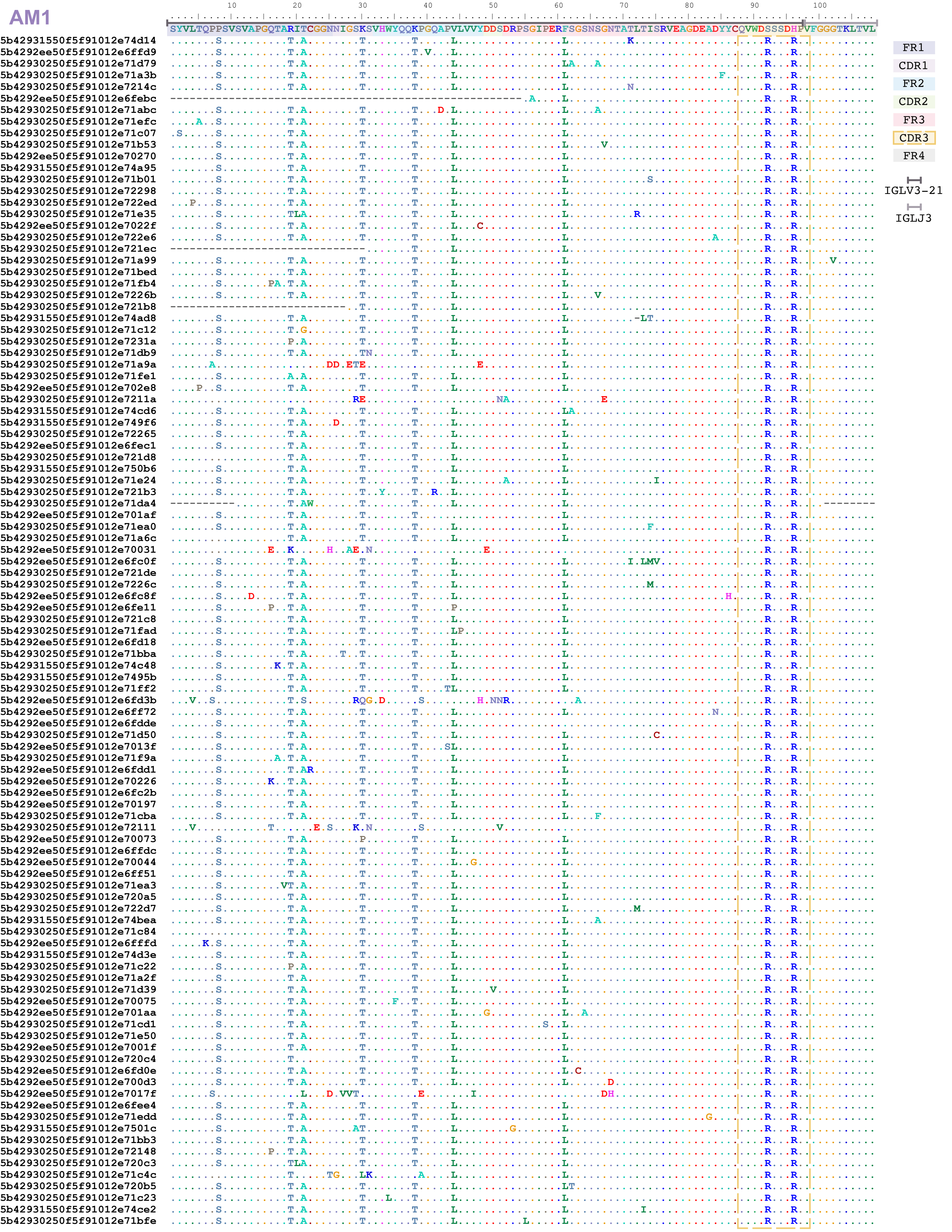

AM1
10 20 30 40 50 60 70 80 90 100
SYVLTQPPSVSVAPGQTARITCGGNNIGSKSVHWYQQKPGQAPVLVVYDDSDRPSGIPERFSGSNSGNTATLTISRVEAGDEADYYCQVWDSSSDHPVFGGGTKLTVL
FR1
CDR1
FR2
CDR2
FR3
CDR3
FR4
IGLV3-21
IGLJ3

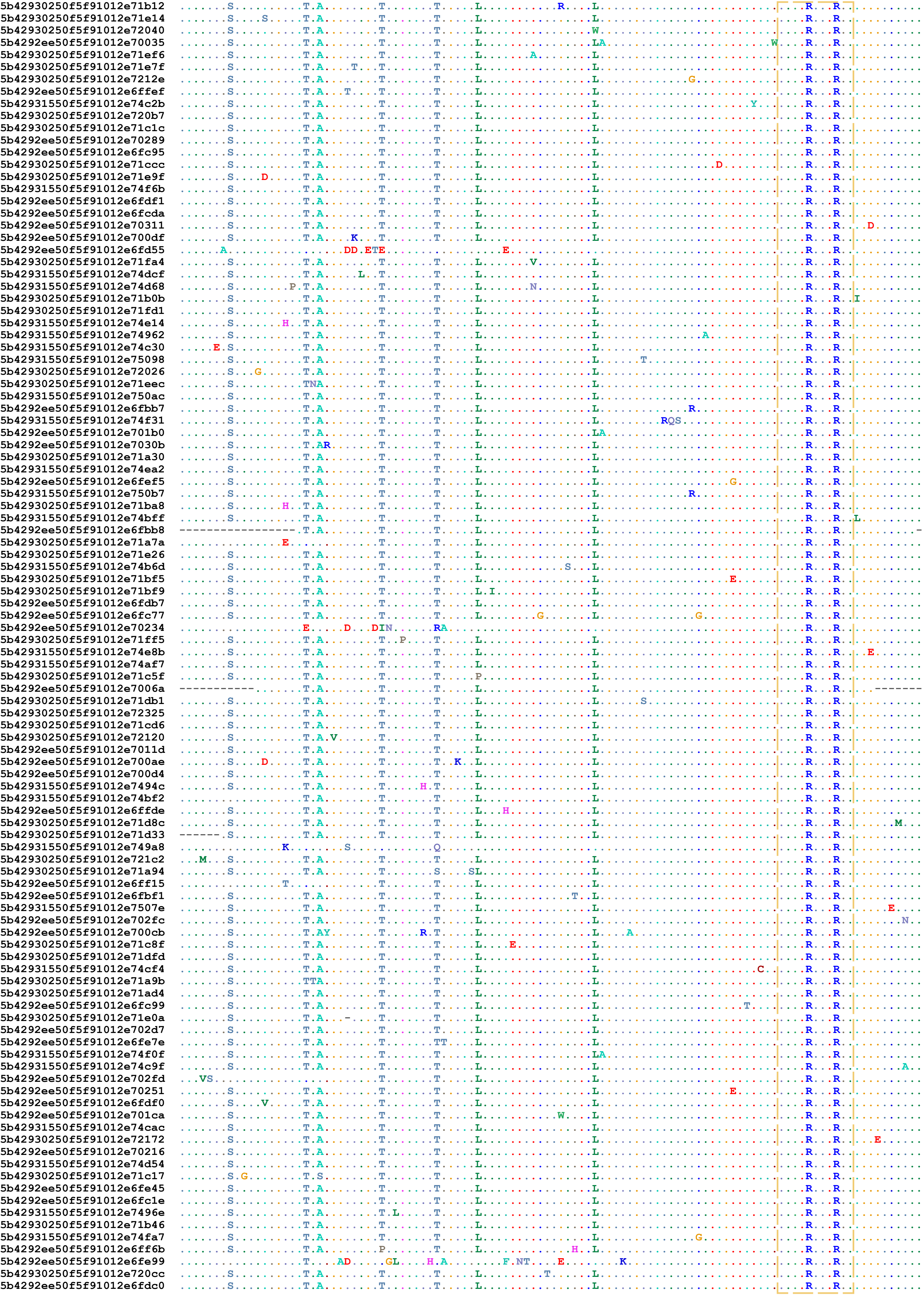
5b42930250f5f91012e71b12
5b42930250f5f91012e71e14
5b42930250f5f91012e72040
5b4292ee50f5f91012e70035
5b42930250f5f91012e71ef6
5b42930250f5f91012e71e7f
5b42930250f5f91012e7212e
5b4292ee50f5f91012e6ffef
5b42931550f5f91012e74c2b
5b42930250f5f91012e720b7
5b42930250f5f91012e71c1c
5b4292ee50f5f91012e70289
5b4292ee50f5f91012e6fc95
5b42930250f5f91012e71ccc
5b42930250f5f91012e71e9f
5b42931550f5f91012e74f6b
5b4292ee50f5f91012e6fdf1
5b4292ee50f5f91012e6fcda
5b4292ee50f5f91012e70311
5b4292ee50f5f91012e700df
5b4292ee50f5f91012e6fd55
5b42930250f5f91012e71fa4
5b42931550f5f91012e74dcf
5b42931550f5f91012e74d68
5b42930250f5f91012e71b0b
5b42930250f5f91012e71fd1
5b42931550f5f91012e74e14
5b42931550f5f91012e74962
5b42931550f5f91012e74c30
5b42931550f5f91012e75098
5b42930250f5f91012e72026
5b42930250f5f91012e71eec
5b42931550f5f91012e750ac
5b4292ee50f5f91012e6fbb7
5b42931550f5f91012e74f31
5b4292ee50f5f91012e701b0
5b4292ee50f5f91012e7030b
5b42930250f5f91012e71a30
5b42931550f5f91012e74ea2
5b4292ee50f5f91012e6fef5
5b42931550f5f91012e750b7
5b42930250f5f91012e71ba8
5b42931550f5f91012e74bff
5b4292ee50f5f91012e6fbb8
5b42930250f5f91012e71a7a
5b42930250f5f91012e71e26
5b42931550f5f91012e74b6d
5b42930250f5f91012e71bf5
5b42930250f5f91012e71bf9
5b4292ee50f5f91012e6fdb7
5b4292ee50f5f91012e6fc77
5b4292ee50f5f91012e70234
5b42930250f5f91012e71ff5
5b42931550f5f91012e74e8b
5b42931550f5f91012e74af7
5b42930250f5f91012e71c5f
5b4292ee50f5f91012e7006a
5b42930250f5f91012e71db1
5b42930250f5f91012e72325
5b42930250f5f91012e71cd6
5b42930250f5f91012e72120
5b4292ee50f5f91012e7011d
5b4292ee50f5f91012e700ae
5b4292ee50f5f91012e700d4
5b42931550f5f91012e7494c
5b42931550f5f91012e74bf2
5b4292ee50f5f91012e6ffde
5b42930250f5f91012e71d8c
5b42930250f5f91012e71d33
5b42931550f5f91012e749a8
5b42930250f5f91012e721c2
5b42930250f5f91012e71a94
5b4292ee50f5f91012e6ff15
5b4292ee50f5f91012e6fbf1
5b42931550f5f91012e7507e
5b4292ee50f5f91012e702fc
5b4292ee50f5f91012e700cb
5b42930250f5f91012e71c8f
5b42930250f5f91012e71dfd
5b42931550f5f91012e74cf4
5b42930250f5f91012e71a9b
5b42930250f5f91012e71ad4
5b4292ee50f5f91012e6fc99
5b42930250f5f91012e71e0a
5b4292ee50f5f91012e702d7
5b4292ee50f5f91012e6fe7e
5b42931550f5f91012e74f0f
5b42931550f5f91012e74c9f
5b4292ee50f5f91012e702fd
5b4292ee50f5f91012e70251
5b4292ee50f5f91012e6fdf0
5b4292ee50f5f91012e701ca
5b42931550f5f91012e74cac
5b42930250f5f91012e72172
5b4292ee50f5f91012e70216
5b42931550f5f91012e74d54
5b42930250f5f91012e71c17
5b4292ee50f5f91012e6fe45
5b4292ee50f5f91012e6fc1e
5b42931550f5f91012e7496e
5b42930250f5f91012e71b46
5b42931550f5f91012e74fa7
5b4292ee50f5f91012e6ff6b
5b4292ee50f5f91012e6fe99
5b42930250f5f91012e720cc
5b4292ee50f5f91012e6fdc0

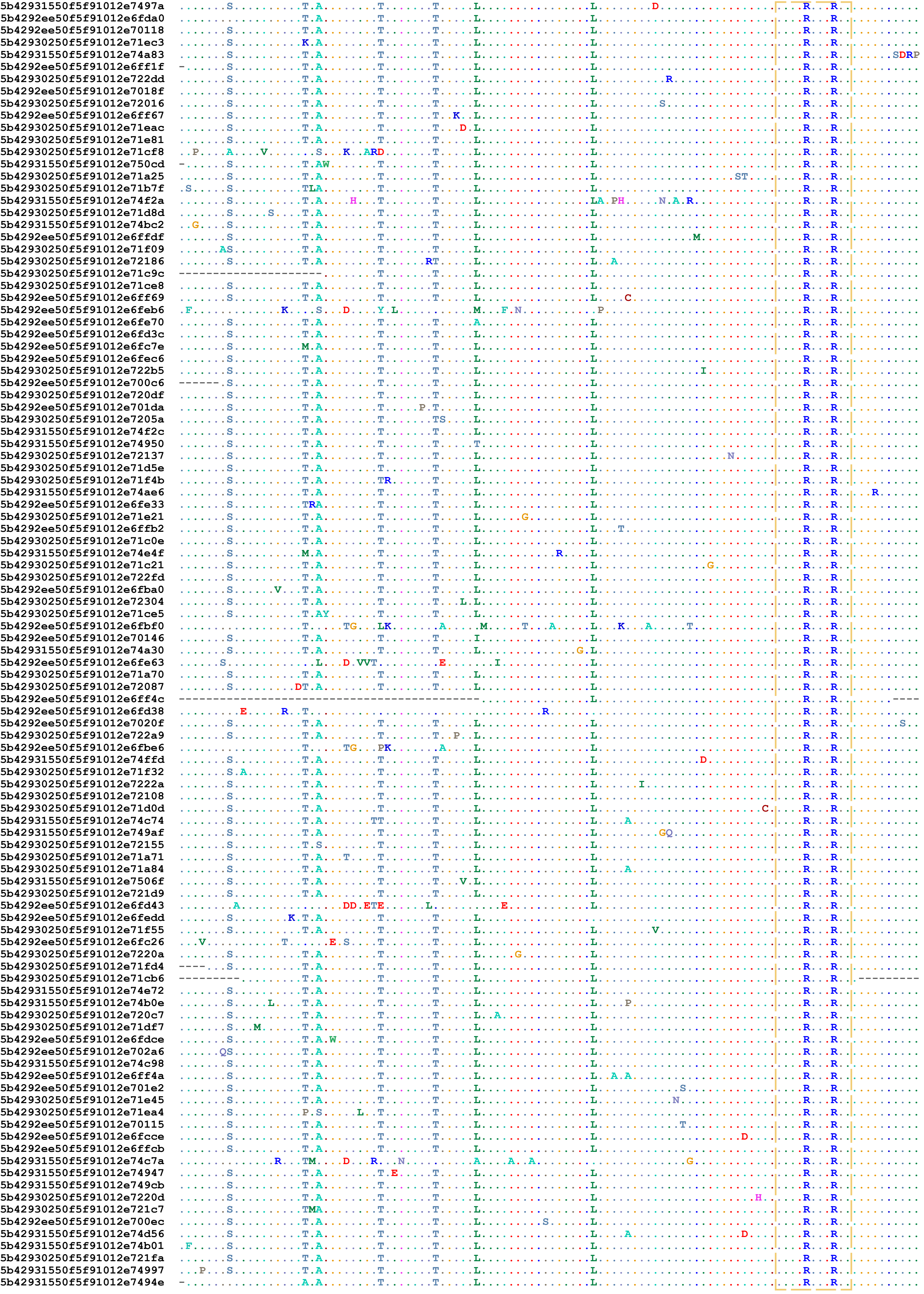
5b42931550f5f91012e7497a
5b4292ee50f5f91012e6fda0
5b4292ee50f5f91012e70118
5b42930250f5f91012e71ec3
5b42931550f5f91012e74a83
5b4292ee50f5f91012e6ff1f
5b42930250f5f91012e722dd
5b4292ee50f5f91012e7018f
5b42930250f5f91012e72016
5b4292ee50f5f91012e6ff67
5b42930250f5f91012e71eac
5b42930250f5f91012e71e81
5b42930250f5f91012e71cf8
5b42931550f5f91012e750cd
5b42930250f5f91012e71a25
5b42930250f5f91012e71b7f
5b42931550f5f91012e74f2a
5b42930250f5f91012e71d8d
5b42931550f5f91012e74bc2
5b4292ee50f5f91012e6ffdf
5b42930250f5f91012e71f09
5b42930250f5f91012e72186
5b42930250f5f91012e71c9c
5b42930250f5f91012e71ce8
5b4292ee50f5f91012e6ff69
5b4292ee50f5f91012e6feb6
5b4292ee50f5f91012e6fe70
5b4292ee50f5f91012e6fd3c
5b4292ee50f5f91012e6fc7e
5b4292ee50f5f91012e6fec6
5b42930250f5f91012e722b5
5b4292ee50f5f91012e700c6
5b42930250f5f91012e720df
5b4292ee50f5f91012e701da
5b42930250f5f91012e7205a
5b42931550f5f91012e74f2c
5b42931550f5f91012e74950
5b42930250f5f91012e72137
5b42930250f5f91012e71d5e
5b42930250f5f91012e71f4b
5b42931550f5f91012e74ae6
5b4292ee50f5f91012e6fe33
5b42930250f5f91012e71e21
5b4292ee50f5f91012e6ffb2
5b42930250f5f91012e71c0e
5b42931550f5f91012e74e4f
5b42930250f5f91012e71c21
5b42930250f5f91012e722fd
5b4292ee50f5f91012e6fba0
5b42930250f5f91012e72304
5b42930250f5f91012e71ce5
5b4292ee50f5f91012e6fbf0
5b4292ee50f5f91012e70146
5b42931550f5f91012e74a30
5b4292ee50f5f91012e6fe63
5b42930250f5f91012e71a70
5b42930250f5f91012e72087
5b4292ee50f5f91012e6ff4c
5b4292ee50f5f91012e6fd38
5b4292ee50f5f91012e7020f
5b42930250f5f91012e722a9
5b4292ee50f5f91012e6fbe6
5b42931550f5f91012e74ffd
5b42930250f5f91012e71f32
5b42930250f5f91012e7222a
5b42930250f5f91012e72108
5b42930250f5f91012e71d0d
5b42931550f5f91012e74c74
5b42931550f5f91012e749af
5b42930250f5f91012e72155
5b42930250f5f91012e71a71
5b42930250f5f91012e71a84
5b42931550f5f91012e7506f
5b42930250f5f91012e721d9
5b4292ee50f5f91012e6fd43
5b4292ee50f5f91012e6fedd
5b42930250f5f91012e71f55
5b4292ee50f5f91012e6fc26
5b42930250f5f91012e7220a
5b42930250f5f91012e71fd4
5b42930250f5f91012e71cb6
5b42931550f5f91012e74e72
5b42931550f5f91012e74b0e
5b42930250f5f91012e720c7
5b42930250f5f91012e71df7
5b4292ee50f5f91012e6fdce
5b4292ee50f5f91012e702a6
5b42931550f5f91012e74c98
5b4292ee50f5f91012e6ff4a
5b4292ee50f5f91012e701e2
5b42930250f5f91012e71e45
5b42930250f5f91012e71ea4
5b4292ee50f5f91012e70115
5b4292ee50f5f91012e6fcce
5b4292ee50f5f91012e6ffcb
5b42931550f5f91012e74c7a
5b42931550f5f91012e74947
5b42931550f5f91012e749cb
5b42930250f5f91012e7220d
5b42930250f5f91012e721c7
5b4292ee50f5f91012e700ec
5b42931550f5f91012e74d56
5b42931550f5f91012e74b01
5b42930250f5f91012e721fa
5b42931550f5f91012e74997
5b42931550f5f91012e7494e

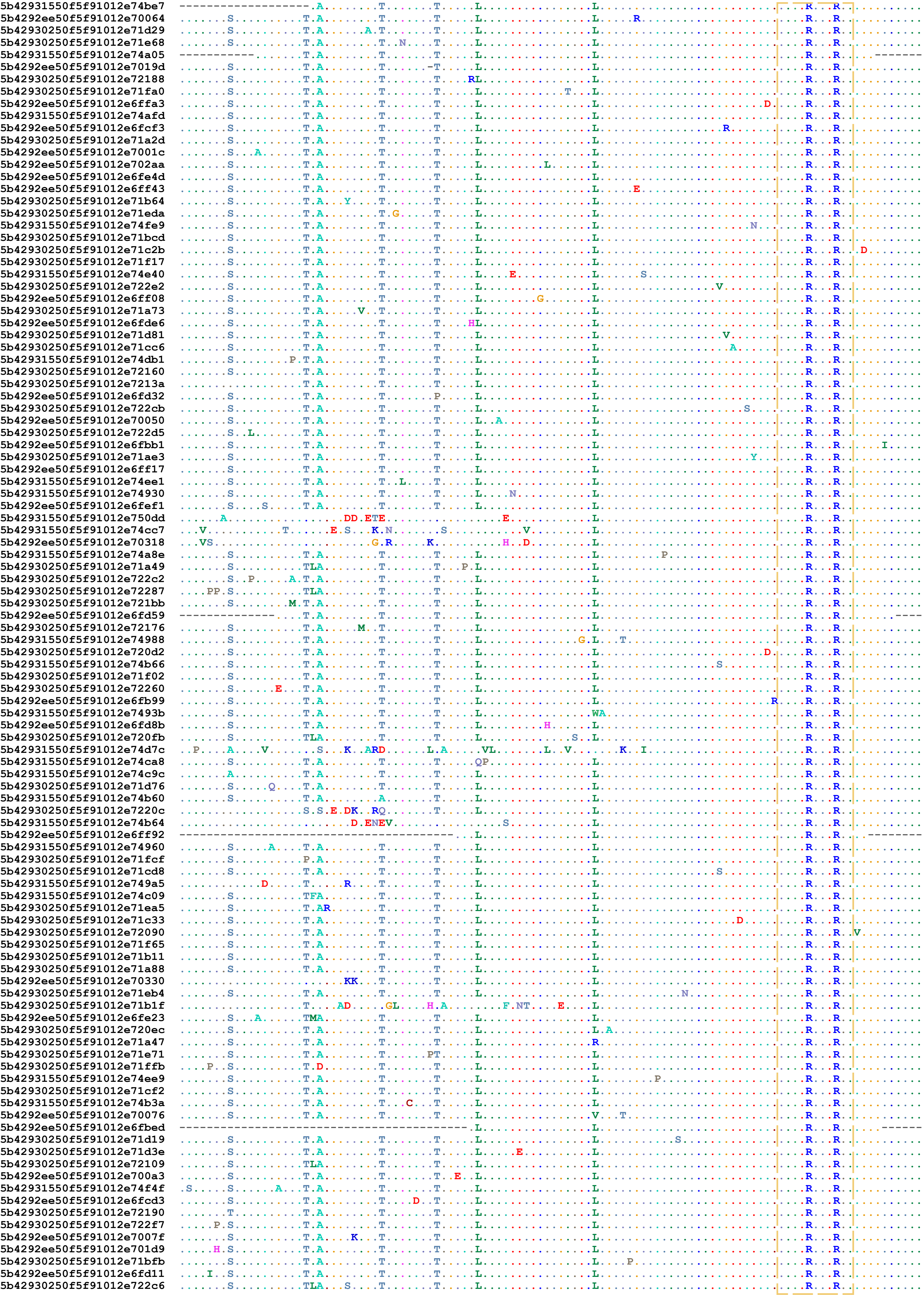
5b42931550f5f91012e74be7
5b4292ee50f5f91012e70064
5b42930250f5f91012e71d29
5b42930250f5f91012e71e68
5b42931550f5f91012e74a05
5b4292ee50f5f91012e7019d
5b42930250f5f91012e72188
5b42930250f5f91012e71fa0
5b4292ee50f5f91012e6ffa3
5b42931550f5f91012e74afd
5b4292ee50f5f91012e6fcf3
5b42930250f5f91012e71a2d
5b4292ee50f5f91012e7001c
5b4292ee50f5f91012e702aa
5b4292ee50f5f91012e6fe4d
5b4292ee50f5f91012e6ff43
5b42930250f5f91012e71b64
5b42930250f5f91012e71eda
5b42931550f5f91012e74fe9
5b42930250f5f91012e71bcd
5b42930250f5f91012e71c2b
5b42930250f5f91012e71f17
5b42931550f5f91012e74e40
5b42930250f5f91012e722e2
5b4292ee50f5f91012e6ff08
5b42930250f5f91012e71a73
5b4292ee50f5f91012e6fde6
5b42930250f5f91012e71d81
5b42930250f5f91012e71cc6
5b42931550f5f91012e74db1
5b42930250f5f91012e72160
5b42930250f5f91012e7213a
5b4292ee50f5f91012e6fd32
5b42930250f5f91012e722cb
5b4292ee50f5f91012e70050
5b42930250f5f91012e722d5
5b4292ee50f5f91012e6fbb1
5b42930250f5f91012e71ae3
5b4292ee50f5f91012e6ff17
5b42931550f5f91012e74ee1
5b42931550f5f91012e74930
5b4292ee50f5f91012e6fef1
5b42931550f5f91012e750dd
5b42931550f5f91012e74cc7
5b4292ee50f5f91012e70318
5b42931550f5f91012e74a8e
5b42930250f5f91012e71a49
5b42930250f5f91012e722c2
5b42930250f5f91012e72287
5b42930250f5f91012e721bb
5b4292ee50f5f91012e6fd59
5b42930250f5f91012e72176
5b42931550f5f91012e74988
5b42930250f5f91012e720d2
5b42931550f5f91012e74b66
5b42930250f5f91012e71f02
5b42930250f5f91012e72260
5b4292ee50f5f91012e6fb99
5b42931550f5f91012e7493b
5b4292ee50f5f91012e6fd8b
5b42930250f5f91012e720fb
5b42931550f5f91012e74d7c
5b42931550f5f91012e74ca8
5b42931550f5f91012e74c9c
5b42930250f5f91012e71d76
5b42931550f5f91012e74b60
5b42930250f5f91012e7220c
5b42931550f5f91012e74b64
5b4292ee50f5f91012e6ff92
5b42931550f5f91012e74960
5b42930250f5f91012e71fcf
5b42930250f5f91012e71cd8
5b42931550f5f91012e749a5
5b42931550f5f91012e74c09
5b42930250f5f91012e71ea5
5b42930250f5f91012e71c33
5b42930250f5f91012e72090
5b42930250f5f91012e71f65
5b42930250f5f91012e71b11
5b42930250f5f91012e71a88
5b4292ee50f5f91012e70330
5b42930250f5f91012e71eb4
5b42930250f5f91012e71b1f
5b4292ee50f5f91012e6fe23
5b42930250f5f91012e720ec
5b42930250f5f91012e71a47
5b42930250f5f91012e71e71
5b42930250f5f91012e71ffb
5b42931550f5f91012e74ee9
5b42930250f5f91012e71cf2
5b42931550f5f91012e74b3a
5b4292ee50f5f91012e70076
5b4292ee50f5f91012e6fbed
5b42930250f5f91012e71d19
5b42930250f5f91012e71d3e
5b42930250f5f91012e72109
5b4292ee50f5f91012e700a3
5b42931550f5f91012e74f4f
5b4292ee50f5f91012e6fcd3
5b42930250f5f91012e72190
5b42930250f5f91012e722f7
5b4292ee50f5f91012e7007f
5b4292ee50f5f91012e701d9
5b42930250f5f91012e71bfb
5b4292ee50f5f91012e6fd11
5b42930250f5f91012e722c6

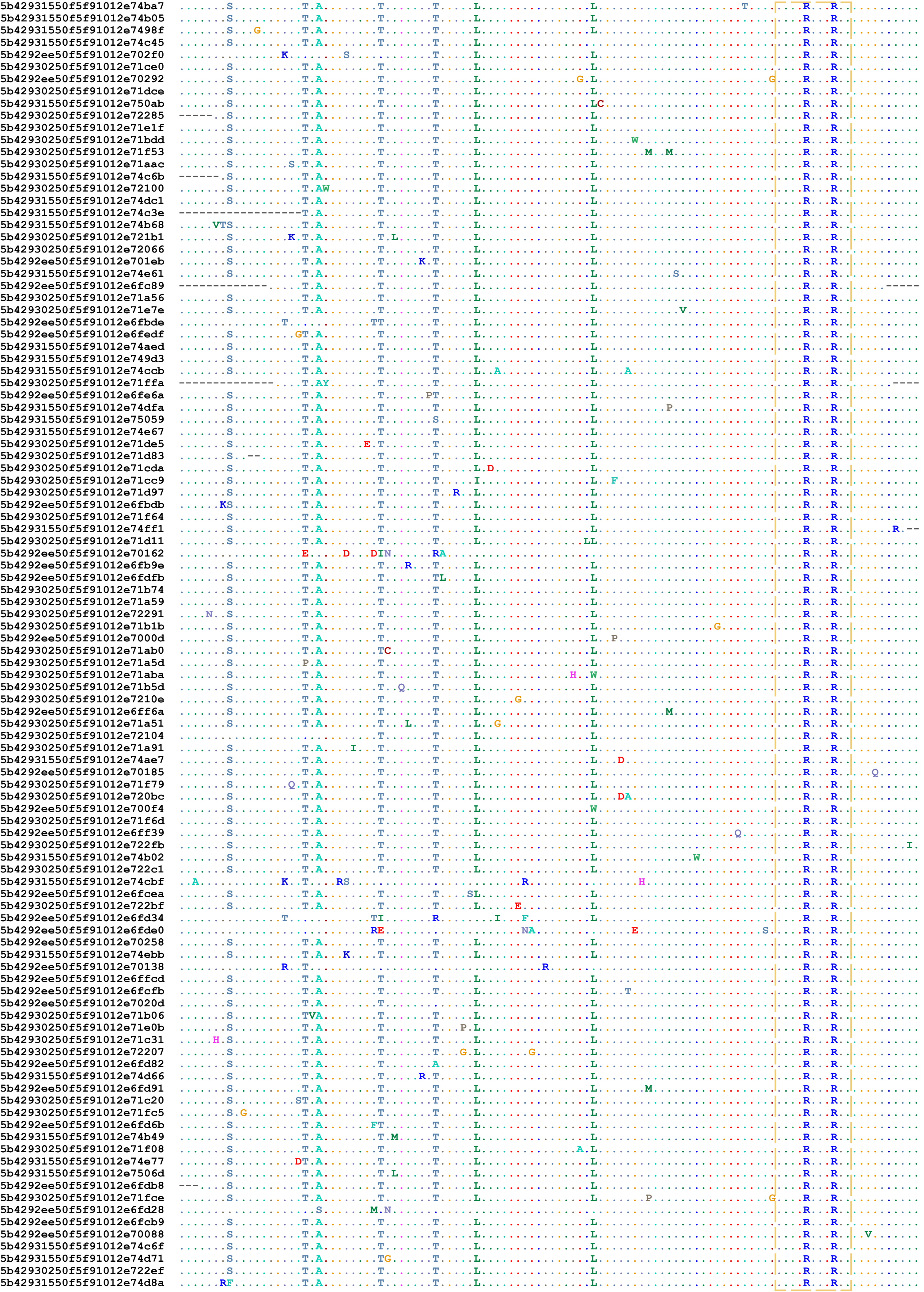
5b42931550f5f91012e74ba7
5b42931550f5f91012e74b05
5b42931550f5f91012e7498f
5b42931550f5f91012e74c45
5b4292ee50f5f91012e702f0
5b42930250f5f91012e71ce0
5b4292ee50f5f91012e70292
5b42930250f5f91012e71dce
5b42931550f5f91012e750ab
5b42930250f5f91012e72285
5b42930250f5f91012e71e1f
5b42930250f5f91012e71bdd
5b42930250f5f91012e71f53
5b42930250f5f91012e71aac
5b42931550f5f91012e74c6b
5b42930250f5f91012e72100
5b42931550f5f91012e74dc1
5b42931550f5f91012e74c3e
5b42931550f5f91012e74b68
5b42930250f5f91012e721b1
5b42930250f5f91012e72066
5b4292ee50f5f91012e701eb
5b42931550f5f91012e74e61
5b4292ee50f5f91012e6fc89
5b42930250f5f91012e71a56
5b42930250f5f91012e71e7e
5b4292ee50f5f91012e6fbde
5b4292ee50f5f91012e6fedf
5b42931550f5f91012e74aed
5b42931550f5f91012e749d3
5b42931550f5f91012e74ccb
5b42930250f5f91012e71ffa
5b4292ee50f5f91012e6fe6a
5b42931550f5f91012e74dfa
5b42931550f5f91012e75059
5b42931550f5f91012e74e67
5b42930250f5f91012e71de5
5b42930250f5f91012e71d83
5b42930250f5f91012e71cda
5b42930250f5f91012e71cc9
5b42930250f5f91012e71d97
5b4292ee50f5f91012e6fbdb
5b42930250f5f91012e71f64
5b42931550f5f91012e74ff1
5b42930250f5f91012e71d11
5b4292ee50f5f91012e70162
5b4292ee50f5f91012e6fb9e
5b4292ee50f5f91012e6fdfb
5b42930250f5f91012e71b74
5b42930250f5f91012e71a59
5b42930250f5f91012e72291
5b42930250f5f91012e71b1b
5b4292ee50f5f91012e7000d
5b42930250f5f91012e71ab0
5b42930250f5f91012e71a5d
5b42930250f5f91012e71aba
5b42930250f5f91012e71b5d
5b42930250f5f91012e7210e
5b4292ee50f5f91012e6ff6a
5b42930250f5f91012e71a51
5b42930250f5f91012e72104
5b42930250f5f91012e71a91
5b42931550f5f91012e74ae7
5b4292ee50f5f91012e70185
5b42930250f5f91012e71f79
5b42930250f5f91012e720bc
5b4292ee50f5f91012e700f4
5b42930250f5f91012e71f6d
5b4292ee50f5f91012e6ff39
5b42930250f5f91012e722fb
5b42931550f5f91012e74b02
5b42930250f5f91012e722c1
5b42931550f5f91012e74cbf
5b4292ee50f5f91012e6fcea
5b42930250f5f91012e722bf
5b4292ee50f5f91012e6fd34
5b4292ee50f5f91012e6fde0
5b4292ee50f5f91012e70258
5b42931550f5f91012e74ebb
5b4292ee50f5f91012e70138
5b4292ee50f5f91012e6ffcd
5b4292ee50f5f91012e6fcfb
5b4292ee50f5f91012e7020d
5b42930250f5f91012e71b06
5b42930250f5f91012e71e0b
5b42930250f5f91012e71c31
5b42930250f5f91012e72207
5b4292ee50f5f91012e6fd82
5b42931550f5f91012e74d66
5b4292ee50f5f91012e6fd91
5b42930250f5f91012e71c20
5b42930250f5f91012e71fc5
5b4292ee50f5f91012e6fd6b
5b42931550f5f91012e74b49
5b42930250f5f91012e71f08
5b42931550f5f91012e74e77
5b42931550f5f91012e7506d
5b4292ee50f5f91012e6fdb8
5b42930250f5f91012e71fce
5b4292ee50f5f91012e6fd28
5b4292ee50f5f91012e6fcb9
5b4292ee50f5f91012e70088
5b42931550f5f91012e74c6f
5b42931550f5f91012e74d71
5b42930250f5f91012e722ef
5b42931550f5f91012e74d8a

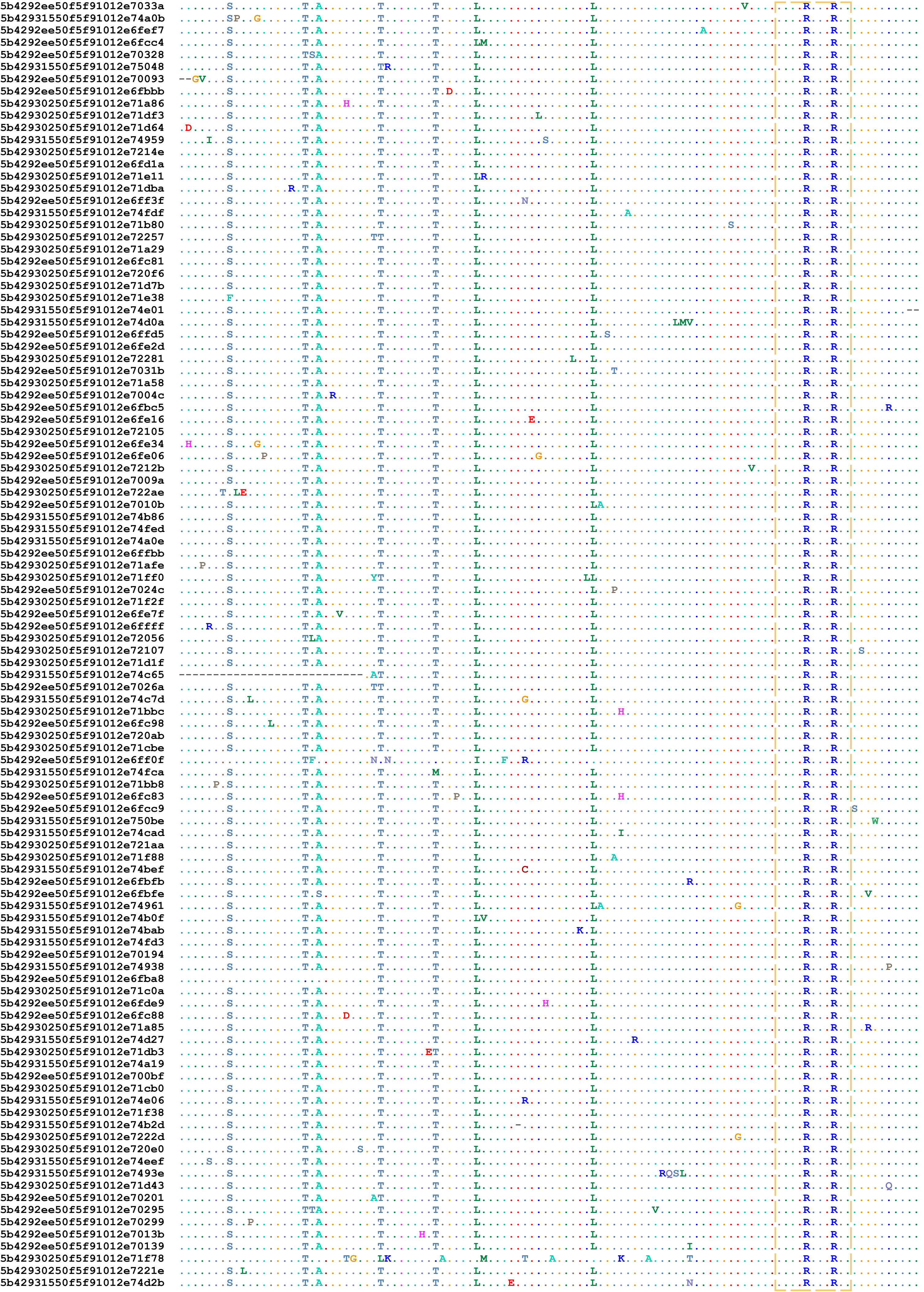
5b4292ee50f5f91012e7033a
5b42931550f5f91012e74a0b
5b4292ee50f5f91012e6fef7
5b4292ee50f5f91012e6fcc4
5b4292ee50f5f91012e70328
5b42931550f5f91012e75048
5b4292ee50f5f91012e70093
5b4292ee50f5f91012e6fbbb
5b42930250f5f91012e71a86
5b42930250f5f91012e71df3
5b42930250f5f91012e71d64
5b42931550f5f91012e74959
5b42930250f5f91012e7214e
5b4292ee50f5f91012e6fd1a
5b42930250f5f91012e71e11
5b42930250f5f91012e71dba
5b4292ee50f5f91012e6ff3f
5b42931550f5f91012e74fdf
5b42930250f5f91012e71b80
5b42930250f5f91012e72257
5b42930250f5f91012e71a29
5b4292ee50f5f91012e6fc81
5b42930250f5f91012e720f6
5b42930250f5f91012e71d7b
5b42930250f5f91012e71e38
5b42931550f5f91012e74e01
5b42931550f5f91012e74d0a
5b4292ee50f5f91012e6ffd5
5b4292ee50f5f91012e6fe2d
5b42930250f5f91012e72281
5b4292ee50f5f91012e7031b
5b42930250f5f91012e71a58
5b4292ee50f5f91012e7004c
5b4292ee50f5f91012e6fbc5
5b4292ee50f5f91012e6fe16
5b42930250f5f91012e72105
5b4292ee50f5f91012e6fe34
5b4292ee50f5f91012e6fe06
5b42930250f5f91012e7212b
5b4292ee50f5f91012e7009a
5b42930250f5f91012e722ae
5b4292ee50f5f91012e7010b
5b42931550f5f91012e74b86
5b42931550f5f91012e74fed
5b42931550f5f91012e74a0e
5b4292ee50f5f91012e6ffbb
5b42930250f5f91012e71afe
5b42930250f5f91012e71ff0
5b4292ee50f5f91012e7024c
5b42930250f5f91012e71f2f
5b4292ee50f5f91012e6fe7f
5b4292ee50f5f91012e6ffff
5b42930250f5f91012e72056
5b42930250f5f91012e72107
5b42930250f5f91012e71d1f
5b42931550f5f91012e74c65
5b4292ee50f5f91012e7026a
5b42931550f5f91012e74c7d
5b42930250f5f91012e71bbc
5b4292ee50f5f91012e6fc98
5b42930250f5f91012e720ab
5b42930250f5f91012e71cbe
5b4292ee50f5f91012e6ff0f
5b42931550f5f91012e74fca
5b42930250f5f91012e71bb8
5b4292ee50f5f91012e6fc83
5b4292ee50f5f91012e6fcc9
5b42931550f5f91012e750be
5b42931550f5f91012e74cad
5b42930250f5f91012e721aa
5b42930250f5f91012e71f88
5b42931550f5f91012e74bef
5b4292ee50f5f91012e6fbfb
5b4292ee50f5f91012e6fbfe
5b42931550f5f91012e74961
5b42931550f5f91012e74b0f
5b42931550f5f91012e74bab
5b42931550f5f91012e74fd3
5b4292ee50f5f91012e70194
5b42931550f5f91012e74938
5b4292ee50f5f91012e6fba8
5b42930250f5f91012e71c0a
5b4292ee50f5f91012e6fde9
5b4292ee50f5f91012e6fc88
5b42930250f5f91012e71a85
5b42931550f5f91012e74d27
5b42930250f5f91012e71db3
5b42931550f5f91012e74a19
5b4292ee50f5f91012e700bf
5b42930250f5f91012e71cb0
5b42931550f5f91012e74e06
5b42930250f5f91012e71f38
5b42931550f5f91012e74b2d
5b42930250f5f91012e7222d
5b42930250f5f91012e720e0
5b42931550f5f91012e74eef
5b42931550f5f91012e7493e
5b42930250f5f91012e71d43
5b4292ee50f5f91012e70201
5b4292ee50f5f91012e70295
5b4292ee50f5f91012e70299
5b4292ee50f5f91012e7013b
5b4292ee50f5f91012e70139
5b42930250f5f91012e71f78
5b42930250f5f91012e7221e
5b42931550f5f91012e74d2b

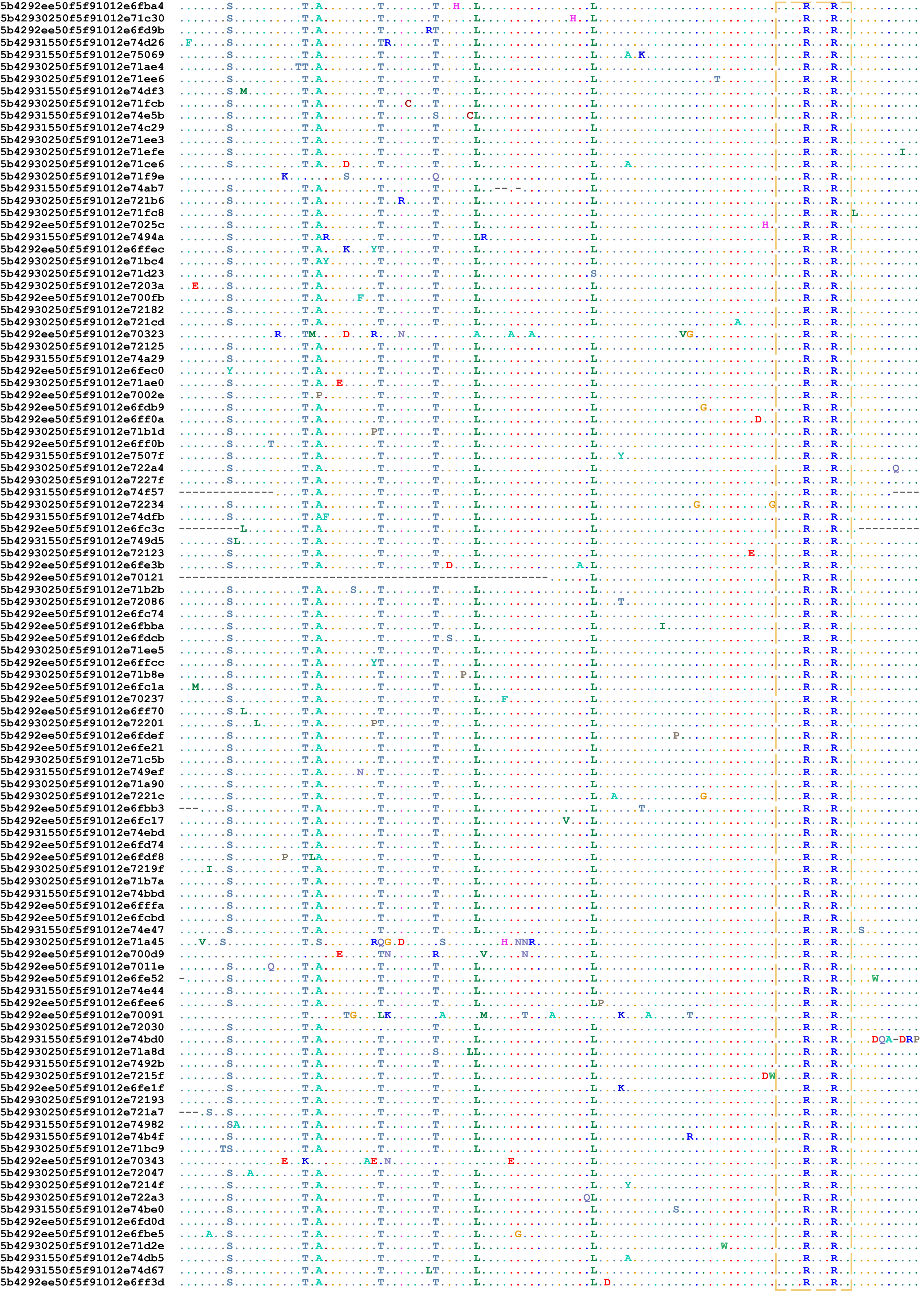
5b4292ee50f5f91012e6fba4
5b42930250f5f91012e71c30
5b4292ee50f5f91012e6fd9b
5b42931550f5f91012e74d26
5b42931550f5f91012e75069
5b42930250f5f91012e71ae4
5b42930250f5f91012e71ee6
5b42931550f5f91012e74df3
5b42930250f5f91012e71fcb
5b42931550f5f91012e74e5b
5b42931550f5f91012e74c29
5b42930250f5f91012e71ee3
5b42930250f5f91012e71efe
5b42930250f5f91012e71ce6
5b42930250f5f91012e71f9e
5b42931550f5f91012e74ab7
5b42930250f5f91012e721b6
5b42930250f5f91012e71fc8
5b4292ee50f5f91012e7025c
5b42931550f5f91012e7494a
5b4292ee50f5f91012e6ffec
5b42930250f5f91012e71bc4
5b42930250f5f91012e71d23
5b42930250f5f91012e7203a
5b4292ee50f5f91012e700fb
5b42930250f5f91012e72182
5b42930250f5f91012e721cd
5b4292ee50f5f91012e70323
5b42930250f5f91012e72125
5b42931550f5f91012e74a29
5b4292ee50f5f91012e6fec0
5b42930250f5f91012e71ae0
5b4292ee50f5f91012e7002e
5b4292ee50f5f91012e6fdb9
5b4292ee50f5f91012e6ff0a
5b42930250f5f91012e71b1d
5b4292ee50f5f91012e6ff0b
5b42931550f5f91012e7507f
5b42930250f5f91012e722a4
5b42930250f5f91012e7227f
5b42931550f5f91012e74f57
5b42930250f5f91012e72234
5b42931550f5f91012e74dfb
5b4292ee50f5f91012e6fc3c
5b42931550f5f91012e749d5
5b42930250f5f91012e72123
5b4292ee50f5f91012e6fe3b
5b4292ee50f5f91012e70121
5b42930250f5f91012e71b2b
5b42930250f5f91012e72086
5b4292ee50f5f91012e6fc74
5b4292ee50f5f91012e6fbba
5b4292ee50f5f91012e6fdcb
5b42930250f5f91012e71ee5
5b4292ee50f5f91012e6ffcc
5b42930250f5f91012e71b8e
5b4292ee50f5f91012e6fc1a
5b4292ee50f5f91012e70237
5b4292ee50f5f91012e6ff70
5b42930250f5f91012e72201
5b4292ee50f5f91012e6fdef
5b4292ee50f5f91012e6fe21
5b42930250f5f91012e71c5b
5b42931550f5f91012e749ef
5b42930250f5f91012e71a90
5b42930250f5f91012e7221c
5b4292ee50f5f91012e6fbb3
5b4292ee50f5f91012e6fc17
5b42931550f5f91012e74ebd
5b4292ee50f5f91012e6fd74
5b4292ee50f5f91012e6fdf8
5b42930250f5f91012e7219f
5b42930250f5f91012e71b7a
5b42931550f5f91012e74bbd
5b4292ee50f5f91012e6fffa
5b4292ee50f5f91012e6fcbd
5b42931550f5f91012e74e47
5b42930250f5f91012e71a45
5b4292ee50f5f91012e700d9
5b4292ee50f5f91012e7011e
5b4292ee50f5f91012e6fe52
5b42931550f5f91012e74e44
5b4292ee50f5f91012e6fee6
5b4292ee50f5f91012e70091
5b42930250f5f91012e72030
5b42931550f5f91012e74bd0
5b42930250f5f91012e71a8d
5b42931550f5f91012e7492b
5b42930250f5f91012e7215f
5b4292ee50f5f91012e6fe1f
5b42930250f5f91012e72193
5b42930250f5f91012e721a7
5b42931550f5f91012e74982
5b42931550f5f91012e74b4f
5b42930250f5f91012e71bc9
5b4292ee50f5f91012e70343
5b42930250f5f91012e72047
5b42930250f5f91012e7214f
5b42930250f5f91012e722a3
5b42931550f5f91012e74be0
5b4292ee50f5f91012e6fd0d
5b4292ee50f5f91012e6fbe5
5b42930250f5f91012e71d2e
5b42931550f5f91012e74db5
5b42931550f5f91012e74d67
5b4292ee50f5f91012e6ff3d

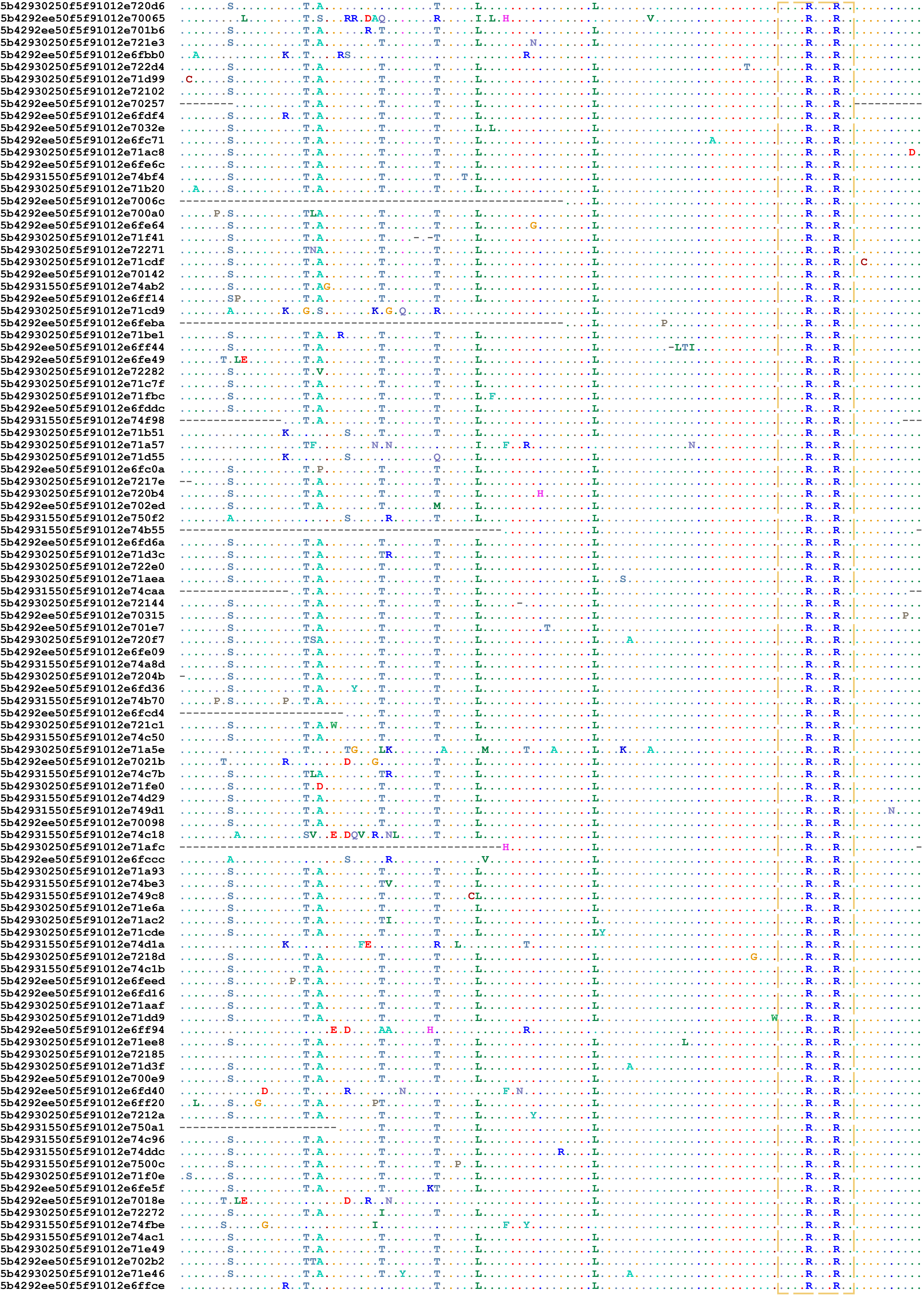
5b42930250f5f91012e720d6
5b4292ee50f5f91012e70065
5b4292ee50f5f91012e701b6
5b42930250f5f91012e721e3
5b4292ee50f5f91012e6fbb0
5b42930250f5f91012e722d4
5b42930250f5f91012e71d99
5b42930250f5f91012e72102
5b4292ee50f5f91012e70257
5b4292ee50f5f91012e6fdf4
5b4292ee50f5f91012e7032e
5b4292ee50f5f91012e6fc71
5b42930250f5f91012e71ac8
5b4292ee50f5f91012e6fe6c
5b42931550f5f91012e74bf4
5b42930250f5f91012e71b20
5b4292ee50f5f91012e7006c
5b4292ee50f5f91012e700a0
5b4292ee50f5f91012e6fe64
5b42930250f5f91012e71f41
5b42930250f5f91012e72271
5b42930250f5f91012e71cdf
5b4292ee50f5f91012e70142
5b42931550f5f91012e74ab2
5b4292ee50f5f91012e6ff14
5b42930250f5f91012e71cd9
5b4292ee50f5f91012e6feba
5b42930250f5f91012e71be1
5b4292ee50f5f91012e6ff44
5b4292ee50f5f91012e6fe49
5b42930250f5f91012e72282
5b42930250f5f91012e71c7f
5b42930250f5f91012e71fbc
5b4292ee50f5f91012e6fddc
5b42931550f5f91012e74f98
5b42930250f5f91012e71b51
5b42930250f5f91012e71a57
5b42930250f5f91012e71d55
5b4292ee50f5f91012e6fc0a
5b42930250f5f91012e7217e
5b42930250f5f91012e720b4
5b4292ee50f5f91012e702ed
5b42931550f5f91012e750f2
5b42931550f5f91012e74b55
5b4292ee50f5f91012e6fd6a
5b42930250f5f91012e71d3c
5b42930250f5f91012e722e0
5b42930250f5f91012e71aea
5b42931550f5f91012e74caa
5b42930250f5f91012e72144
5b4292ee50f5f91012e70315
5b4292ee50f5f91012e701e7
5b42930250f5f91012e720f7
5b4292ee50f5f91012e6fe09
5b42931550f5f91012e74a8d
5b42930250f5f91012e7204b
5b4292ee50f5f91012e6fd36
5b42931550f5f91012e74b70
5b4292ee50f5f91012e6fcd4
5b42930250f5f91012e721c1
5b42931550f5f91012e74c50
5b42930250f5f91012e71a5e
5b4292ee50f5f91012e7021b
5b42931550f5f91012e74c7b
5b42930250f5f91012e71fe0
5b42931550f5f91012e74d29
5b42931550f5f91012e749d1
5b4292ee50f5f91012e70098
5b42931550f5f91012e74c18
5b42930250f5f91012e71afc
5b4292ee50f5f91012e6fccc
5b42930250f5f91012e71a93
5b42931550f5f91012e74be3
5b42931550f5f91012e749c8
5b42930250f5f91012e71e6a
5b42930250f5f91012e71ac2
5b42930250f5f91012e71cde
5b42931550f5f91012e74d1a
5b42930250f5f91012e7218d
5b42931550f5f91012e74c1b
5b4292ee50f5f91012e6feed
5b4292ee50f5f91012e6fd16
5b42930250f5f91012e71aaf
5b42930250f5f91012e71dd9
5b4292ee50f5f91012e6ff94
5b42930250f5f91012e71ee8
5b42930250f5f91012e72185
5b42930250f5f91012e71d3f
5b4292ee50f5f91012e700e9
5b4292ee50f5f91012e6fd40
5b4292ee50f5f91012e6ff20
5b42930250f5f91012e7212a
5b42931550f5f91012e750a1
5b42931550f5f91012e74c96
5b42931550f5f91012e74ddc
5b42931550f5f91012e7500c
5b42930250f5f91012e71f0e
5b4292ee50f5f91012e6fe5f
5b4292ee50f5f91012e7018e
5b42930250f5f91012e72272
5b42931550f5f91012e74fbe
5b42931550f5f91012e74ac1
5b42930250f5f91012e71e49
5b4292ee50f5f91012e702b2
5b42930250f5f91012e71e46
5b4292ee50f5f91012e6ffce

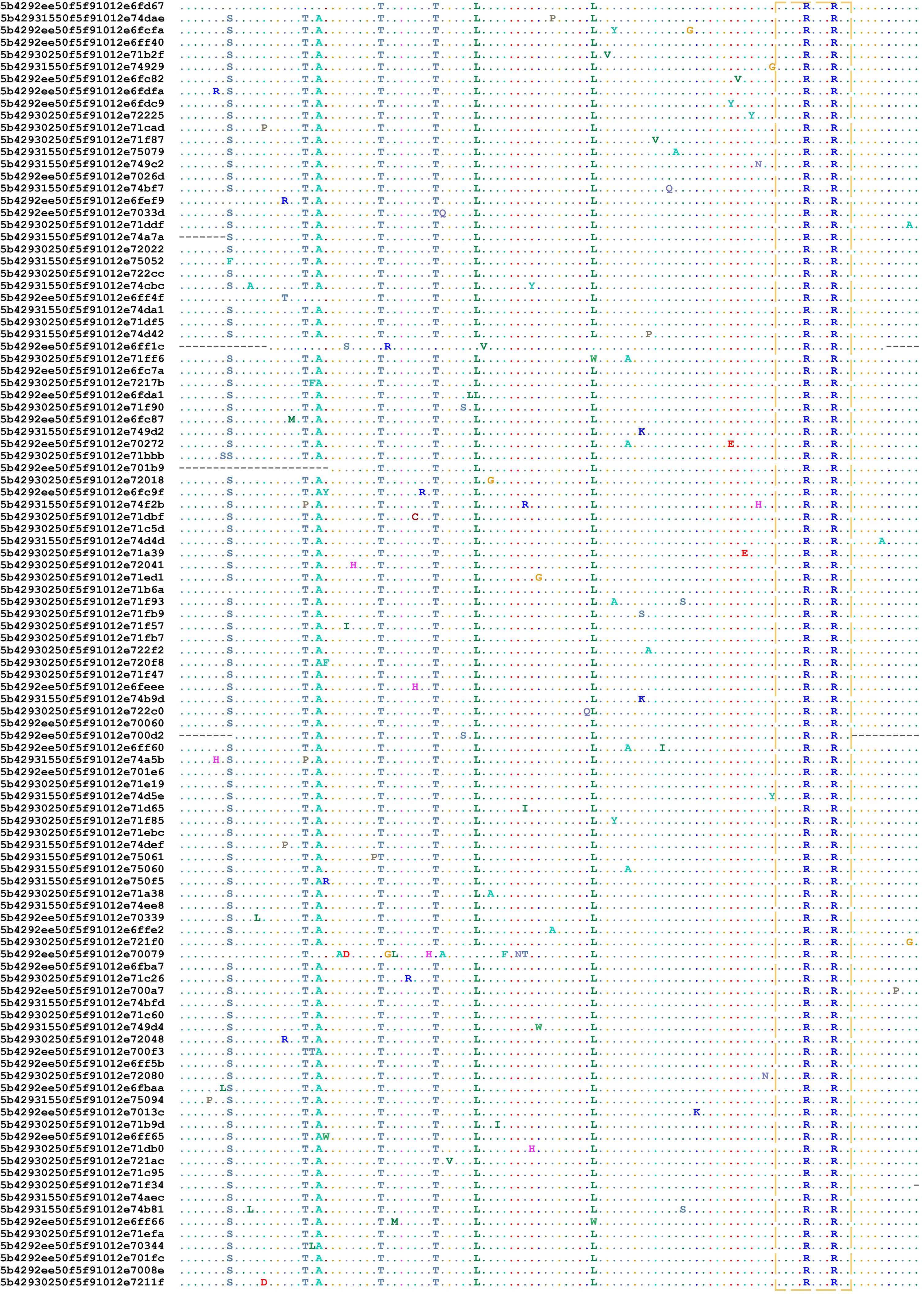
5b4292ee50f5f91012e6fd67
5b42931550f5f91012e74dae
5b4292ee50f5f91012e6fcfa
5b4292ee50f5f91012e6ff40
5b42930250f5f91012e71b2f
5b42931550f5f91012e74929
5b4292ee50f5f91012e6fc82
5b4292ee50f5f91012e6fdfa
5b4292ee50f5f91012e6fdc9
5b42930250f5f91012e72225
5b42930250f5f91012e71cad
5b42930250f5f91012e71f87
5b42931550f5f91012e75079
5b42931550f5f91012e749c2
5b4292ee50f5f91012e7026d
5b42931550f5f91012e74bf7
5b4292ee50f5f91012e6fef9
5b4292ee50f5f91012e7033d
5b42930250f5f91012e71ddf
5b42931550f5f91012e74a7a
5b42930250f5f91012e72022
5b42931550f5f91012e75052
5b42930250f5f91012e722cc
5b42931550f5f91012e74cbc
5b4292ee50f5f91012e6ff4f
5b42931550f5f91012e74da1
5b42930250f5f91012e71df5
5b42931550f5f91012e74d42
5b4292ee50f5f91012e6ff1c
5b42930250f5f91012e71ff6
5b4292ee50f5f91012e6fc7a
5b42930250f5f91012e7217b
5b4292ee50f5f91012e6fda1
5b42930250f5f91012e71f90
5b4292ee50f5f91012e6fc87
5b42931550f5f91012e749d2
5b4292ee50f5f91012e70272
5b42930250f5f91012e71bbb
5b4292ee50f5f91012e701b9
5b42930250f5f91012e72018
5b4292ee50f5f91012e6fc9f
5b42931550f5f91012e74f2b
5b42930250f5f91012e71dbf
5b42930250f5f91012e71c5d
5b42931550f5f91012e74d4d
5b42930250f5f91012e71a39
5b42930250f5f91012e72041
5b42930250f5f91012e71ed1
5b42930250f5f91012e71b6a
5b42930250f5f91012e71f93
5b42930250f5f91012e71fb9
5b42930250f5f91012e71f57
5b42930250f5f91012e71fb7
5b42930250f5f91012e722f2
5b42930250f5f91012e720f8
5b42930250f5f91012e71f47
5b4292ee50f5f91012e6feee
5b42931550f5f91012e74b9d
5b42930250f5f91012e722c0
5b4292ee50f5f91012e70060
5b4292ee50f5f91012e700d2
5b4292ee50f5f91012e6ff60
5b42931550f5f91012e74a5b
5b4292ee50f5f91012e701e6
5b42930250f5f91012e71e19
5b42931550f5f91012e74d5e
5b42930250f5f91012e71d65
5b42930250f5f91012e71f85
5b42930250f5f91012e71ebc
5b42931550f5f91012e74def
5b42931550f5f91012e75061
5b42931550f5f91012e75060
5b42931550f5f91012e750f5
5b42930250f5f91012e71a38
5b42931550f5f91012e74ee8
5b4292ee50f5f91012e70339
5b4292ee50f5f91012e6ffe2
5b42930250f5f91012e721f0
5b4292ee50f5f91012e70079
5b4292ee50f5f91012e6fba7
5b42930250f5f91012e71c26
5b4292ee50f5f91012e700a7
5b42931550f5f91012e74bfd
5b42930250f5f91012e71c60
5b42931550f5f91012e749d4
5b42930250f5f91012e72048
5b4292ee50f5f91012e700f3
5b4292ee50f5f91012e6ff5b
5b42930250f5f91012e72080
5b4292ee50f5f91012e6fbaa
5b42931550f5f91012e75094
5b4292ee50f5f91012e7013c
5b42930250f5f91012e71b9d
5b4292ee50f5f91012e6ff65
5b42930250f5f91012e71db0
5b42930250f5f91012e721ac
5b42930250f5f91012e71c95
5b42930250f5f91012e71f34
5b42931550f5f91012e74aec
5b42931550f5f91012e74b81
5b4292ee50f5f91012e6ff66
5b42930250f5f91012e71efa
5b4292ee50f5f91012e70344
5b4292ee50f5f91012e701fc
5b4292ee50f5f91012e7008e
5b42930250f5f91012e7211f

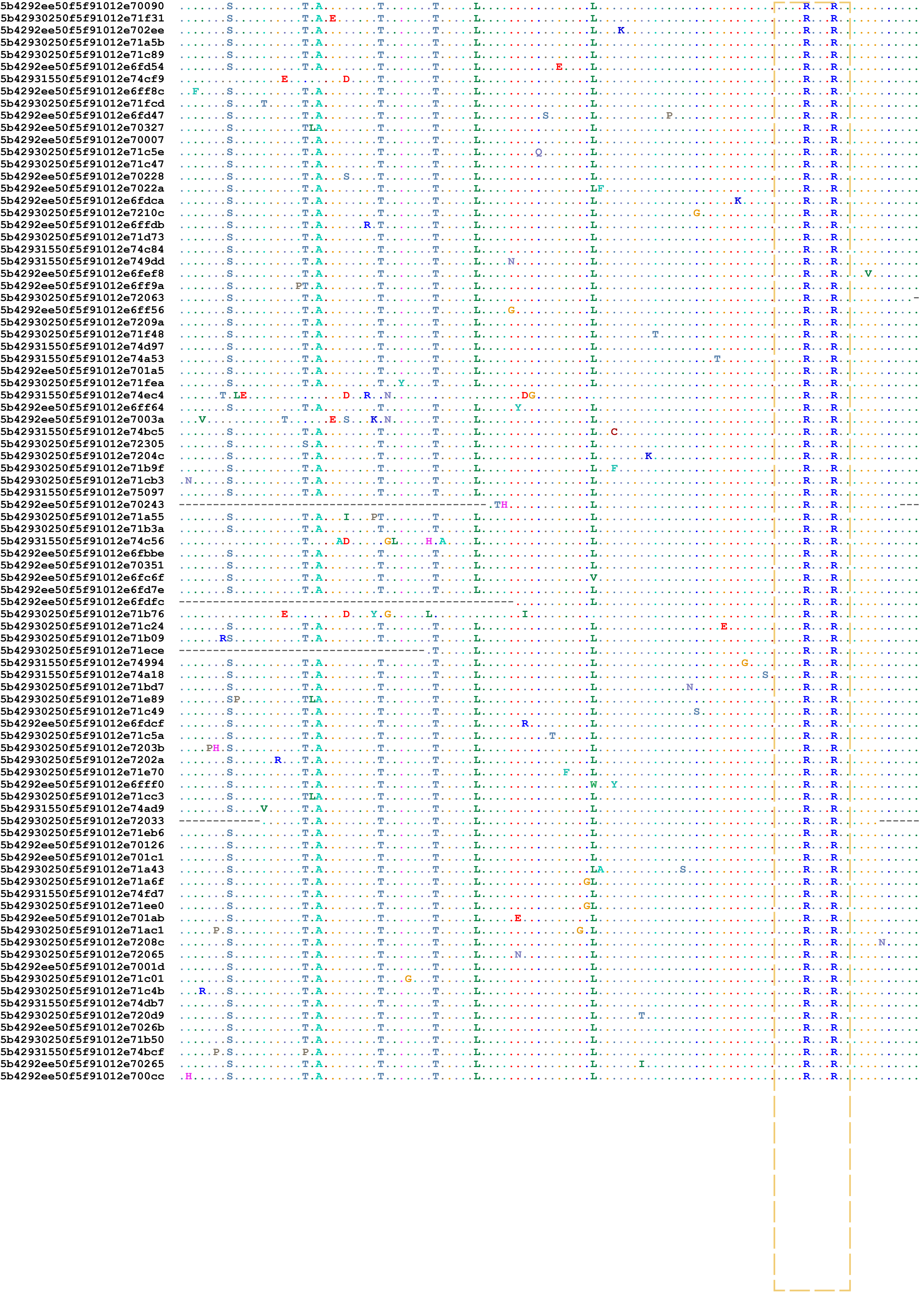

Supplement: S1 Fig — Somatic variants of the dominant clone were aligned to inferred germline genes to create a multiple sequence alignment. (PDF) [file pone.0235713.s003.pdf]
