# Supplementary material for: Diverse patterns of antibody variable gene repertoire disruption in patients with amyloid light chain (AL) amyloidosis
Source: PLoS One. 2020 Jul 7;15(7):e0235713. doi: 10.1371/journal.pone.0235713 (PMC7340310; doi:10.1371/journal.pone.0235713)
Supplement: S2 Fig — Somatic variants of the dominant clone were aligned to inferred germline genes to create a multiple sequence alignment. (PDF) [file pone.0235713.s004.pdf]

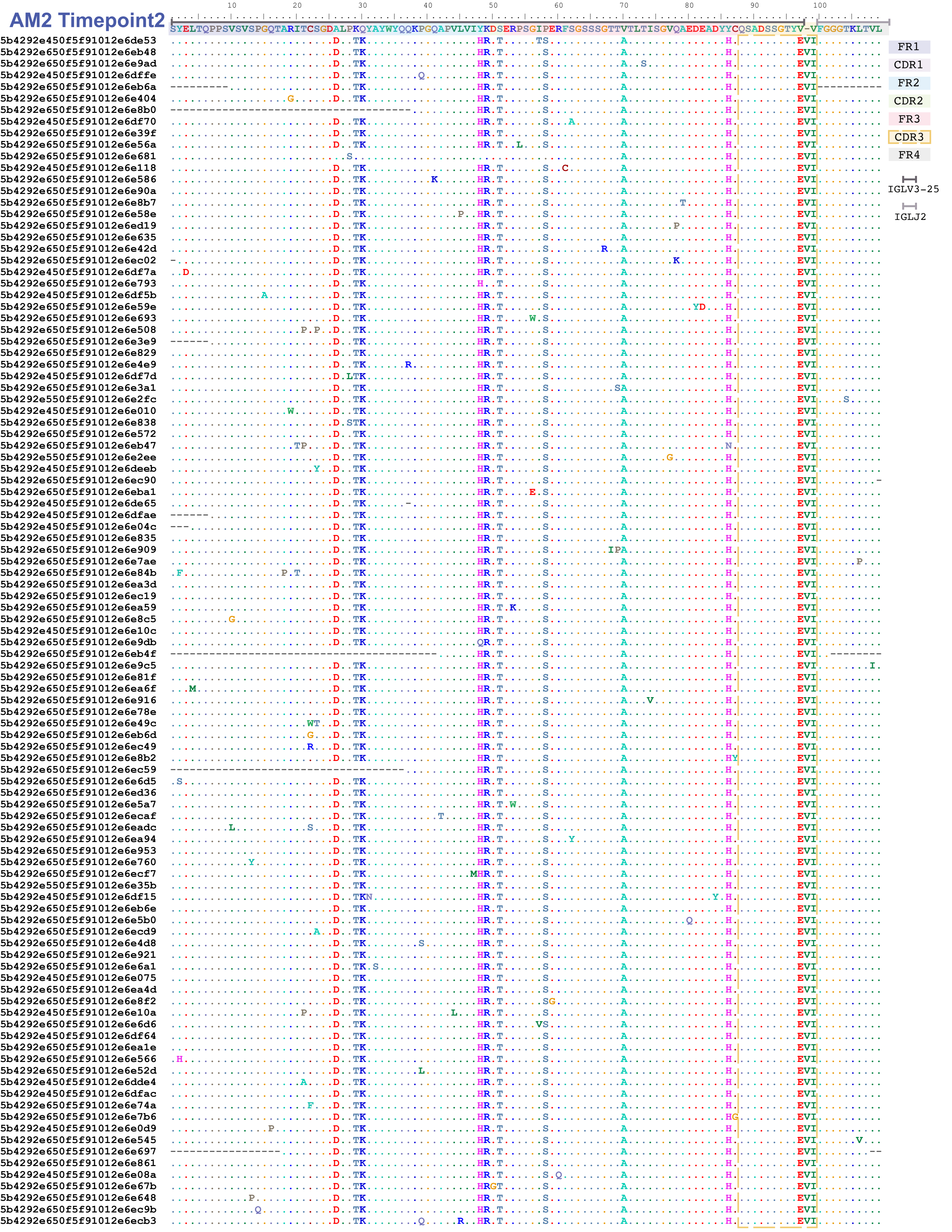

AM2 Timepoint2
SYELTQPPSVSVSPGQTARITCSGDALPKQYAYWYQQKPGQAPVLVIYKDSERPSGIPERFSGSSSGTTVTLTISGVQAEDEADYYCQSADSSGTYV-VFGGGTKLTVL
FR1
CDR1
FR2
CDR2
FR3
CDR3
FR4
IGLV3-25
IGLJ2

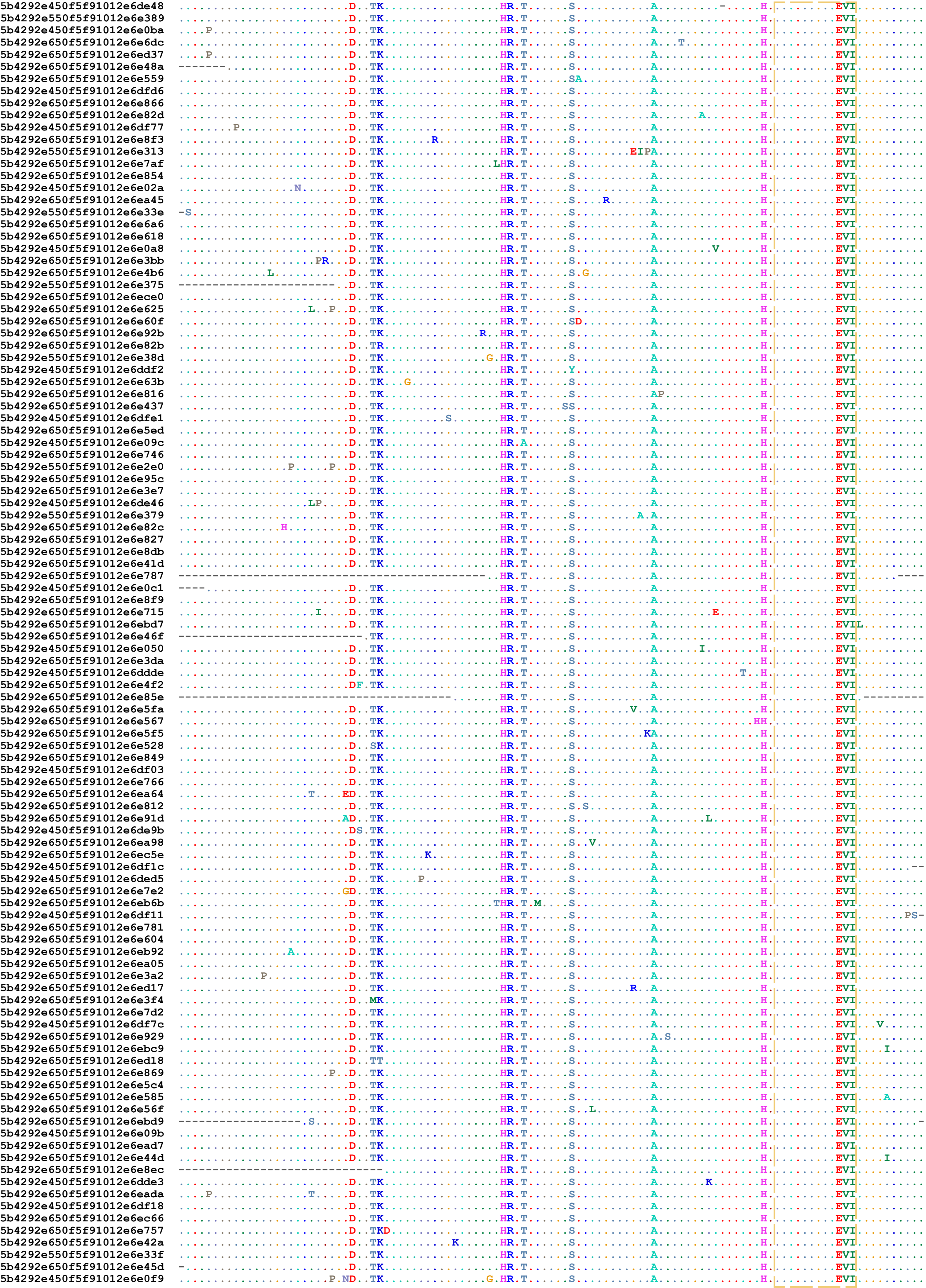

5b4292e450f5f91012e6de48
5b4292e550f5f91012e6e389
5b4292e450f5f91012e6e0ba
5b4292e650f5f91012e6e6dc
5b4292e650f5f91012e6ed37
5b4292e650f5f91012e6e48a
5b4292e650f5f91012e6e559
5b4292e450f5f91012e6dfd6
5b4292e650f5f91012e6e866
5b4292e650f5f91012e6e82d
5b4292e450f5f91012e6df77
5b4292e650f5f91012e6e8f3
5b4292e550f5f91012e6e313
5b4292e650f5f91012e6e7af
5b4292e650f5f91012e6e854
5b4292e450f5f91012e6e02a
5b4292e650f5f91012e6ea45
5b4292e550f5f91012e6e33e
5b4292e650f5f91012e6e6a6
5b4292e650f5f91012e6e618
5b4292e450f5f91012e6e0a8
5b4292e650f5f91012e6e3bb
5b4292e650f5f91012e6e4b6
5b4292e550f5f91012e6e375
5b4292e650f5f91012e6ece0
5b4292e650f5f91012e6e625
5b4292e650f5f91012e6e60f
5b4292e650f5f91012e6e92b
5b4292e650f5f91012e6e82b
5b4292e550f5f91012e6e38d
5b4292e450f5f91012e6ddf2
5b4292e650f5f91012e6e63b
5b4292e650f5f91012e6e816
5b4292e650f5f91012e6e437
5b4292e450f5f91012e6dfe1
5b4292e650f5f91012e6e5ed
5b4292e450f5f91012e6e09c
5b4292e650f5f91012e6e746
5b4292e550f5f91012e6e2e0
5b4292e650f5f91012e6e95c
5b4292e650f5f91012e6e3e7
5b4292e450f5f91012e6de46
5b4292e550f5f91012e6e379
5b4292e650f5f91012e6e82c
5b4292e650f5f91012e6e827
5b4292e650f5f91012e6e8db
5b4292e650f5f91012e6e41d
5b4292e650f5f91012e6e787
5b4292e450f5f91012e6e0c1
5b4292e650f5f91012e6e8f9
5b4292e650f5f91012e6e715
5b4292e650f5f91012e6ebd7
5b4292e650f5f91012e6e46f
5b4292e450f5f91012e6e050
5b4292e650f5f91012e6e3da
5b4292e450f5f91012e6ddde
5b4292e650f5f91012e6e4f2
5b4292e650f5f91012e6e85e
5b4292e650f5f91012e6e5fa
5b4292e650f5f91012e6e567
5b4292e650f5f91012e6e5f5
5b4292e650f5f91012e6e528
5b4292e650f5f91012e6e849
5b4292e450f5f91012e6df03
5b4292e650f5f91012e6e766
5b4292e650f5f91012e6ea64
5b4292e650f5f91012e6e812
5b4292e650f5f91012e6e91d
5b4292e450f5f91012e6de9b
5b4292e650f5f91012e6ea98
5b4292e650f5f91012e6ec5e
5b4292e450f5f91012e6df1c
5b4292e450f5f91012e6ded5
5b4292e650f5f91012e6e7e2
5b4292e650f5f91012e6eb6b
5b4292e450f5f91012e6df11
5b4292e650f5f91012e6e781
5b4292e650f5f91012e6e604
5b4292e650f5f91012e6eb92
5b4292e650f5f91012e6ea05
5b4292e650f5f91012e6e3a2
5b4292e650f5f91012e6ed17
5b4292e650f5f91012e6e3f4
5b4292e650f5f91012e6e7d2
5b4292e450f5f91012e6df7c
5b4292e650f5f91012e6e929
5b4292e650f5f91012e6ebc9
5b4292e650f5f91012e6ed18
5b4292e650f5f91012e6e869
5b4292e650f5f91012e6e5c4
5b4292e650f5f91012e6e585
5b4292e650f5f91012e6e56f
5b4292e650f5f91012e6ebd9
5b4292e450f5f91012e6e09b
5b4292e650f5f91012e6ead7
5b4292e650f5f91012e6e44d
5b4292e650f5f91012e6e8ec
5b4292e450f5f91012e6dde3
5b4292e650f5f91012e6eada
5b4292e450f5f91012e6df18
5b4292e650f5f91012e6ec66
5b4292e650f5f91012e6e757
5b4292e650f5f91012e6e42a
5b4292e550f5f91012e6e33f
5b4292e650f5f91012e6e45d
5b4292e450f5f91012e6e0f9

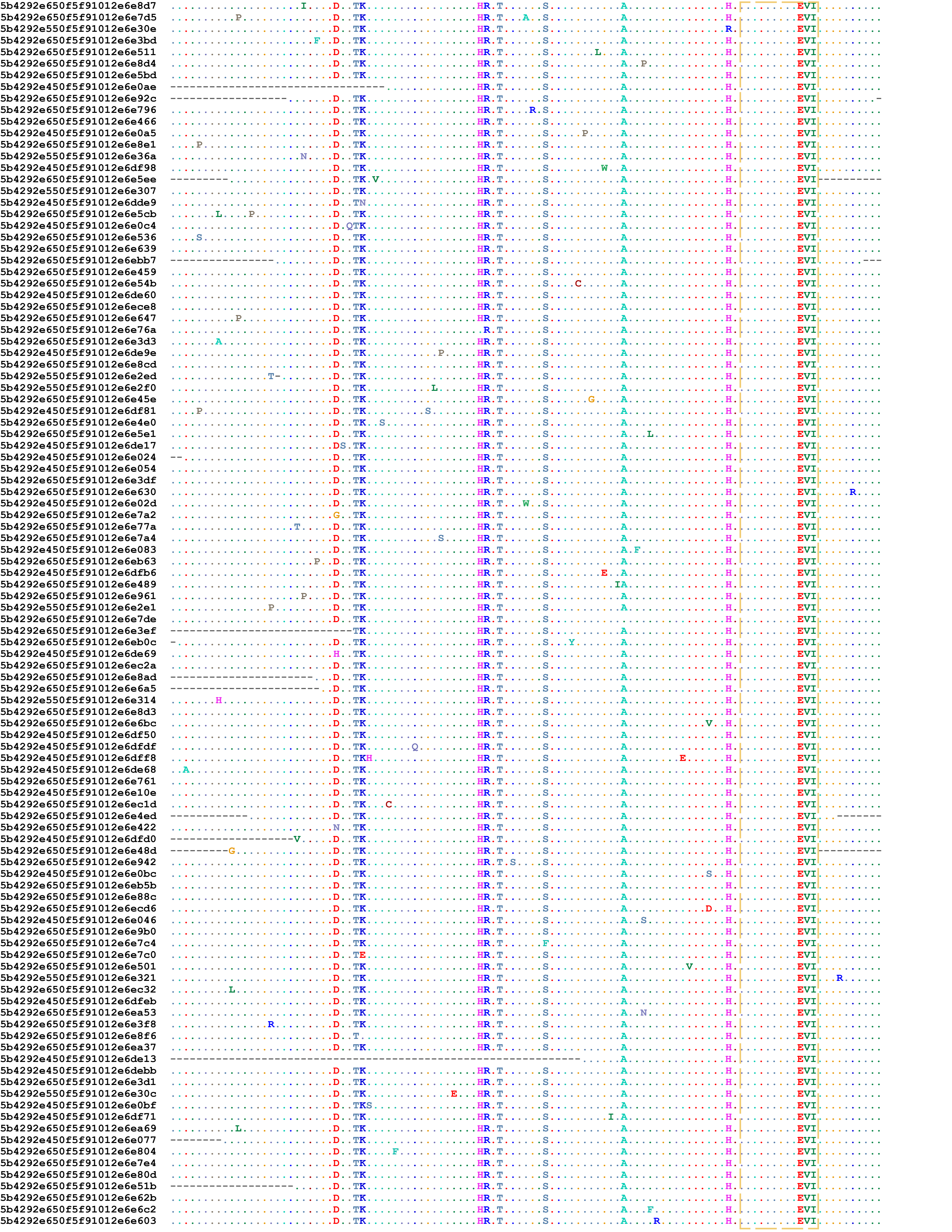
5b4292e650f5f91012e6e8d7
5b4292e650f5f91012e6e7d5
5b4292e550f5f91012e6e30e
5b4292e650f5f91012e6e3bd
5b4292e650f5f91012e6e511
5b4292e650f5f91012e6e8d4
5b4292e650f5f91012e6e5bd
5b4292e450f5f91012e6e0ae
5b4292e650f5f91012e6e92c
5b4292e650f5f91012e6e796
5b4292e650f5f91012e6e466
5b4292e450f5f91012e6e0a5
5b4292e650f5f91012e6e8e1
5b4292e550f5f91012e6e36a
5b4292e450f5f91012e6df98
5b4292e650f5f91012e6e5ee
5b4292e550f5f91012e6e307
5b4292e450f5f91012e6dde9
5b4292e650f5f91012e6e5cb
5b4292e450f5f91012e6e0c4
5b4292e650f5f91012e6e536
5b4292e650f5f91012e6e639
5b4292e650f5f91012e6ebb7
5b4292e650f5f91012e6e459
5b4292e650f5f91012e6e54b
5b4292e450f5f91012e6de60
5b4292e650f5f91012e6ece8
5b4292e650f5f91012e6e647
5b4292e650f5f91012e6e76a
5b4292e650f5f91012e6e3d3
5b4292e450f5f91012e6de9e
5b4292e650f5f91012e6e8cd
5b4292e550f5f91012e6e2ed
5b4292e550f5f91012e6e2f0
5b4292e650f5f91012e6e45e
5b4292e450f5f91012e6df81
5b4292e650f5f91012e6e4e0
5b4292e650f5f91012e6e5e1
5b4292e450f5f91012e6de17
5b4292e450f5f91012e6e024
5b4292e450f5f91012e6e054
5b4292e650f5f91012e6e3df
5b4292e650f5f91012e6e630
5b4292e450f5f91012e6e02d
5b4292e650f5f91012e6e7a2
5b4292e650f5f91012e6e77a
5b4292e650f5f91012e6e7a4
5b4292e450f5f91012e6e083
5b4292e650f5f91012e6eb63
5b4292e450f5f91012e6dfb6
5b4292e650f5f91012e6e489
5b4292e650f5f91012e6e961
5b4292e550f5f91012e6e2e1
5b4292e650f5f91012e6e7de
5b4292e650f5f91012e6e3ef
5b4292e650f5f91012e6eb0c
5b4292e450f5f91012e6de69
5b4292e650f5f91012e6ec2a
5b4292e650f5f91012e6e8ad
5b4292e650f5f91012e6e6a5
5b4292e550f5f91012e6e314
5b4292e650f5f91012e6e8d3
5b4292e650f5f91012e6e6bc
5b4292e450f5f91012e6df50
5b4292e450f5f91012e6dfdf
5b4292e450f5f91012e6dff8
5b4292e450f5f91012e6de68
5b4292e650f5f91012e6e761
5b4292e450f5f91012e6e10e
5b4292e650f5f91012e6ec1d
5b4292e650f5f91012e6e4ed
5b4292e650f5f91012e6e422
5b4292e450f5f91012e6dfd0
5b4292e650f5f91012e6e48d
5b4292e650f5f91012e6e942
5b4292e450f5f91012e6e0bc
5b4292e650f5f91012e6eb5b
5b4292e650f5f91012e6e88c
5b4292e650f5f91012e6ecd6
5b4292e450f5f91012e6e046
5b4292e650f5f91012e6e9b0
5b4292e650f5f91012e6e7c4
5b4292e650f5f91012e6e7c0
5b4292e650f5f91012e6e501
5b4292e550f5f91012e6e321
5b4292e650f5f91012e6ec32
5b4292e450f5f91012e6dfeb
5b4292e650f5f91012e6ea53
5b4292e650f5f91012e6e3f8
5b4292e650f5f91012e6e8f6
5b4292e650f5f91012e6ea37
5b4292e450f5f91012e6de13
5b4292e450f5f91012e6debb
5b4292e650f5f91012e6e3d1
5b4292e550f5f91012e6e30c
5b4292e450f5f91012e6e0bf
5b4292e450f5f91012e6df71
5b4292e650f5f91012e6ea69
5b4292e450f5f91012e6e077
5b4292e650f5f91012e6e804
5b4292e650f5f91012e6e7e4
5b4292e650f5f91012e6e80d
5b4292e650f5f91012e6e51b
5b4292e650f5f91012e6e62b
5b4292e650f5f91012e6e6c2
5b4292e650f5f91012e6e603

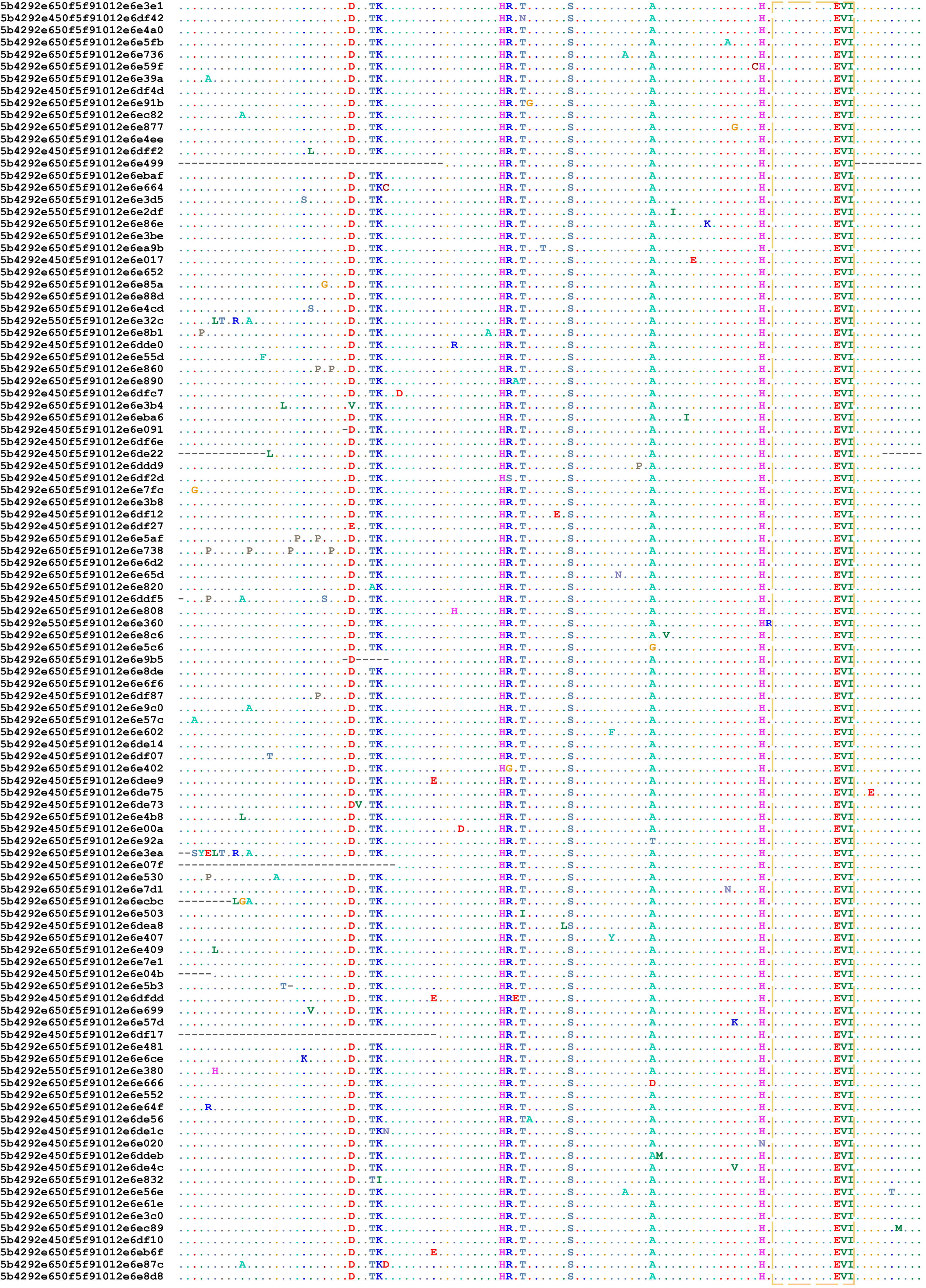

5b4292e650f5f91012e6e3e1
5b4292e450f5f91012e6df42
5b4292e650f5f91012e6e4a0
5b4292e650f5f91012e6e5fb
5b4292e650f5f91012e6e736
5b4292e650f5f91012e6e59f
5b4292e650f5f91012e6e39a
5b4292e450f5f91012e6df4d
5b4292e650f5f91012e6e91b
5b4292e650f5f91012e6ec82
5b4292e650f5f91012e6e877
5b4292e650f5f91012e6e4ee
5b4292e450f5f91012e6dff2
5b4292e650f5f91012e6e499
5b4292e650f5f91012e6ebaf
5b4292e650f5f91012e6e664
5b4292e650f5f91012e6e3d5
5b4292e550f5f91012e6e2df
5b4292e650f5f91012e6e86e
5b4292e650f5f91012e6e3be
5b4292e650f5f91012e6ea9b
5b4292e450f5f91012e6e017
5b4292e650f5f91012e6e652
5b4292e650f5f91012e6e85a
5b4292e650f5f91012e6e88d
5b4292e650f5f91012e6e4cd
5b4292e550f5f91012e6e32c
5b4292e650f5f91012e6e8b1
5b4292e450f5f91012e6dde0
5b4292e650f5f91012e6e55d
5b4292e650f5f91012e6e860
5b4292e650f5f91012e6e890
5b4292e450f5f91012e6dfc7
5b4292e650f5f91012e6e3b4
5b4292e650f5f91012e6eba6
5b4292e450f5f91012e6e091
5b4292e450f5f91012e6df6e
5b4292e450f5f91012e6de22
5b4292e450f5f91012e6ddd9
5b4292e450f5f91012e6df2d
5b4292e650f5f91012e6e7fc
5b4292e650f5f91012e6e3b8
5b4292e450f5f91012e6df12
5b4292e450f5f91012e6df27
5b4292e650f5f91012e6e5af
5b4292e650f5f91012e6e738
5b4292e650f5f91012e6e6d2
5b4292e650f5f91012e6e65d
5b4292e650f5f91012e6e820
5b4292e450f5f91012e6ddf5
5b4292e650f5f91012e6e808
5b4292e550f5f91012e6e360
5b4292e650f5f91012e6e8c6
5b4292e650f5f91012e6e5c6
5b4292e650f5f91012e6e9b5
5b4292e650f5f91012e6e8de
5b4292e650f5f91012e6e6f6
5b4292e450f5f91012e6df87
5b4292e650f5f91012e6e9c0
5b4292e650f5f91012e6e57c
5b4292e650f5f91012e6e602
5b4292e450f5f91012e6de14
5b4292e450f5f91012e6df07
5b4292e650f5f91012e6e402
5b4292e450f5f91012e6dee9
5b4292e450f5f91012e6de75
5b4292e450f5f91012e6de73
5b4292e650f5f91012e6e4b8
5b4292e450f5f91012e6e00a
5b4292e650f5f91012e6e92a
5b4292e650f5f91012e6e3ea
5b4292e450f5f91012e6e07f
5b4292e650f5f91012e6e530
5b4292e650f5f91012e6e7d1
5b4292e650f5f91012e6ecbc
5b4292e650f5f91012e6e503
5b4292e450f5f91012e6dea8
5b4292e650f5f91012e6e407
5b4292e650f5f91012e6e409
5b4292e650f5f91012e6e7e1
5b4292e450f5f91012e6e04b
5b4292e650f5f91012e6e5b3
5b4292e450f5f91012e6dfdd
5b4292e650f5f91012e6e699
5b4292e650f5f91012e6e57d
5b4292e450f5f91012e6df17
5b4292e650f5f91012e6e481
5b4292e650f5f91012e6e6ce
5b4292e550f5f91012e6e380
5b4292e650f5f91012e6e666
5b4292e650f5f91012e6e552
5b4292e650f5f91012e6e64f
5b4292e450f5f91012e6de56
5b4292e450f5f91012e6de1c
5b4292e450f5f91012e6e020
5b4292e450f5f91012e6ddeb
5b4292e450f5f91012e6de4c
5b4292e650f5f91012e6e832
5b4292e650f5f91012e6e56e
5b4292e650f5f91012e6e61e
5b4292e650f5f91012e6e3c0
5b4292e650f5f91012e6ec89
5b4292e450f5f91012e6df10
5b4292e650f5f91012e6eb6f
5b4292e650f5f91012e6e87c
5b4292e650f5f91012e6e8d8

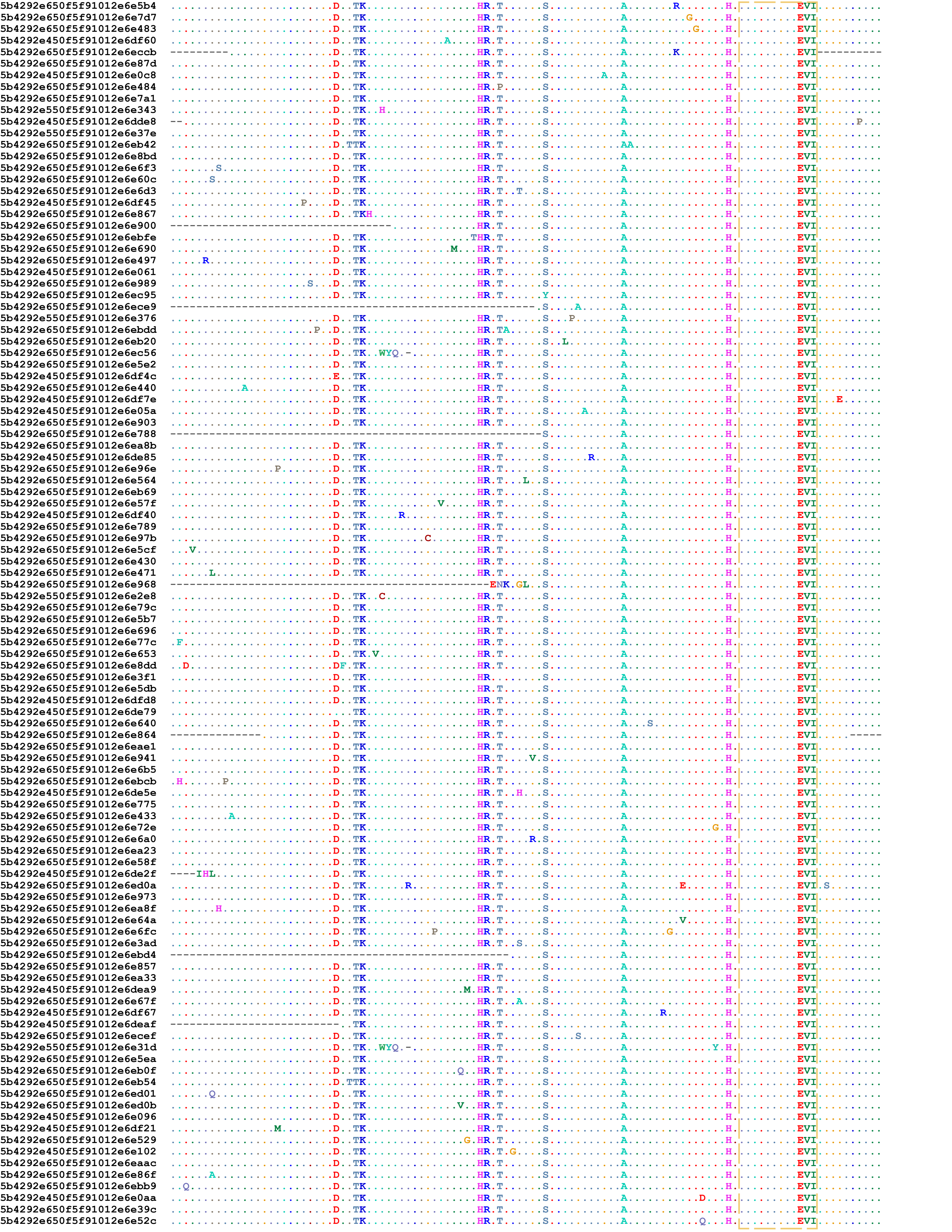
5b4292e650f5f91012e6e5b4
5b4292e650f5f91012e6e7d7
5b4292e650f5f91012e6e483
5b4292e450f5f91012e6df60
5b4292e650f5f91012e6eccb
5b4292e650f5f91012e6e87d
5b4292e450f5f91012e6e0c8
5b4292e650f5f91012e6e484
5b4292e650f5f91012e6e7a1
5b4292e550f5f91012e6e343
5b4292e450f5f91012e6dde8
5b4292e550f5f91012e6e37e
5b4292e650f5f91012e6eb42
5b4292e650f5f91012e6e8bd
5b4292e650f5f91012e6e6f3
5b4292e650f5f91012e6e60c
5b4292e650f5f91012e6e6d3
5b4292e450f5f91012e6df45
5b4292e650f5f91012e6e867
5b4292e650f5f91012e6e900
5b4292e650f5f91012e6ebfe
5b4292e650f5f91012e6e690
5b4292e650f5f91012e6e497
5b4292e450f5f91012e6e061
5b4292e650f5f91012e6e989
5b4292e650f5f91012e6ec95
5b4292e650f5f91012e6ece9
5b4292e550f5f91012e6e376
5b4292e650f5f91012e6ebdd
5b4292e650f5f91012e6eb20
5b4292e650f5f91012e6ec56
5b4292e650f5f91012e6e5e2
5b4292e450f5f91012e6df4c
5b4292e650f5f91012e6e440
5b4292e450f5f91012e6df7e
5b4292e450f5f91012e6e05a
5b4292e650f5f91012e6e903
5b4292e650f5f91012e6e788
5b4292e650f5f91012e6ea8b
5b4292e450f5f91012e6de85
5b4292e650f5f91012e6e96e
5b4292e650f5f91012e6e564
5b4292e650f5f91012e6eb69
5b4292e650f5f91012e6e57f
5b4292e450f5f91012e6df40
5b4292e650f5f91012e6e789
5b4292e650f5f91012e6e97b
5b4292e650f5f91012e6e5cf
5b4292e650f5f91012e6e430
5b4292e650f5f91012e6e471
5b4292e650f5f91012e6e968
5b4292e550f5f91012e6e2e8
5b4292e650f5f91012e6e79c
5b4292e650f5f91012e6e5b7
5b4292e650f5f91012e6e696
5b4292e650f5f91012e6e77c
5b4292e650f5f91012e6e653
5b4292e650f5f91012e6e8dd
5b4292e550f5f91012e6e3f1
5b4292e650f5f91012e6e5db
5b4292e450f5f91012e6dfd8
5b4292e450f5f91012e6de79
5b4292e650f5f91012e6e640
5b4292e650f5f91012e6e864
5b4292e650f5f91012e6eae1
5b4292e650f5f91012e6e941
5b4292e650f5f91012e6e6b5
5b4292e650f5f91012e6ebcb
5b4292e450f5f91012e6de5e
5b4292e650f5f91012e6e775
5b4292e650f5f91012e6e433
5b4292e650f5f91012e6e72e
5b4292e650f5f91012e6e6a0
5b4292e650f5f91012e6ea23
5b4292e650f5f91012e6e58f
5b4292e450f5f91012e6de2f
5b4292e650f5f91012e6ed0a
5b4292e650f5f91012e6e973
5b4292e650f5f91012e6ea8f
5b4292e650f5f91012e6e64a
5b4292e650f5f91012e6e6fc
5b4292e550f5f91012e6e3ad
5b4292e650f5f91012e6ebd4
5b4292e650f5f91012e6e857
5b4292e650f5f91012e6ea33
5b4292e450f5f91012e6dea9
5b4292e650f5f91012e6e67f
5b4292e450f5f91012e6df67
5b4292e450f5f91012e6deaf
5b4292e650f5f91012e6ecef
5b4292e550f5f91012e6e31d
5b4292e650f5f91012e6e5ea
5b4292e650f5f91012e6eb0f
5b4292e650f5f91012e6eb54
5b4292e650f5f91012e6ed01
5b4292e650f5f91012e6ed0b
5b4292e450f5f91012e6e096
5b4292e450f5f91012e6df21
5b4292e650f5f91012e6e529
5b4292e450f5f91012e6e102
5b4292e650f5f91012e6eaac
5b4292e650f5f91012e6e86f
5b4292e650f5f91012e6ebb9
5b4292e450f5f91012e6e0aa
5b4292e650f5f91012e6e39c
5b4292e650f5f91012e6e52c

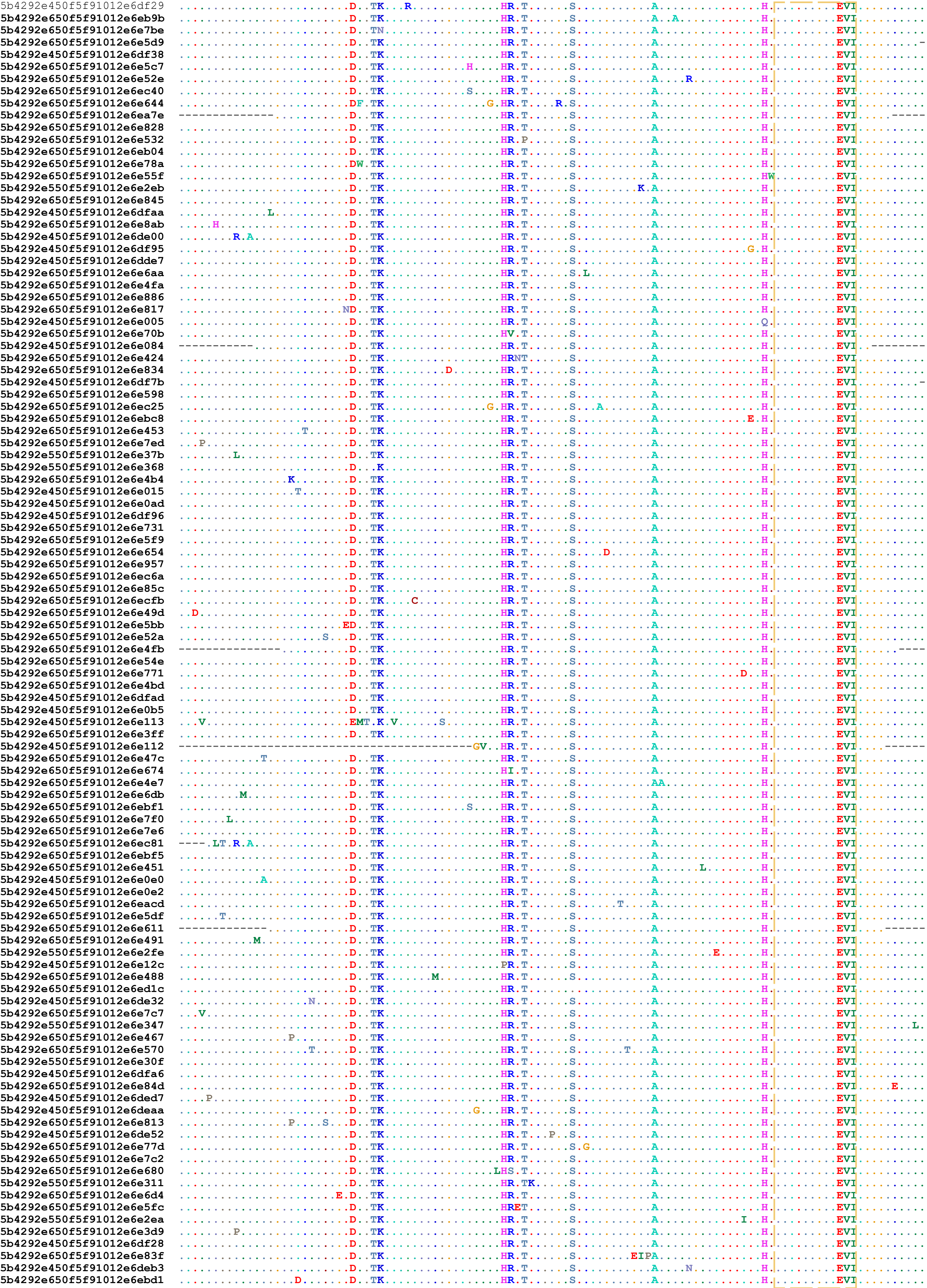
5b4292e450f5f91012e6df29
5b4292e650f5f91012e6eb9b
5b4292e650f5f91012e6e7be
5b4292e650f5f91012e6e5d9
5b4292e450f5f91012e6df38
5b4292e650f5f91012e6e5c7
5b4292e650f5f91012e6e52e
5b4292e650f5f91012e6ec40
5b4292e650f5f91012e6e644
5b4292e650f5f91012e6ea7e
5b4292e650f5f91012e6e828
5b4292e650f5f91012e6e532
5b4292e650f5f91012e6eb04
5b4292e650f5f91012e6e78a
5b4292e650f5f91012e6e55f
5b4292e550f5f91012e6e2eb
5b4292e650f5f91012e6e845
5b4292e450f5f91012e6dfaa
5b4292e650f5f91012e6e8ab
5b4292e450f5f91012e6de00
5b4292e450f5f91012e6df95
5b4292e450f5f91012e6dde7
5b4292e650f5f91012e6e6aa
5b4292e650f5f91012e6e4fa
5b4292e650f5f91012e6e886
5b4292e650f5f91012e6e817
5b4292e450f5f91012e6e005
5b4292e650f5f91012e6e70b
5b4292e450f5f91012e6e084
5b4292e650f5f91012e6e424
5b4292e650f5f91012e6e834
5b4292e450f5f91012e6df7b
5b4292e650f5f91012e6e598
5b4292e650f5f91012e6ec25
5b4292e650f5f91012e6ebc8
5b4292e650f5f91012e6e453
5b4292e650f5f91012e6e7ed
5b4292e550f5f91012e6e37b
5b4292e550f5f91012e6e368
5b4292e650f5f91012e6e4b4
5b4292e450f5f91012e6e015
5b4292e450f5f91012e6e0ad
5b4292e450f5f91012e6df96
5b4292e650f5f91012e6e731
5b4292e650f5f91012e6e5f9
5b4292e650f5f91012e6e654
5b4292e650f5f91012e6e957
5b4292e650f5f91012e6ec6a
5b4292e650f5f91012e6e85c
5b4292e650f5f91012e6ecfb
5b4292e650f5f91012e6e49d
5b4292e650f5f91012e6e5bb
5b4292e650f5f91012e6e52a
5b4292e650f5f91012e6e4fb
5b4292e650f5f91012e6e54e
5b4292e650f5f91012e6e771
5b4292e650f5f91012e6e4bd
5b4292e450f5f91012e6dfad
5b4292e450f5f91012e6e0b5
5b4292e450f5f91012e6e113
5b4292e650f5f91012e6e3ff
5b4292e450f5f91012e6e112
5b4292e650f5f91012e6e47c
5b4292e650f5f91012e6e674
5b4292e650f5f91012e6e4e7
5b4292e650f5f91012e6e6db
5b4292e650f5f91012e6ebf1
5b4292e650f5f91012e6e7f0
5b4292e650f5f91012e6e7e6
5b4292e650f5f91012e6ec81
5b4292e650f5f91012e6ebf5
5b4292e650f5f91012e6e451
5b4292e450f5f91012e6e0e0
5b4292e450f5f91012e6e0e2
5b4292e650f5f91012e6eacd
5b4292e650f5f91012e6e5df
5b4292e650f5f91012e6e611
5b4292e650f5f91012e6e491
5b4292e550f5f91012e6e2fe
5b4292e450f5f91012e6e12c
5b4292e650f5f91012e6e488
5b4292e650f5f91012e6ed1c
5b4292e450f5f91012e6de32
5b4292e650f5f91012e6e7c7
5b4292e550f5f91012e6e347
5b4292e650f5f91012e6e467
5b4292e650f5f91012e6e570
5b4292e550f5f91012e6e30f
5b4292e450f5f91012e6dfa6
5b4292e650f5f91012e6e84d
5b4292e450f5f91012e6ded7
5b4292e450f5f91012e6deaa
5b4292e650f5f91012e6e813
5b4292e450f5f91012e6de52
5b4292e650f5f91012e6e77d
5b4292e650f5f91012e6e7c2
5b4292e650f5f91012e6e680
5b4292e550f5f91012e6e311
5b4292e650f5f91012e6e6d4
5b4292e650f5f91012e6e5fc
5b4292e550f5f91012e6e2ea
5b4292e650f5f91012e6e3d9
5b4292e450f5f91012e6df28
5b4292e650f5f91012e6e83f
5b4292e450f5f91012e6deb3
5b4292e650f5f91012e6ebd1

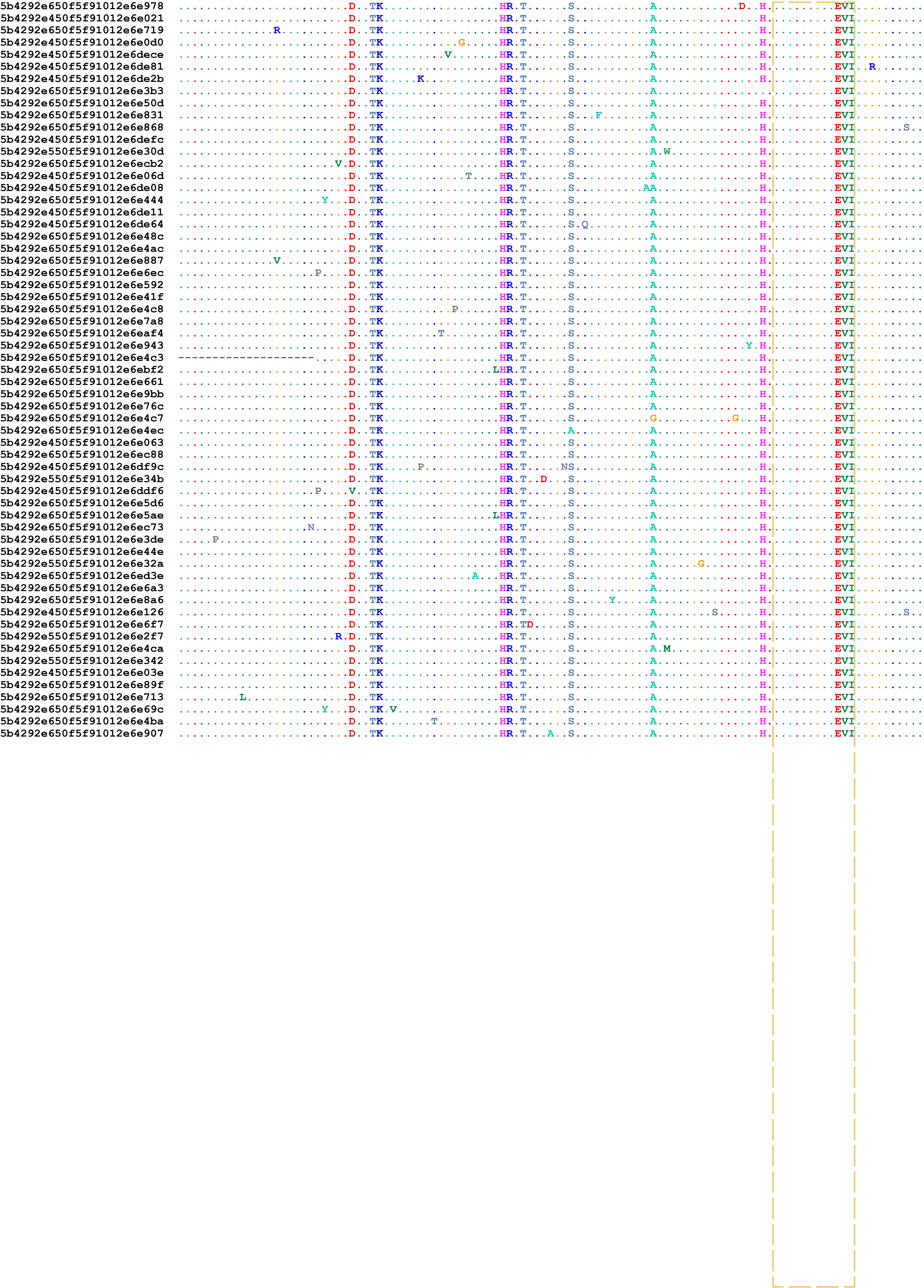
5b4292e650f5f91012e6e978
5b4292e450f5f91012e6e021
5b4292e650f5f91012e6e719
5b4292e450f5f91012e6e0d0
5b4292e450f5f91012e6dece
5b4292e450f5f91012e6de81
5b4292e450f5f91012e6de2b
5b4292e650f5f91012e6e3b3
5b4292e650f5f91012e6e50d
5b4292e650f5f91012e6e831
5b4292e650f5f91012e6e868
5b4292e450f5f91012e6defc
5b4292e550f5f91012e6e30d
5b4292e650f5f91012e6ecb2
5b4292e450f5f91012e6e06d
5b4292e450f5f91012e6de08
5b4292e650f5f91012e6e444
5b4292e450f5f91012e6de11
5b4292e450f5f91012e6de64
5b4292e650f5f91012e6e48c
5b4292e650f5f91012e6e4ac
5b4292e650f5f91012e6e887
5b4292e650f5f91012e6e6ec
5b4292e650f5f91012e6e592
5b4292e650f5f91012e6e41f
5b4292e650f5f91012e6e4c8
5b4292e650f5f91012e6e7a8
5b4292e650f5f91012e6eaf4
5b4292e650f5f91012e6e943
5b4292e650f5f91012e6e4c3
5b4292e650f5f91012e6ebf2
5b4292e650f5f91012e6e661
5b4292e650f5f91012e6e9bb
5b4292e650f5f91012e6e76c
5b4292e650f5f91012e6e4c7
5b4292e650f5f91012e6e4ec
5b4292e450f5f91012e6e063
5b4292e650f5f91012e6ec88
5b4292e450f5f91012e6df9c
5b4292e550f5f91012e6e34b
5b4292e450f5f91012e6ddf6
5b4292e650f5f91012e6e5d6
5b4292e650f5f91012e6e5ae
5b4292e650f5f91012e6ec73
5b4292e650f5f91012e6e3de
5b4292e650f5f91012e6e44e
5b4292e550f5f91012e6e32a
5b4292e650f5f91012e6ed3e
5b4292e650f5f91012e6e6a3
5b4292e650f5f91012e6e8a6
5b4292e450f5f91012e6e126
5b4292e650f5f91012e6e6f7
5b4292e550f5f91012e6e2f7
5b4292e650f5f91012e6e4ca
5b4292e550f5f91012e6e342
5b4292e450f5f91012e6e03e
5b4292e650f5f91012e6e89f
5b4292e650f5f91012e6e713
5b4292e650f5f91012e6e69c
5b4292e650f5f91012e6e4ba
5b4292e650f5f91012e6e907
