# Supplementary material for: Diverse patterns of antibody variable gene repertoire disruption in patients with amyloid light chain (AL) amyloidosis
Source: PLoS One. 2020 Jul 7;15(7):e0235713. doi: 10.1371/journal.pone.0235713 (PMC7340310; doi:10.1371/journal.pone.0235713)
Supplement: S4 Fig — Somatic variants of the dominant clone were aligned to inferred germline genes to create a multiple sequence alignment. (PDF) [file pone.0235713.s006.pdf]

AM2 Timepoint 3

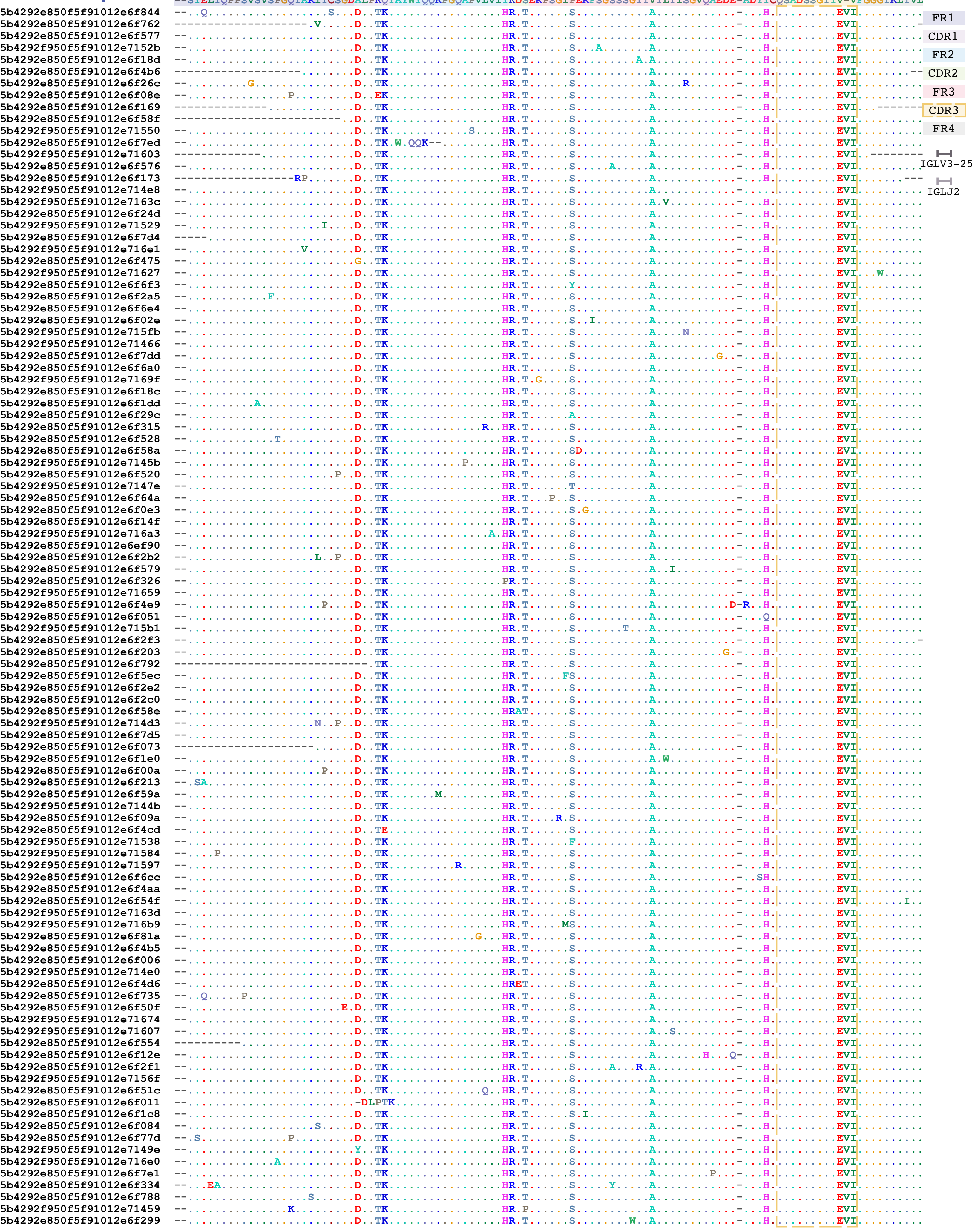

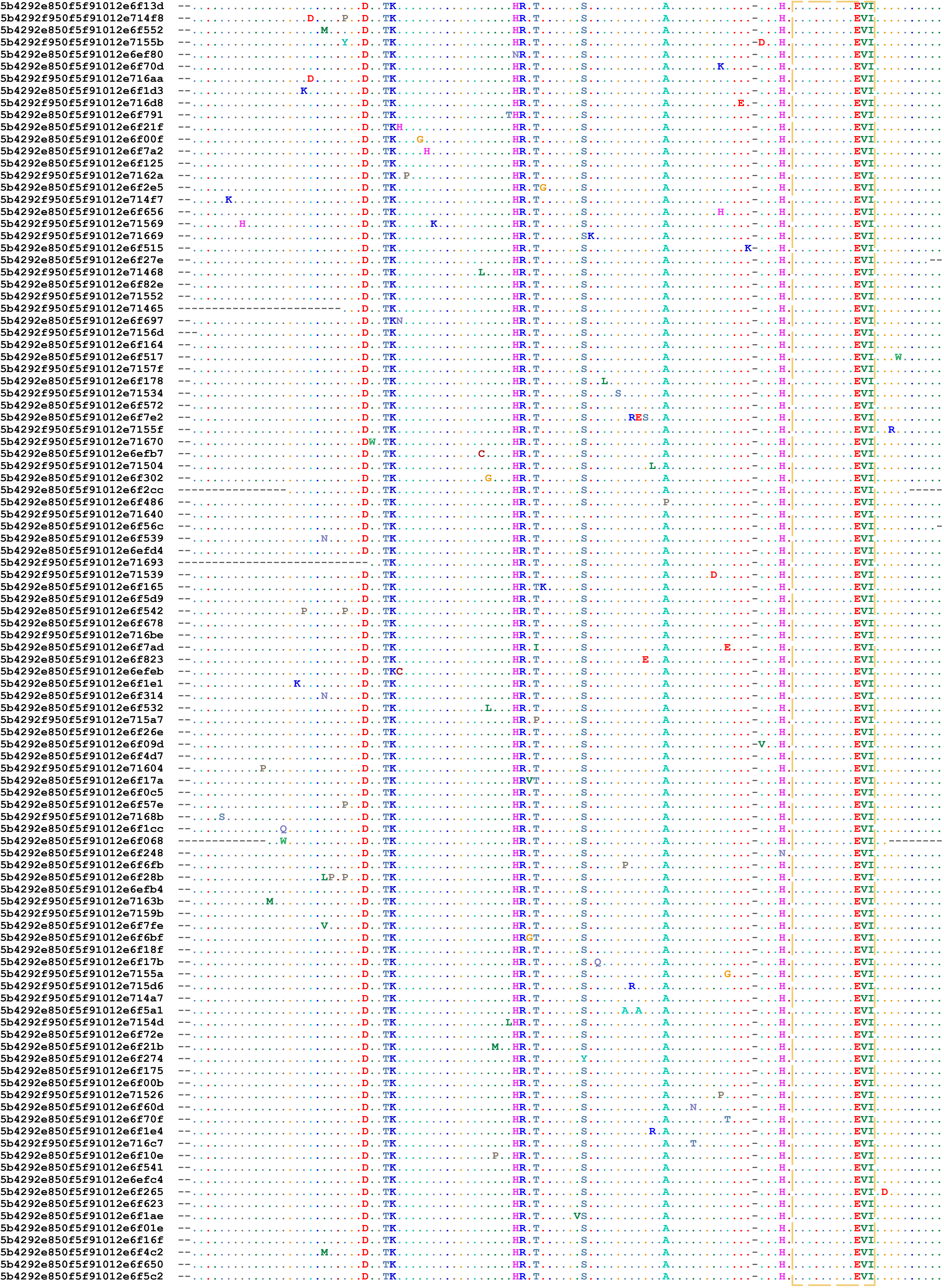
5b4292e850f5f91012e6f13d
5b4292f950f5f91012e714f8
5b4292e850f5f91012e6f552
5b4292f950f5f91012e7155b
5b4292e850f5f91012e6ef80
5b4292e850f5f91012e6f70d
5b4292f950f5f91012e716aa
5b4292e850f5f91012e6f1d3
5b4292f950f5f91012e716d8
5b4292e850f5f91012e6f791
5b4292e850f5f91012e6f21f
5b4292e850f5f91012e6f00f
5b4292e850f5f91012e6f7a2
5b4292e850f5f91012e6f125
5b4292f950f5f91012e7162a
5b4292e850f5f91012e6f2e5
5b4292f950f5f91012e714f7
5b4292e850f5f91012e6f656
5b4292f950f5f91012e71569
5b4292f950f5f91012e71669
5b4292e850f5f91012e6f515
5b4292e850f5f91012e6f27e
5b4292f950f5f91012e71468
5b4292e850f5f91012e6f82e
5b4292f950f5f91012e71552
5b4292f950f5f91012e71465
5b4292e850f5f91012e6f697
5b4292f950f5f91012e7156d
5b4292e850f5f91012e6f164
5b4292e850f5f91012e6f517
5b4292f950f5f91012e7157f
5b4292e850f5f91012e6f178
5b4292f950f5f91012e71534
5b4292e850f5f91012e6f572
5b4292e850f5f91012e6f7e2
5b4292f950f5f91012e7155f
5b4292f950f5f91012e71670
5b4292e850f5f91012e6efb7
5b4292f950f5f91012e71504
5b4292e850f5f91012e6f302
5b4292e850f5f91012e6f2cc
5b4292e850f5f91012e6f486
5b4292f950f5f91012e71640
5b4292e850f5f91012e6f56c
5b4292e850f5f91012e6f539
5b4292e850f5f91012e6efd4
5b4292f950f5f91012e71693
5b4292f950f5f91012e71539
5b4292e850f5f91012e6f165
5b4292e850f5f91012e6f5d9
5b4292e850f5f91012e6f542
5b4292e850f5f91012e6f678
5b4292f950f5f91012e716be
5b4292e850f5f91012e6f7ad
5b4292e850f5f91012e6f823
5b4292e850f5f91012e6efeb
5b4292e850f5f91012e6f1e1
5b4292e850f5f91012e6f314
5b4292e850f5f91012e6f532
5b4292f950f5f91012e715a7
5b4292e850f5f91012e6f26e
5b4292e850f5f91012e6f09d
5b4292e850f5f91012e6f4d7
5b4292f950f5f91012e71604
5b4292e850f5f91012e6f17a
5b4292e850f5f91012e6f0c5
5b4292e850f5f91012e6f57e
5b4292f950f5f91012e7168b
5b4292e850f5f91012e6f1cc
5b4292e850f5f91012e6f068
5b4292e850f5f91012e6f248
5b4292e850f5f91012e6f6fb
5b4292e850f5f91012e6f28b
5b4292e850f5f91012e6efb4
5b4292f950f5f91012e7163b
5b4292f950f5f91012e7159b
5b4292e850f5f91012e6f7fe
5b4292e850f5f91012e6f6bf
5b4292e850f5f91012e6f18f
5b4292e850f5f91012e6f17b
5b4292f950f5f91012e7155a
5b4292f950f5f91012e715d6
5b4292f950f5f91012e714a7
5b4292e850f5f91012e6f5a1
5b4292f950f5f91012e7154d
5b4292e850f5f91012e6f72e
5b4292e850f5f91012e6f21b
5b4292e850f5f91012e6f274
5b4292e850f5f91012e6f175
5b4292e850f5f91012e6f00b
5b4292f950f5f91012e71526
5b4292e850f5f91012e6f60d
5b4292e850f5f91012e6f70f
5b4292e850f5f91012e6f1e4
5b4292f950f5f91012e716c7
5b4292e850f5f91012e6f10e
5b4292e850f5f91012e6f541
5b4292e850f5f91012e6efc4
5b4292e850f5f91012e6f265
5b4292e850f5f91012e6f623
5b4292e850f5f91012e6f1ae
5b4292e850f5f91012e6f01e
5b4292e850f5f91012e6f16f
5b4292e850f5f91012e6f4c2
5b4292e850f5f91012e6f650
5b4292e850f5f91012e6f5c2

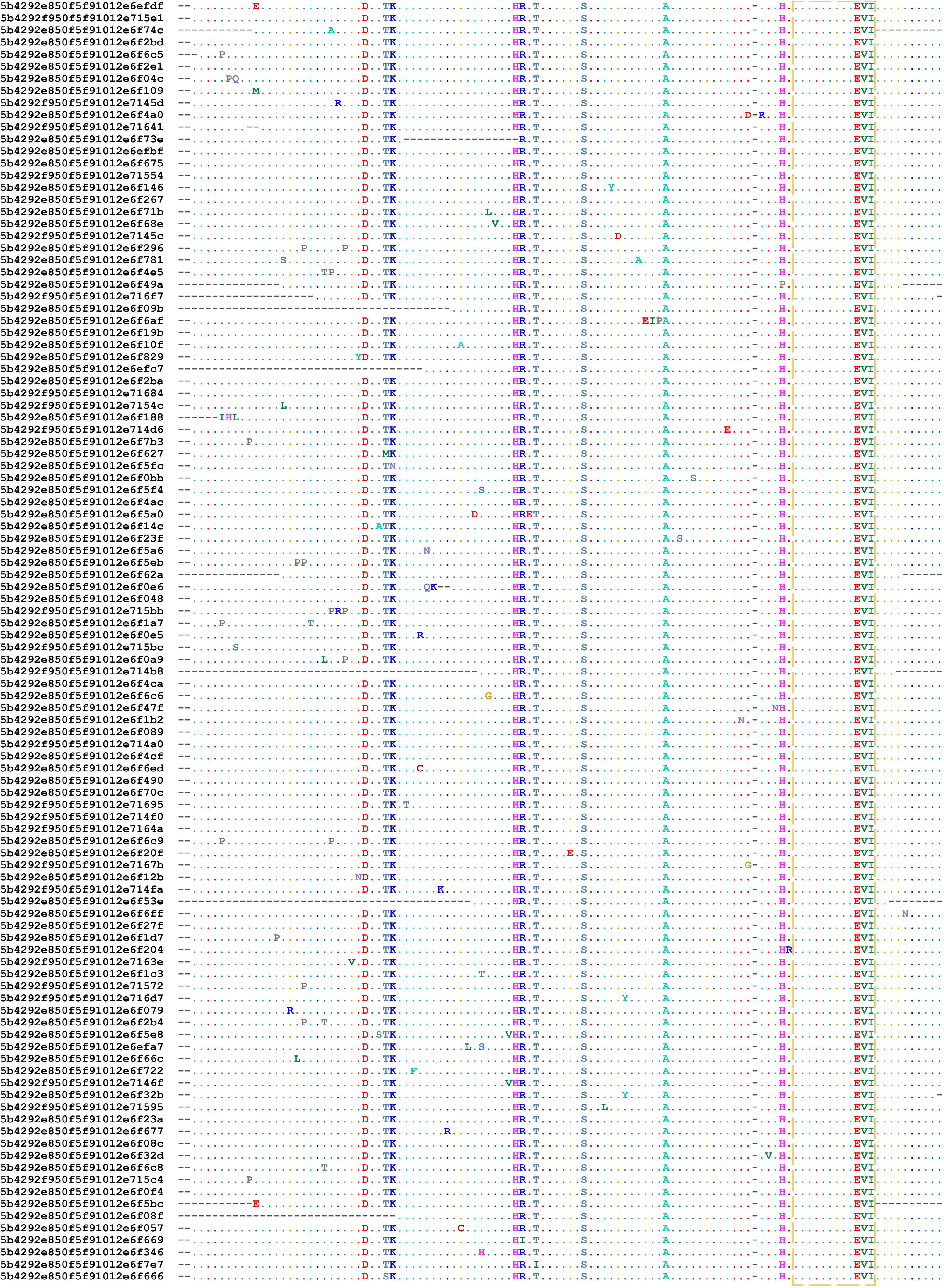
5b4292e850f5f91012e6efdf
5b4292f950f5f91012e715e1
5b4292e850f5f91012e6f74c
5b4292e850f5f91012e6f2bd
5b4292e850f5f91012e6f6c5
5b4292e850f5f91012e6f2e1
5b4292e850f5f91012e6f04c
5b4292e850f5f91012e6f109
5b4292f950f5f91012e7145d
5b4292e850f5f91012e6f4a0
5b4292f950f5f91012e71641
5b4292e850f5f91012e6f73e
5b4292e850f5f91012e6efbf
5b4292e850f5f91012e6f675
5b4292f950f5f91012e71554
5b4292e850f5f91012e6f146
5b4292e850f5f91012e6f267
5b4292e850f5f91012e6f71b
5b4292e850f5f91012e6f68e
5b4292f950f5f91012e7145c
5b4292e850f5f91012e6f296
5b4292e850f5f91012e6f781
5b4292e850f5f91012e6f4e5
5b4292e850f5f91012e6f49a
5b4292f950f5f91012e716f7
5b4292e850f5f91012e6f09b
5b4292e850f5f91012e6f6af
5b4292e850f5f91012e6f19b
5b4292e850f5f91012e6f10f
5b4292e850f5f91012e6f829
5b4292e850f5f91012e6efc7
5b4292e850f5f91012e6f2ba
5b4292f950f5f91012e71684
5b4292f950f5f91012e7154c
5b4292e850f5f91012e6f188
5b4292f950f5f91012e714d6
5b4292e850f5f91012e6f7b3
5b4292e850f5f91012e6f627
5b4292e850f5f91012e6f5fc
5b4292e850f5f91012e6f0bb
5b4292e850f5f91012e6f5f4
5b4292e850f5f91012e6f4ac
5b4292e850f5f91012e6f5a0
5b4292e850f5f91012e6f14c
5b4292e850f5f91012e6f23f
5b4292e850f5f91012e6f5a6
5b4292e850f5f91012e6f5eb
5b4292e850f5f91012e6f62a
5b4292e850f5f91012e6f0e6
5b4292e850f5f91012e6f048
5b4292f950f5f91012e715bb
5b4292e850f5f91012e6f1a7
5b4292e850f5f91012e6f0e5
5b4292f950f5f91012e715bc
5b4292e850f5f91012e6f0a9
5b4292f950f5f91012e714b8
5b4292e850f5f91012e6f4ca
5b4292e850f5f91012e6f6c6
5b4292e850f5f91012e6f47f
5b4292e850f5f91012e6f1b2
5b4292e850f5f91012e6f089
5b4292f950f5f91012e714a0
5b4292e850f5f91012e6f4cf
5b4292e850f5f91012e6f6ed
5b4292e850f5f91012e6f490
5b4292e850f5f91012e6f70c
5b4292f950f5f91012e71695
5b4292f950f5f91012e714f0
5b4292f950f5f91012e7164a
5b4292e850f5f91012e6f6c9
5b4292e850f5f91012e6f20f
5b4292f950f5f91012e7167b
5b4292e850f5f91012e6f12b
5b4292f950f5f91012e714fa
5b4292e850f5f91012e6f53e
5b4292e850f5f91012e6f6ff
5b4292e850f5f91012e6f27f
5b4292e850f5f91012e6f1d7
5b4292e850f5f91012e6f204
5b4292f950f5f91012e7163e
5b4292e850f5f91012e6f1c3
5b4292f950f5f91012e71572
5b4292f950f5f91012e716d7
5b4292e850f5f91012e6f079
5b4292e850f5f91012e6f2b4
5b4292e850f5f91012e6f5e8
5b4292e850f5f91012e6efa7
5b4292e850f5f91012e6f66c
5b4292e850f5f91012e6f722
5b4292f950f5f91012e7146f
5b4292e850f5f91012e6f32b
5b4292f950f5f91012e71595
5b4292e850f5f91012e6f23a
5b4292e850f5f91012e6f677
5b4292e850f5f91012e6f08c
5b4292e850f5f91012e6f32d
5b4292e850f5f91012e6f6c8
5b4292f950f5f91012e715c4
5b4292e850f5f91012e6f0f4
5b4292e850f5f91012e6f5bc
5b4292e850f5f91012e6f08f
5b4292e850f5f91012e6f057
5b4292e850f5f91012e6f669
5b4292e850f5f91012e6f346
5b4292e850f5f91012e6f7e7
5b4292e850f5f91012e6f666

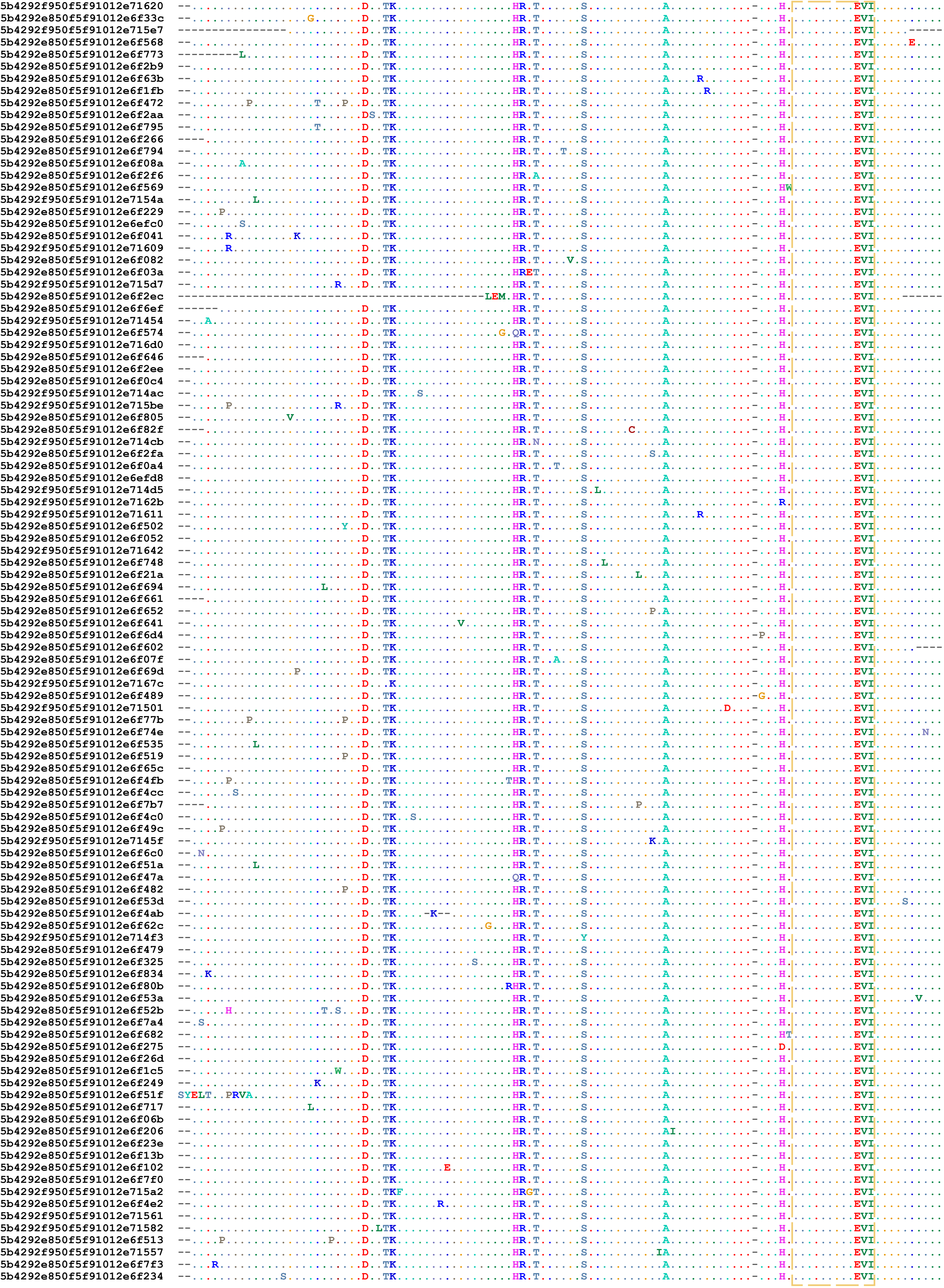
5b4292f950f5f91012e71620
5b4292e850f5f91012e6f33c
5b4292f950f5f91012e715e7
5b4292e850f5f91012e6f568
5b4292e850f5f91012e6f773
5b4292e850f5f91012e6f2b9
5b4292e850f5f91012e6f63b
5b4292e850f5f91012e6f1fb
5b4292e850f5f91012e6f472
5b4292e850f5f91012e6f2aa
5b4292e850f5f91012e6f795
5b4292e850f5f91012e6f266
5b4292e850f5f91012e6f794
5b4292e850f5f91012e6f08a
5b4292e850f5f91012e6f2f6
5b4292e850f5f91012e6f569
5b4292f950f5f91012e7154a
5b4292e850f5f91012e6f229
5b4292e850f5f91012e6efc0
5b4292e850f5f91012e6f041
5b4292f950f5f91012e71609
5b4292e850f5f91012e6f082
5b4292e850f5f91012e6f03a
5b4292f950f5f91012e715d7
5b4292e850f5f91012e6f2ec
5b4292e850f5f91012e6f6ef
5b4292f950f5f91012e71454
5b4292e850f5f91012e6f574
5b4292f950f5f91012e716d0
5b4292e850f5f91012e6f646
5b4292e850f5f91012e6f2ee
5b4292e850f5f91012e6f0c4
5b4292f950f5f91012e714ac
5b4292f950f5f91012e715be
5b4292e850f5f91012e6f805
5b4292e850f5f91012e6f82f
5b4292f950f5f91012e714cb
5b4292e850f5f91012e6f2fa
5b4292e850f5f91012e6f0a4
5b4292e850f5f91012e6efd8
5b4292f950f5f91012e714d5
5b4292f950f5f91012e7162b
5b4292f950f5f91012e71611
5b4292e850f5f91012e6f502
5b4292e850f5f91012e6f052
5b4292f950f5f91012e71642
5b4292e850f5f91012e6f748
5b4292e850f5f91012e6f21a
5b4292e850f5f91012e6f694
5b4292e850f5f91012e6f661
5b4292e850f5f91012e6f652
5b4292e850f5f91012e6f641
5b4292e850f5f91012e6f6d4
5b4292e850f5f91012e6f602
5b4292e850f5f91012e6f07f
5b4292e850f5f91012e6f69d
5b4292f950f5f91012e7167c
5b4292e850f5f91012e6f489
5b4292f950f5f91012e71501
5b4292e850f5f91012e6f77b
5b4292e850f5f91012e6f74e
5b4292e850f5f91012e6f535
5b4292e850f5f91012e6f519
5b4292e850f5f91012e6f65c
5b4292e850f5f91012e6f4fb
5b4292e850f5f91012e6f4cc
5b4292e850f5f91012e6f7b7
5b4292e850f5f91012e6f4c0
5b4292e850f5f91012e6f49c
5b4292f950f5f91012e7145f
5b4292e850f5f91012e6f6c0
5b4292e850f5f91012e6f51a
5b4292e850f5f91012e6f47a
5b4292e850f5f91012e6f482
5b4292e850f5f91012e6f53d
5b4292e850f5f91012e6f4ab
5b4292e850f5f91012e6f62c
5b4292f950f5f91012e714f3
5b4292e850f5f91012e6f479
5b4292e850f5f91012e6f325
5b4292e850f5f91012e6f834
5b4292e850f5f91012e6f80b
5b4292e850f5f91012e6f53a
5b4292e850f5f91012e6f52b
5b4292e850f5f91012e6f7a4
5b4292e850f5f91012e6f682
5b4292e850f5f91012e6f275
5b4292e850f5f91012e6f26d
5b4292e850f5f91012e6f1c5
5b4292e850f5f91012e6f249
5b4292e850f5f91012e6f51f
5b4292e850f5f91012e6f717
5b4292e850f5f91012e6f06b
5b4292e850f5f91012e6f206
5b4292e850f5f91012e6f23e
5b4292e850f5f91012e6f13b
5b4292e850f5f91012e6f102
5b4292e850f5f91012e6f7f0
5b4292f950f5f91012e715a2
5b4292e850f5f91012e6f4e2
5b4292f950f5f91012e71561
5b4292f950f5f91012e71582
5b4292e850f5f91012e6f513
5b4292f950f5f91012e71557
5b4292e850f5f91012e6f7f3
5b4292e850f5f91012e6f234

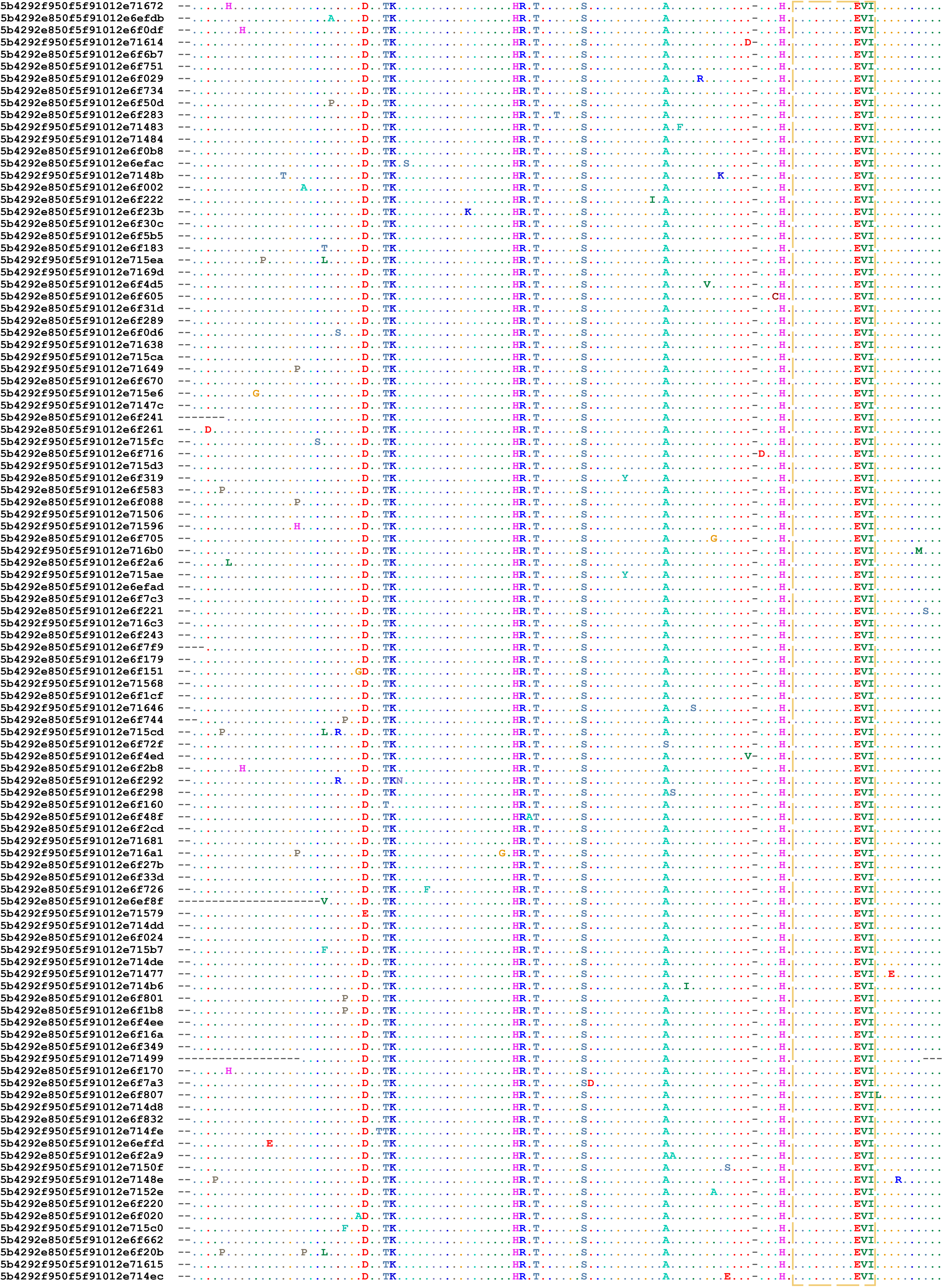
5b4292f950f5f91012e71672
5b4292e850f5f91012e6efdb
5b4292e850f5f91012e6f0df
5b4292f950f5f91012e71614
5b4292e850f5f91012e6f6b7
5b4292e850f5f91012e6f751
5b4292e850f5f91012e6f029
5b4292e850f5f91012e6f734
5b4292e850f5f91012e6f50d
5b4292e850f5f91012e6f283
5b4292f950f5f91012e71483
5b4292f950f5f91012e71484
5b4292e850f5f91012e6f0b8
5b4292e850f5f91012e6efac
5b4292f950f5f91012e7148b
5b4292e850f5f91012e6f002
5b4292e850f5f91012e6f222
5b4292e850f5f91012e6f23b
5b4292e850f5f91012e6f30c
5b4292e850f5f91012e6f5b5
5b4292e850f5f91012e6f183
5b4292f950f5f91012e715ea
5b4292f950f5f91012e7169d
5b4292e850f5f91012e6f4d5
5b4292e850f5f91012e6f605
5b4292e850f5f91012e6f31d
5b4292e850f5f91012e6f289
5b4292e850f5f91012e6f0d6
5b4292f950f5f91012e71638
5b4292f950f5f91012e715ca
5b4292f950f5f91012e71649
5b4292e850f5f91012e6f670
5b4292f950f5f91012e715e6
5b4292f950f5f91012e7147c
5b4292e850f5f91012e6f241
5b4292e850f5f91012e6f261
5b4292f950f5f91012e715fc
5b4292e850f5f91012e6f716
5b4292f950f5f91012e715d3
5b4292e850f5f91012e6f319
5b4292e850f5f91012e6f583
5b4292e850f5f91012e6f088
5b4292f950f5f91012e71506
5b4292f950f5f91012e71596
5b4292e850f5f91012e6f705
5b4292f950f5f91012e716b0
5b4292e850f5f91012e6f2a6
5b4292f950f5f91012e715ae
5b4292e850f5f91012e6efad
5b4292e850f5f91012e6f7c3
5b4292e850f5f91012e6f221
5b4292f950f5f91012e716c3
5b4292e850f5f91012e6f243
5b4292e850f5f91012e6f7f9
5b4292e850f5f91012e6f179
5b4292e850f5f91012e6f151
5b4292f950f5f91012e71568
5b4292e850f5f91012e6f1cf
5b4292f950f5f91012e71646
5b4292e850f5f91012e6f744
5b4292f950f5f91012e715cd
5b4292e850f5f91012e6f72f
5b4292e850f5f91012e6f4ed
5b4292e850f5f91012e6f2b8
5b4292e850f5f91012e6f292
5b4292e850f5f91012e6f298
5b4292e850f5f91012e6f160
5b4292e850f5f91012e6f48f
5b4292e850f5f91012e6f2cd
5b4292f950f5f91012e71681
5b4292f950f5f91012e716a1
5b4292e850f5f91012e6f27b
5b4292e850f5f91012e6f33d
5b4292e850f5f91012e6f726
5b4292e850f5f91012e6ef8f
5b4292f950f5f91012e71579
5b4292f950f5f91012e714dd
5b4292e850f5f91012e6f024
5b4292f950f5f91012e715b7
5b4292f950f5f91012e714de
5b4292f950f5f91012e71477
5b4292f950f5f91012e714b6
5b4292e850f5f91012e6f801
5b4292e850f5f91012e6f1b8
5b4292e850f5f91012e6f4ee
5b4292e850f5f91012e6f16a
5b4292e850f5f91012e6f349
5b4292f950f5f91012e71499
5b4292e850f5f91012e6f170
5b4292e850f5f91012e6f7a3
5b4292e850f5f91012e6f807
5b4292f950f5f91012e714d8
5b4292e850f5f91012e6f832
5b4292f950f5f91012e714fe
5b4292e850f5f91012e6effd
5b4292e850f5f91012e6f2a9
5b4292f950f5f91012e7150f
5b4292f950f5f91012e7148e
5b4292f950f5f91012e7152e
5b4292e850f5f91012e6f220
5b4292e850f5f91012e6f020
5b4292f950f5f91012e715c0
5b4292e850f5f91012e6f662
5b4292e850f5f91012e6f20b
5b4292f950f5f91012e71615
5b4292f950f5f91012e714ec

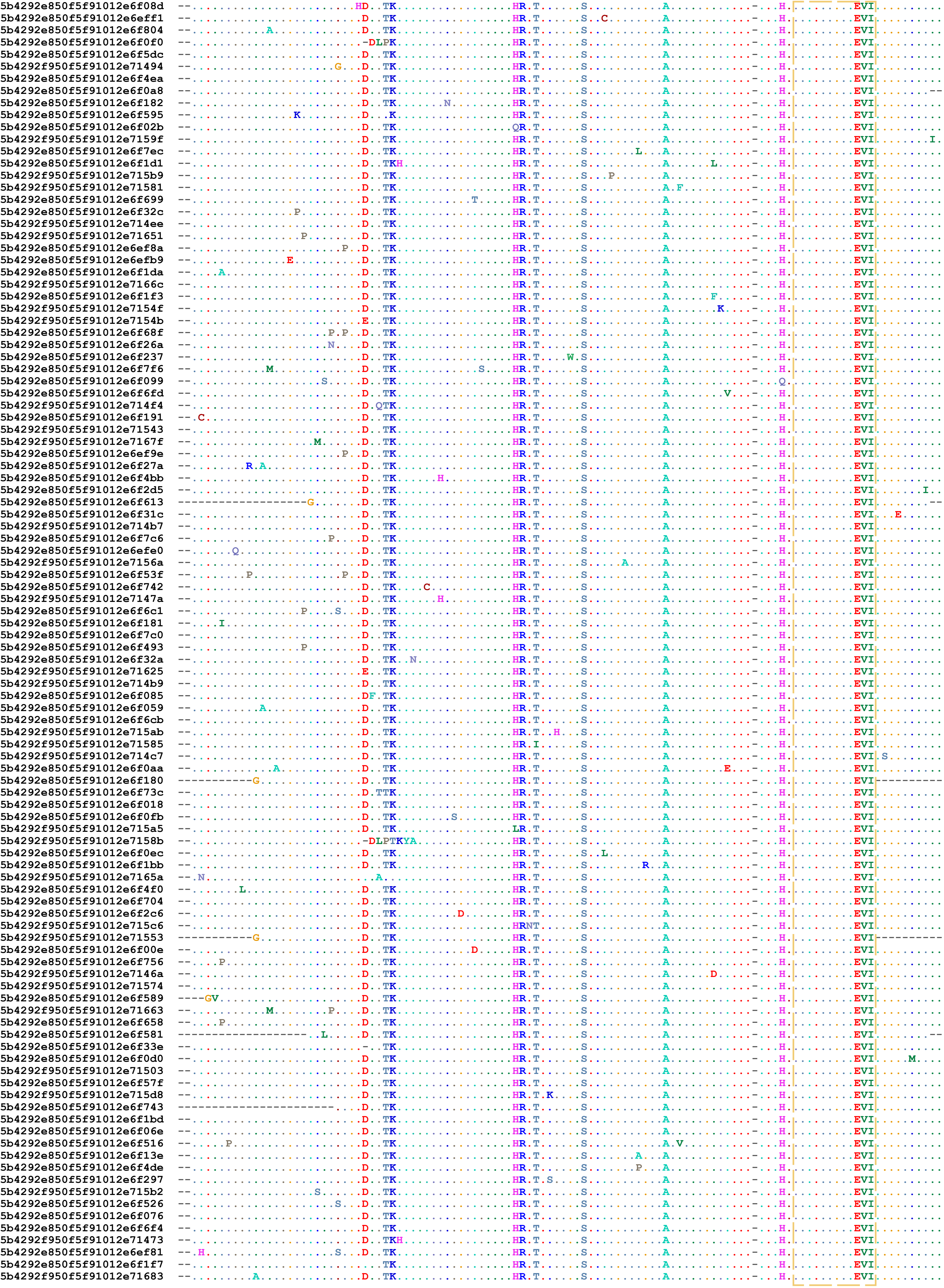
5b4292e850f5f91012e6f08d
5b4292e850f5f91012e6eff1
5b4292e850f5f91012e6f804
5b4292e850f5f91012e6f0f0
5b4292e850f5f91012e6f5dc
5b4292f950f5f91012e71494
5b4292e850f5f91012e6f4ea
5b4292e850f5f91012e6f0a8
5b4292e850f5f91012e6f182
5b4292e850f5f91012e6f595
5b4292e850f5f91012e6f02b
5b4292f950f5f91012e7159f
5b4292e850f5f91012e6f7ec
5b4292e850f5f91012e6f1d1
5b4292f950f5f91012e715b9
5b4292f950f5f91012e71581
5b4292e850f5f91012e6f699
5b4292e850f5f91012e6f32c
5b4292f950f5f91012e714ee
5b4292f950f5f91012e71651
5b4292e850f5f91012e6ef8a
5b4292e850f5f91012e6efb9
5b4292e850f5f91012e6f1da
5b4292f950f5f91012e7166c
5b4292e850f5f91012e6f1f3
5b4292f950f5f91012e7154f
5b4292f950f5f91012e7154b
5b4292e850f5f91012e6f68f
5b4292e850f5f91012e6f26a
5b4292e850f5f91012e6f237
5b4292e850f5f91012e6f7f6
5b4292e850f5f91012e6f099
5b4292e850f5f91012e6f6fd
5b4292f950f5f91012e714f4
5b4292e850f5f91012e6f191
5b4292f950f5f91012e71543
5b4292f950f5f91012e7167f
5b4292e850f5f91012e6ef9e
5b4292e850f5f91012e6f27a
5b4292e850f5f91012e6f4bb
5b4292e850f5f91012e6f2d5
5b4292e850f5f91012e6f613
5b4292e850f5f91012e6f31c
5b4292f950f5f91012e714b7
5b4292e850f5f91012e6f7c6
5b4292e850f5f91012e6efe0
5b4292f950f5f91012e7156a
5b4292e850f5f91012e6f53f
5b4292e850f5f91012e6f742
5b4292f950f5f91012e7147a
5b4292e850f5f91012e6f6c1
5b4292e850f5f91012e6f181
5b4292e850f5f91012e6f7c0
5b4292e850f5f91012e6f493
5b4292e850f5f91012e6f32a
5b4292f950f5f91012e71625
5b4292f950f5f91012e714b9
5b4292e850f5f91012e6f085
5b4292e850f5f91012e6f059
5b4292e850f5f91012e6f6cb
5b4292f950f5f91012e715ab
5b4292f950f5f91012e71585
5b4292f950f5f91012e714c7
5b4292e850f5f91012e6f0aa
5b4292e850f5f91012e6f180
5b4292e850f5f91012e6f73c
5b4292e850f5f91012e6f018
5b4292e850f5f91012e6f0fb
5b4292f950f5f91012e715a5
5b4292f950f5f91012e7158b
5b4292e850f5f91012e6f0ec
5b4292e850f5f91012e6f1bb
5b4292f950f5f91012e7165a
5b4292e850f5f91012e6f4f0
5b4292e850f5f91012e6f704
5b4292e850f5f91012e6f2c6
5b4292f950f5f91012e715c6
5b4292f950f5f91012e71553
5b4292e850f5f91012e6f00e
5b4292e850f5f91012e6f756
5b4292f950f5f91012e7146a
5b4292f950f5f91012e71574
5b4292e850f5f91012e6f589
5b4292f950f5f91012e71663
5b4292e850f5f91012e6f658
5b4292e850f5f91012e6f581
5b4292e850f5f91012e6f33e
5b4292e850f5f91012e6f0d0
5b4292f950f5f91012e71503
5b4292e850f5f91012e6f57f
5b4292f950f5f91012e715d8
5b4292e850f5f91012e6f743
5b4292e850f5f91012e6f1bd
5b4292e850f5f91012e6f06e
5b4292e850f5f91012e6f516
5b4292e850f5f91012e6f13e
5b4292e850f5f91012e6f4de
5b4292e850f5f91012e6f297
5b4292f950f5f91012e715b2
5b4292e850f5f91012e6f526
5b4292e850f5f91012e6f076
5b4292e850f5f91012e6f6f4
5b4292f950f5f91012e71473
5b4292e850f5f91012e6ef81
5b4292e850f5f91012e6f1f7
5b4292f950f5f91012e71683

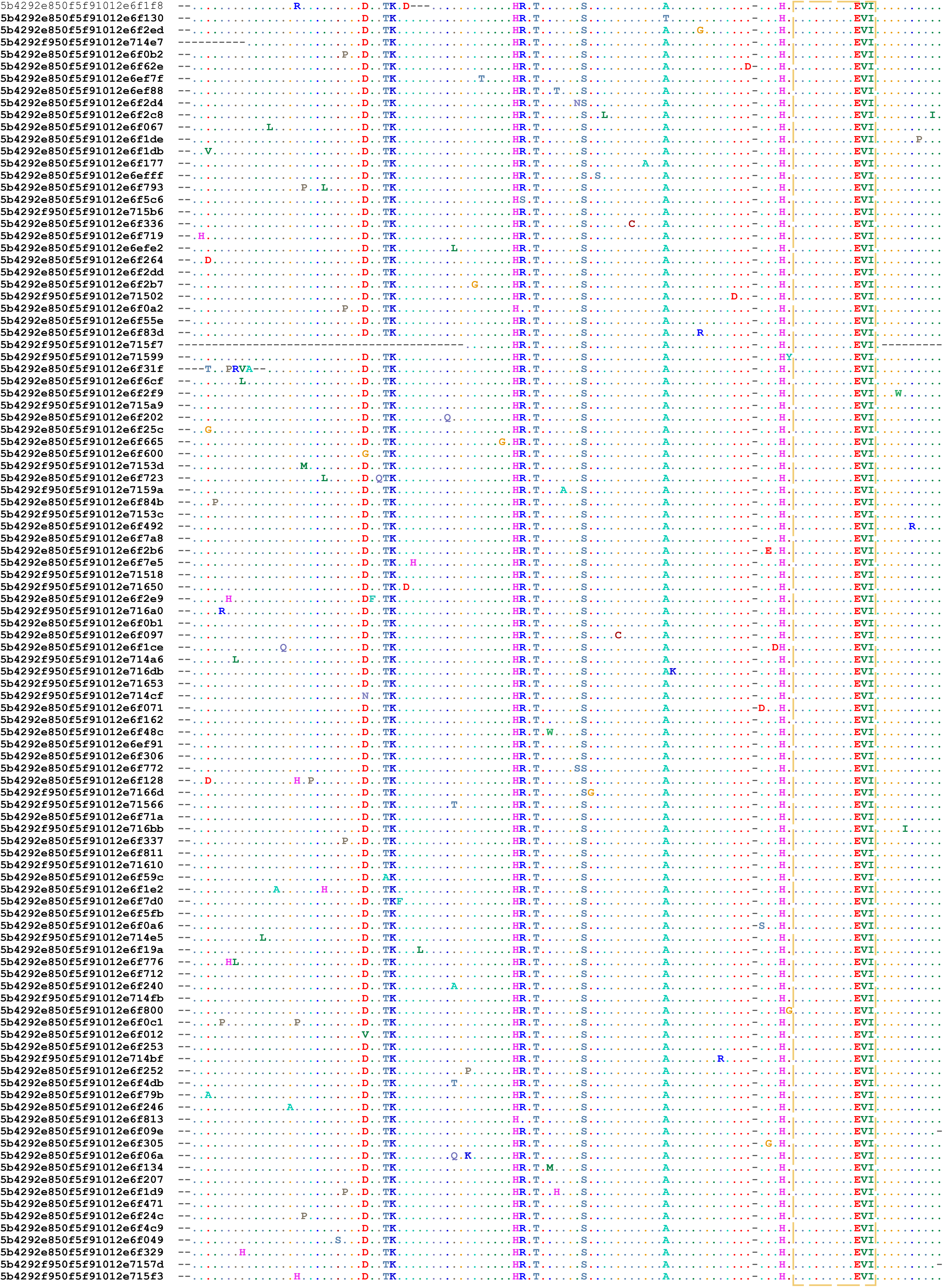

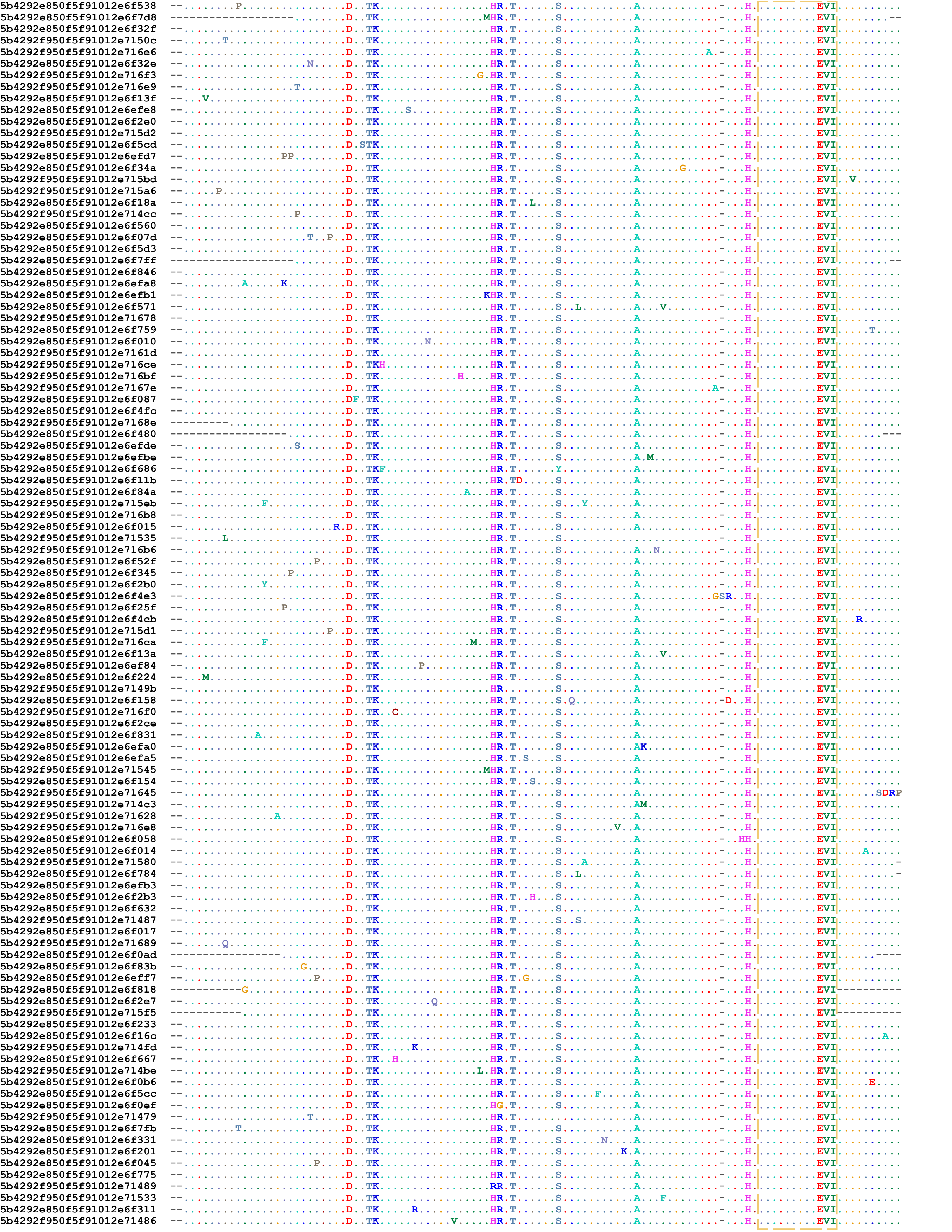
5b4292e850f5f91012e6f538
5b4292e850f5f91012e6f7d8
5b4292e850f5f91012e6f32f
5b4292f950f5f91012e7150c
5b4292f950f5f91012e716e6
5b4292e850f5f91012e6f32e
5b4292f950f5f91012e716f3
5b4292f950f5f91012e716e9
5b4292e850f5f91012e6f13f
5b4292e850f5f91012e6efe8
5b4292e850f5f91012e6f2e0
5b4292f950f5f91012e715d2
5b4292e850f5f91012e6f5cd
5b4292e850f5f91012e6efd7
5b4292e850f5f91012e6f34a
5b4292f950f5f91012e715bd
5b4292f950f5f91012e715a6
5b4292e850f5f91012e6f18a
5b4292f950f5f91012e714cc
5b4292e850f5f91012e6f560
5b4292e850f5f91012e6f07d
5b4292e850f5f91012e6f5d3
5b4292e850f5f91012e6f7ff
5b4292e850f5f91012e6f846
5b4292e850f5f91012e6efa8
5b4292e850f5f91012e6efb1
5b4292e850f5f91012e6f571
5b4292f950f5f91012e71678
5b4292e850f5f91012e6f759
5b4292e850f5f91012e6f010
5b4292f950f5f91012e7161d
5b4292f950f5f91012e716ce
5b4292f950f5f91012e716bf
5b4292f950f5f91012e7167e
5b4292e850f5f91012e6f087
5b4292e850f5f91012e6f4fc
5b4292f950f5f91012e7168e
5b4292e850f5f91012e6f480
5b4292e850f5f91012e6efde
5b4292e850f5f91012e6efbe
5b4292e850f5f91012e6f686
5b4292e850f5f91012e6f11b
5b4292e850f5f91012e6f84a
5b4292f950f5f91012e715eb
5b4292f950f5f91012e716b8
5b4292e850f5f91012e6f015
5b4292f950f5f91012e71535
5b4292f950f5f91012e716b6
5b4292e850f5f91012e6f52f
5b4292e850f5f91012e6f345
5b4292e850f5f91012e6f2b0
5b4292e850f5f91012e6f4e3
5b4292e850f5f91012e6f25f
5b4292e850f5f91012e6f4cb
5b4292f950f5f91012e715d1
5b4292f950f5f91012e716ca
5b4292e850f5f91012e6f13a
5b4292e850f5f91012e6ef84
5b4292e850f5f91012e6f224
5b4292f950f5f91012e7149b
5b4292e850f5f91012e6f158
5b4292f950f5f91012e716f0
5b4292e850f5f91012e6f2ce
5b4292e850f5f91012e6f831
5b4292e850f5f91012e6efa0
5b4292e850f5f91012e6efa5
5b4292f950f5f91012e71545
5b4292e850f5f91012e6f154
5b4292f950f5f91012e71645
5b4292f950f5f91012e714c3
5b4292f950f5f91012e71628
5b4292f950f5f91012e716e8
5b4292e850f5f91012e6f058
5b4292e850f5f91012e6f014
5b4292f950f5f91012e71580
5b4292e850f5f91012e6f784
5b4292e850f5f91012e6efb3
5b4292e850f5f91012e6f2b3
5b4292e850f5f91012e6f632
5b4292f950f5f91012e71487
5b4292e850f5f91012e6f017
5b4292f950f5f91012e71689
5b4292e850f5f91012e6f0ad
5b4292e850f5f91012e6f83b
5b4292e850f5f91012e6eff7
5b4292e850f5f91012e6f818
5b4292e850f5f91012e6f2e7
5b4292f950f5f91012e715f5
5b4292e850f5f91012e6f233
5b4292e850f5f91012e6f16c
5b4292f950f5f91012e714fd
5b4292e850f5f91012e6f667
5b4292f950f5f91012e714be
5b4292e850f5f91012e6f0b6
5b4292e850f5f91012e6f5cc
5b4292e850f5f91012e6f0ef
5b4292f950f5f91012e71479
5b4292e850f5f91012e6f7fb
5b4292e850f5f91012e6f331
5b4292e850f5f91012e6f201
5b4292e850f5f91012e6f045
5b4292e850f5f91012e6f775
5b4292f950f5f91012e71489
5b4292f950f5f91012e71533
5b4292e850f5f91012e6f311
5b4292f950f5f91012e71486

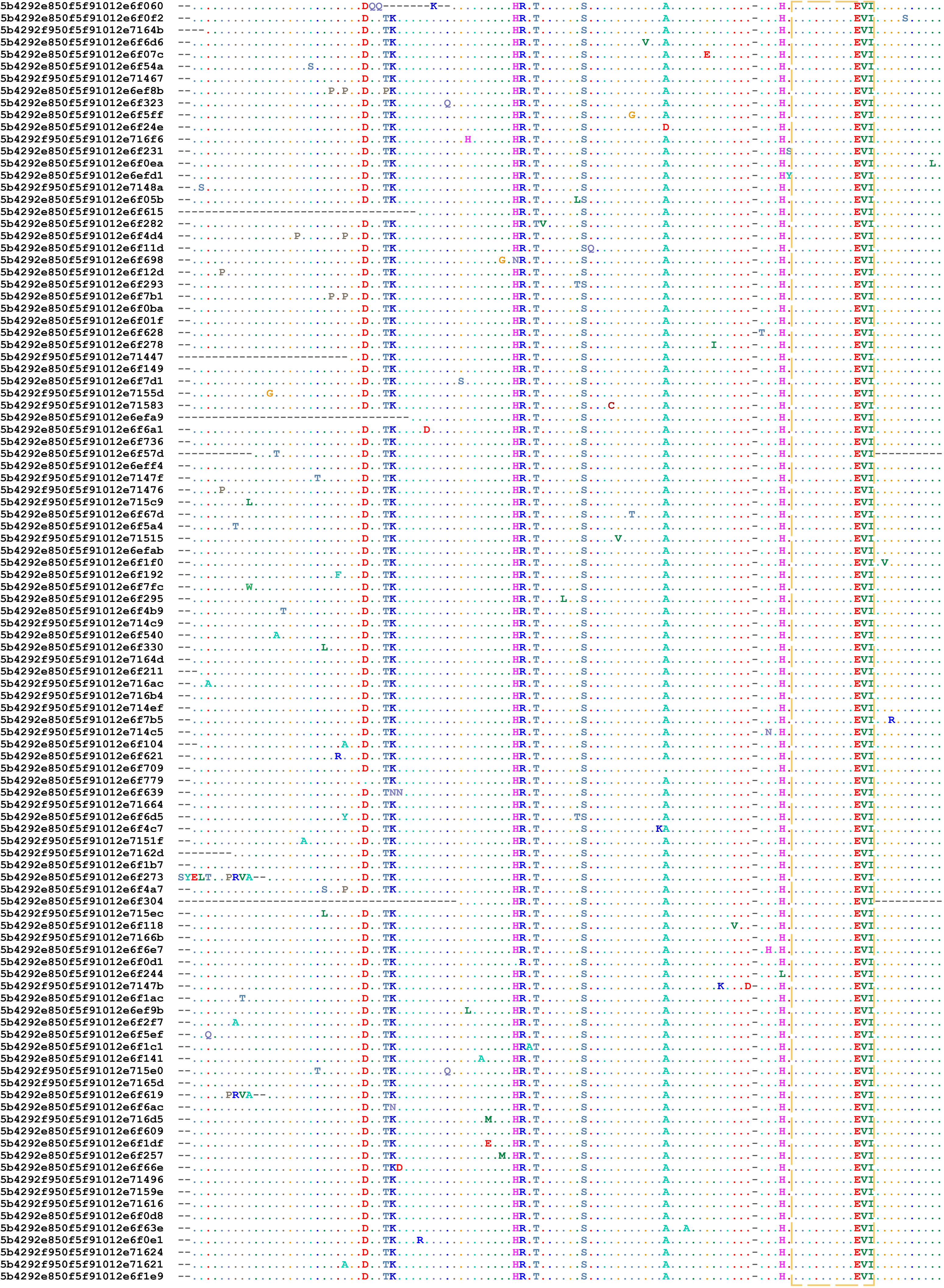
5b4292e850f5f91012e6f060
5b4292e850f5f91012e6f0f2
5b4292f950f5f91012e7164b
5b4292e850f5f91012e6f6d6
5b4292e850f5f91012e6f07c
5b4292e850f5f91012e6f54a
5b4292f950f5f91012e71467
5b4292e850f5f91012e6ef8b
5b4292e850f5f91012e6f323
5b4292e850f5f91012e6f5ff
5b4292e850f5f91012e6f24e
5b4292f950f5f91012e716f6
5b4292e850f5f91012e6f231
5b4292e850f5f91012e6f0ea
5b4292e850f5f91012e6efd1
5b4292f950f5f91012e7148a
5b4292e850f5f91012e6f05b
5b4292e850f5f91012e6f615
5b4292e850f5f91012e6f282
5b4292e850f5f91012e6f4d4
5b4292e850f5f91012e6f11d
5b4292e850f5f91012e6f698
5b4292e850f5f91012e6f12d
5b4292e850f5f91012e6f293
5b4292e850f5f91012e6f7b1
5b4292e850f5f91012e6f0ba
5b4292e850f5f91012e6f01f
5b4292e850f5f91012e6f628
5b4292e850f5f91012e6f278
5b4292f950f5f91012e71447
5b4292e850f5f91012e6f149
5b4292e850f5f91012e6f7d1
5b4292f950f5f91012e7155d
5b4292f950f5f91012e71583
5b4292e850f5f91012e6efa9
5b4292e850f5f91012e6f6a1
5b4292e850f5f91012e6f736
5b4292e850f5f91012e6f57d
5b4292e850f5f91012e6eff4
5b4292f950f5f91012e7147f
5b4292f950f5f91012e71476
5b4292f950f5f91012e715c9
5b4292e850f5f91012e6f67d
5b4292e850f5f91012e6f5a4
5b4292f950f5f91012e71515
5b4292e850f5f91012e6efab
5b4292e850f5f91012e6f1f0
5b4292e850f5f91012e6f192
5b4292e850f5f91012e6f7fc
5b4292e850f5f91012e6f295
5b4292e850f5f91012e6f4b9
5b4292f950f5f91012e714c9
5b4292e850f5f91012e6f540
5b4292e850f5f91012e6f330
5b4292f950f5f91012e7164d
5b4292e850f5f91012e6f211
5b4292f950f5f91012e716ac
5b4292f950f5f91012e716b4
5b4292f950f5f91012e714ef
5b4292e850f5f91012e6f7b5
5b4292f950f5f91012e714c5
5b4292e850f5f91012e6f104
5b4292e850f5f91012e6f621
5b4292e850f5f91012e6f709
5b4292e850f5f91012e6f779
5b4292e850f5f91012e6f639
5b4292f950f5f91012e71664
5b4292e850f5f91012e6f6d5
5b4292e850f5f91012e6f4c7
5b4292f950f5f91012e7151f
5b4292f950f5f91012e7162d
5b4292e850f5f91012e6f1b7
5b4292e850f5f91012e6f273
5b4292e850f5f91012e6f4a7
5b4292e850f5f91012e6f304
5b4292f950f5f91012e715ec
5b4292e850f5f91012e6f118
5b4292f950f5f91012e7166b
5b4292e850f5f91012e6f6e7
5b4292e850f5f91012e6f0d1
5b4292e850f5f91012e6f244
5b4292f950f5f91012e7147b
5b4292e850f5f91012e6f1ac
5b4292e850f5f91012e6ef9b
5b4292e850f5f91012e6f2f7
5b4292e850f5f91012e6f5ef
5b4292e850f5f91012e6f1c1
5b4292e850f5f91012e6f141
5b4292f950f5f91012e715e0
5b4292f950f5f91012e7165d
5b4292e850f5f91012e6f619
5b4292e850f5f91012e6f6ac
5b4292f950f5f91012e716d5
5b4292e850f5f91012e6f609
5b4292e850f5f91012e6f1df
5b4292e850f5f91012e6f257
5b4292e850f5f91012e6f66e
5b4292f950f5f91012e71496
5b4292f950f5f91012e7159e
5b4292f950f5f91012e71616
5b4292e850f5f91012e6f0d8
5b4292e850f5f91012e6f63e
5b4292e850f5f91012e6f0e1
5b4292f950f5f91012e71624
5b4292f950f5f91012e71621
5b4292e850f5f91012e6f1e9

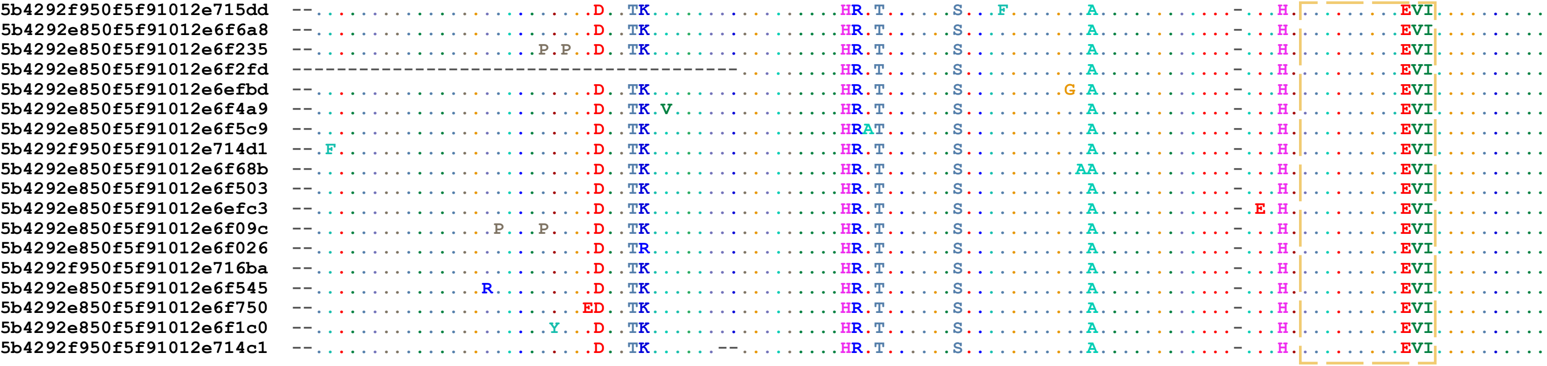
5b4292f950f5f91012e715dd
5b4292e850f5f91012e6f6a8
5b4292e850f5f91012e6f235
5b4292e850f5f91012e6f2fd
5b4292e850f5f91012e6efbd
5b4292e850f5f91012e6f4a9
5b4292e850f5f91012e6f5c9
5b4292f950f5f91012e714d1
5b4292e850f5f91012e6f68b
5b4292e850f5f91012e6f503
5b4292e850f5f91012e6efc3
5b4292e850f5f91012e6f09c
5b4292e850f5f91012e6f026
5b4292f950f5f91012e716ba
5b4292e850f5f91012e6f545
5b4292e850f5f91012e6f750
5b4292e850f5f91012e6f1c0
5b4292f950f5f91012e714c1
