# Supplementary material for: Diverse patterns of antibody variable gene repertoire disruption in patients with amyloid light chain (AL) amyloidosis
Source: PLoS One. 2020 Jul 7;15(7):e0235713. doi: 10.1371/journal.pone.0235713 (PMC7340310; doi:10.1371/journal.pone.0235713)
Supplement: S5 Fig — Somatic variants of the dominant clone were aligned to inferred germline genes to create a multiple sequence alignment. (PDF) [file pone.0235713.s007.pdf]

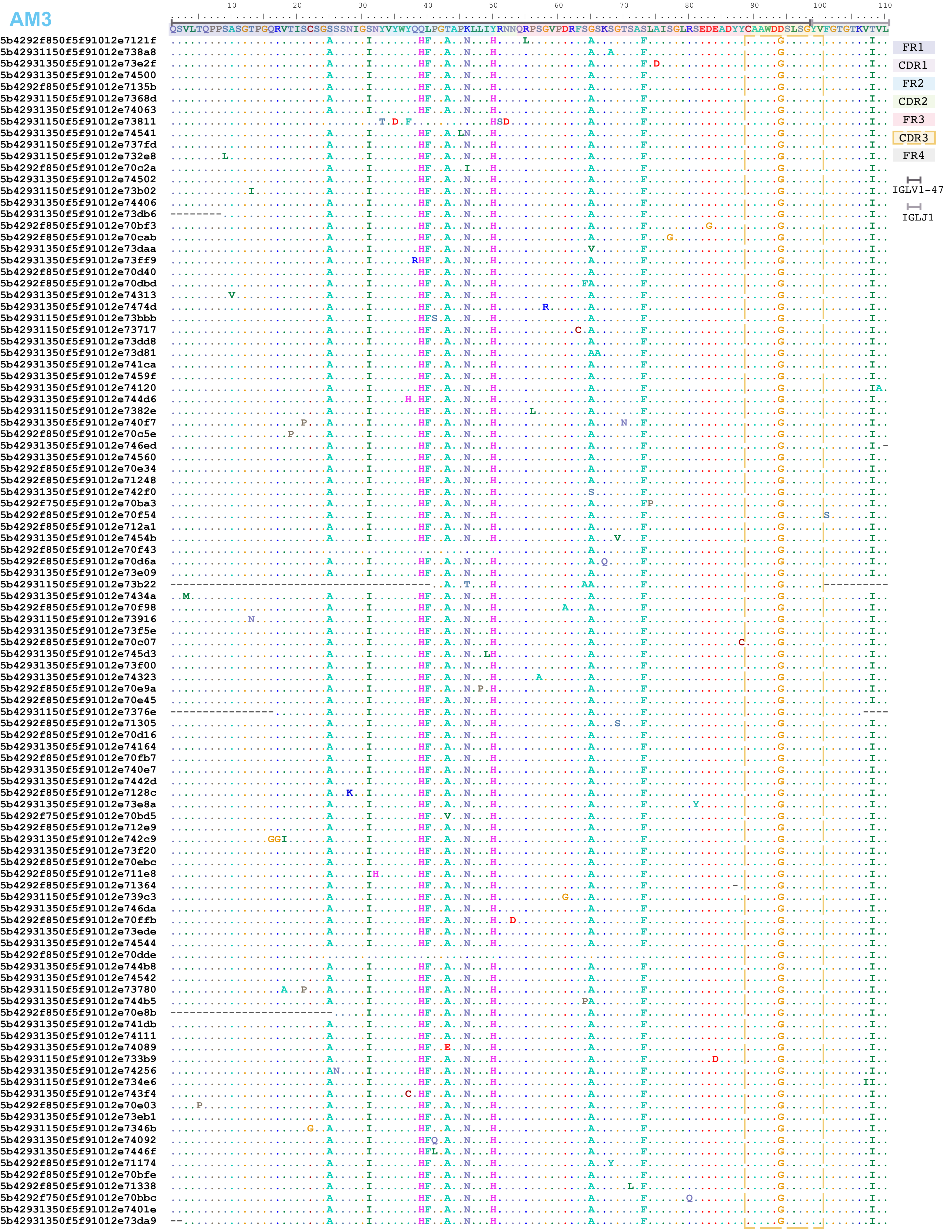
AM3
QSVLTQPPSASGTPGQRVTISCSGSSSNIGSNYVYWYQQLPGTAPKLLIYRNNQRPSGVPDRFSGSKSGTSASLAISGLRSEDEADYYCAAWDDSLSGYVFGTGTKVTVL
FR1
CDR1
FR2
CDR2
FR3
CDR3
FR4
IGLV1-47
IGLJ1

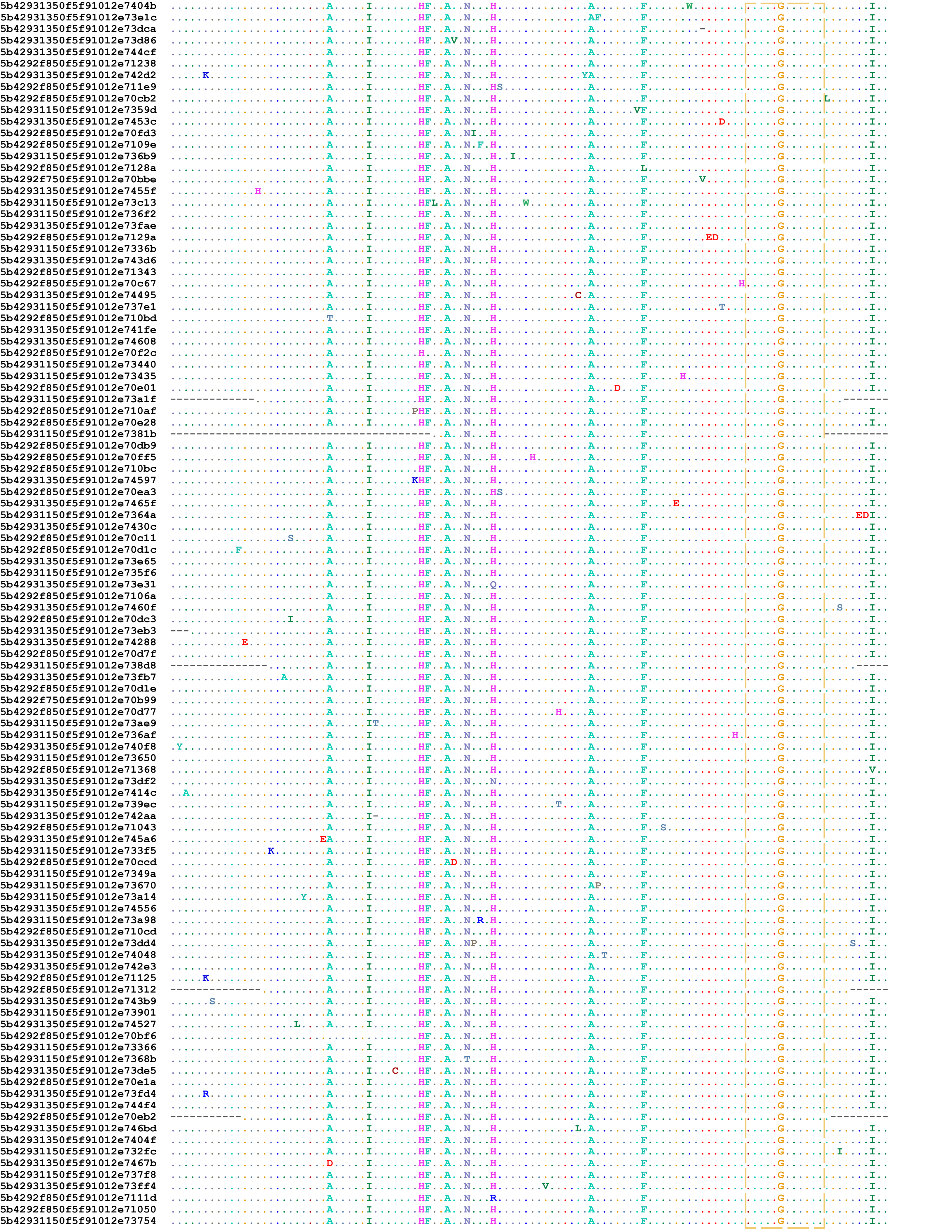
5b42931350f5f91012e7404b
5b42931350f5f91012e73e1c
5b42931350f5f91012e73dca
5b42931350f5f91012e73d86
5b42931350f5f91012e744cf
5b4292f850f5f91012e71238
5b42931350f5f91012e742d2
5b4292f850f5f91012e711e9
5b4292f850f5f91012e70cb2
5b42931150f5f91012e7359d
5b42931350f5f91012e7453c
5b4292f850f5f91012e70fd3
5b4292f850f5f91012e7109e
5b42931150f5f91012e736b9
5b4292f850f5f91012e7128a
5b4292f750f5f91012e70bbe
5b42931350f5f91012e7455f
5b42931150f5f91012e73c13
5b42931150f5f91012e736f2
5b42931350f5f91012e73fae
5b4292f850f5f91012e7129a
5b42931150f5f91012e7336b
5b42931350f5f91012e743d6
5b4292f850f5f91012e71343
5b4292f850f5f91012e70c67
5b42931350f5f91012e74495
5b42931150f5f91012e737e1
5b4292f850f5f91012e710bd
5b42931350f5f91012e741fe
5b42931350f5f91012e74608
5b4292f850f5f91012e70f2c
5b42931150f5f91012e73440
5b42931150f5f91012e73435
5b4292f850f5f91012e70e01
5b42931150f5f91012e73a1f
5b4292f850f5f91012e710af
5b4292f850f5f91012e70e28
5b42931150f5f91012e7381b
5b4292f850f5f91012e70db9
5b4292f850f5f91012e70ff5
5b4292f850f5f91012e710bc
5b42931350f5f91012e74597
5b4292f850f5f91012e70ea3
5b42931350f5f91012e7465f
5b42931150f5f91012e7364a
5b42931350f5f91012e7430c
5b4292f850f5f91012e70c11
5b4292f850f5f91012e70d1c
5b42931350f5f91012e73e65
5b42931150f5f91012e735f6
5b42931350f5f91012e73e31
5b4292f850f5f91012e7106a
5b42931350f5f91012e7460f
5b4292f850f5f91012e70dc3
5b42931350f5f91012e73eb3
5b42931350f5f91012e74288
5b4292f850f5f91012e70d7f
5b42931150f5f91012e738d8
5b42931350f5f91012e73fb7
5b4292f850f5f91012e70d1e
5b4292f750f5f91012e70b99
5b4292f850f5f91012e70d77
5b42931150f5f91012e73ae9
5b42931150f5f91012e736af
5b42931350f5f91012e740f8
5b42931150f5f91012e73650
5b4292f850f5f91012e71368
5b42931350f5f91012e73df2
5b42931350f5f91012e7414c
5b42931150f5f91012e739ec
5b42931350f5f91012e742aa
5b4292f850f5f91012e71043
5b42931350f5f91012e745a6
5b42931150f5f91012e733f5
5b4292f850f5f91012e70ccd
5b42931150f5f91012e7349a
5b42931150f5f91012e73670
5b42931150f5f91012e73a14
5b42931350f5f91012e74556
5b42931150f5f91012e73a98
5b4292f850f5f91012e710cd
5b42931350f5f91012e73dd4
5b42931350f5f91012e74048
5b42931350f5f91012e742e3
5b4292f850f5f91012e71125
5b4292f850f5f91012e71312
5b42931350f5f91012e743b9
5b42931150f5f91012e73901
5b42931350f5f91012e74527
5b4292f850f5f91012e70bf6
5b42931150f5f91012e73366
5b42931150f5f91012e7368b
5b42931350f5f91012e73de5
5b4292f850f5f91012e70e1a
5b42931350f5f91012e73fd4
5b42931350f5f91012e744f4
5b4292f850f5f91012e70eb2
5b42931350f5f91012e746bd
5b42931350f5f91012e7404f
5b42931150f5f91012e732fc
5b42931350f5f91012e7467b
5b42931150f5f91012e737f8
5b42931350f5f91012e73ff4
5b4292f850f5f91012e7111d
5b4292f850f5f91012e71050
5b42931150f5f91012e73754

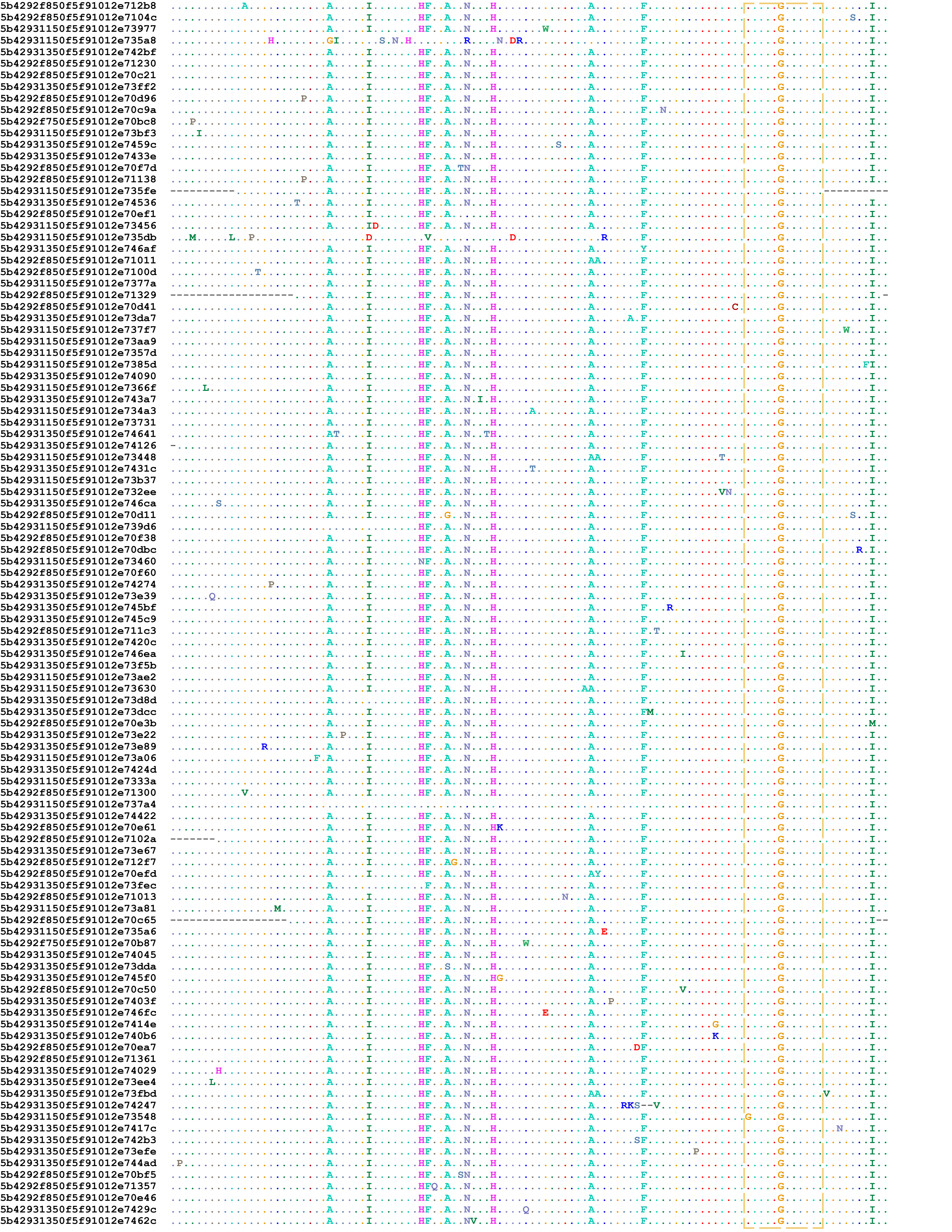
5b4292f850f5f91012e712b8
5b4292f850f5f91012e7104c
5b42931150f5f91012e73977
5b42931150f5f91012e735a8
5b42931350f5f91012e742bf
5b4292f850f5f91012e71230
5b4292f850f5f91012e70c21
5b42931350f5f91012e73ff2
5b4292f850f5f91012e70d96
5b4292f850f5f91012e70c9a
5b4292f750f5f91012e70bc8
5b42931150f5f91012e73bf3
5b42931350f5f91012e7459c
5b42931350f5f91012e7433e
5b4292f850f5f91012e70f7d
5b4292f850f5f91012e71138
5b42931150f5f91012e735fe
5b42931350f5f91012e74536
5b4292f850f5f91012e70ef1
5b42931350f5f91012e73456
5b42931150f5f91012e735db
5b42931350f5f91012e746af
5b4292f850f5f91012e71011
5b4292f850f5f91012e7100d
5b42931150f5f91012e7377a
5b4292f850f5f91012e71329
5b4292f850f5f91012e70d41
5b42931350f5f91012e73da7
5b42931150f5f91012e737f7
5b42931150f5f91012e73aa9
5b42931150f5f91012e7357d
5b42931150f5f91012e7385d
5b42931350f5f91012e74090
5b42931150f5f91012e7366f
5b42931350f5f91012e743a7
5b42931150f5f91012e734a3
5b42931150f5f91012e73731
5b42931350f5f91012e74641
5b42931350f5f91012e74126
5b42931150f5f91012e73448
5b42931350f5f91012e7431c
5b42931150f5f91012e73b37
5b42931150f5f91012e732ee
5b42931350f5f91012e746ca
5b4292f850f5f91012e70d11
5b42931150f5f91012e739d6
5b4292f850f5f91012e70f38
5b4292f850f5f91012e70dbc
5b42931150f5f91012e73460
5b4292f850f5f91012e70f60
5b42931350f5f91012e74274
5b42931350f5f91012e73e39
5b42931350f5f91012e745bf
5b42931350f5f91012e745c9
5b4292f850f5f91012e711c3
5b42931350f5f91012e7420c
5b42931350f5f91012e746ea
5b42931350f5f91012e73f5b
5b42931150f5f91012e73ae2
5b42931150f5f91012e73630
5b42931350f5f91012e73d8d
5b42931350f5f91012e73dcc
5b4292f850f5f91012e70e3b
5b42931350f5f91012e73e22
5b42931350f5f91012e73e89
5b42931150f5f91012e73a06
5b42931350f5f91012e7424d
5b42931150f5f91012e7333a
5b4292f850f5f91012e71300
5b42931150f5f91012e737a4
5b42931350f5f91012e74422
5b4292f850f5f91012e70e61
5b4292f850f5f91012e7102a
5b42931350f5f91012e73e67
5b4292f850f5f91012e712f7
5b4292f850f5f91012e70efd
5b42931350f5f91012e73fec
5b4292f850f5f91012e71013
5b42931150f5f91012e73a81
5b4292f850f5f91012e70c65
5b42931150f5f91012e735a6
5b4292f750f5f91012e70b87
5b42931350f5f91012e74045
5b42931350f5f91012e73dda
5b42931350f5f91012e745f0
5b4292f850f5f91012e70c50
5b42931350f5f91012e7403f
5b42931350f5f91012e746fc
5b42931350f5f91012e7414e
5b42931350f5f91012e740b6
5b4292f850f5f91012e70ea7
5b4292f850f5f91012e71361
5b42931350f5f91012e74029
5b42931350f5f91012e73ee4
5b42931350f5f91012e73fbd
5b42931350f5f91012e74247
5b42931150f5f91012e73548
5b42931350f5f91012e7417c
5b42931350f5f91012e742b3
5b42931350f5f91012e73efe
5b42931350f5f91012e744ad
5b4292f850f5f91012e70bf5
5b4292f850f5f91012e71357
5b4292f850f5f91012e70e46
5b42931350f5f91012e7429c
5b42931350f5f91012e7462c

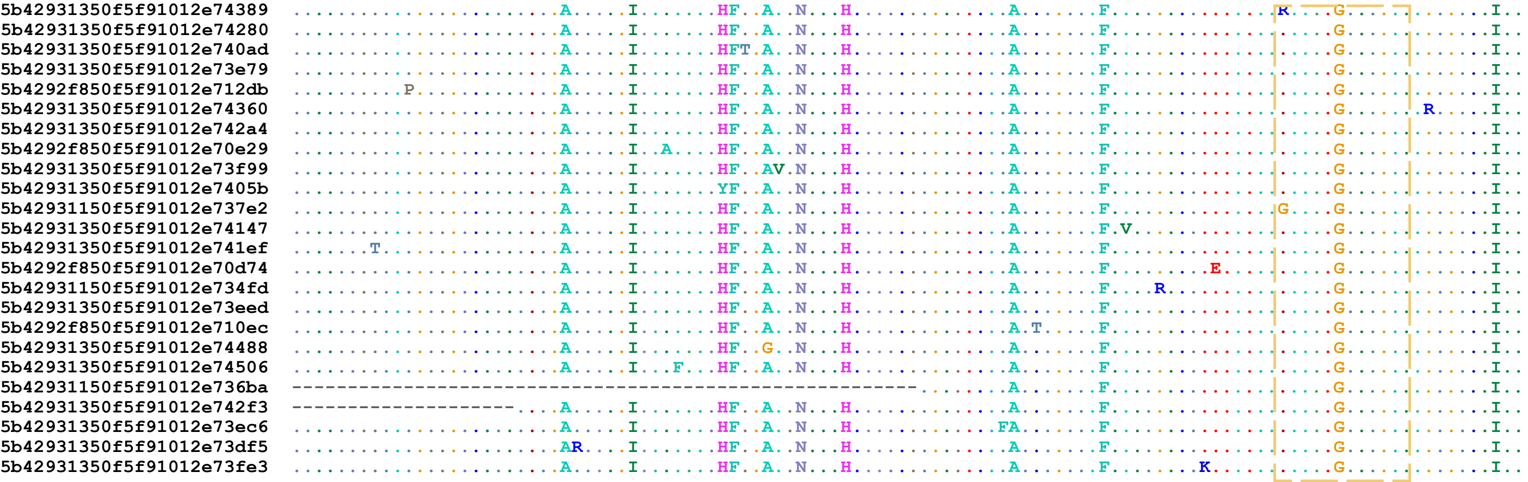
5b42931350f5f91012e74389
5b42931350f5f91012e74280
5b42931350f5f91012e740ad
5b42931350f5f91012e73e79
5b4292f850f5f91012e712db
5b42931350f5f91012e74360
5b42931350f5f91012e742a4
5b4292f850f5f91012e70e29
5b42931350f5f91012e73f99
5b42931350f5f91012e7405b
5b42931150f5f91012e737e2
5b42931350f5f91012e74147
5b42931350f5f91012e741ef
5b4292f850f5f91012e70d74
5b42931150f5f91012e734fd
5b42931350f5f91012e73eed
5b4292f850f5f91012e710ec
5b42931350f5f91012e74488
5b42931350f5f91012e74506
5b42931150f5f91012e736ba
5b42931350f5f91012e742f3
5b42931350f5f91012e73ec6
5b42931350f5f91012e73df5
5b42931350f5f91012e73fe3
