# Supplementary figures and images for: Diverse patterns of antibody variable gene repertoire disruption in patients with amyloid light chain (AL) amyloidosis
Source: PLoS One. 2020 Jul 7;15(7):e0235713. doi: 10.1371/journal.pone.0235713 (PMC7340310; doi:10.1371/journal.pone.0235713)

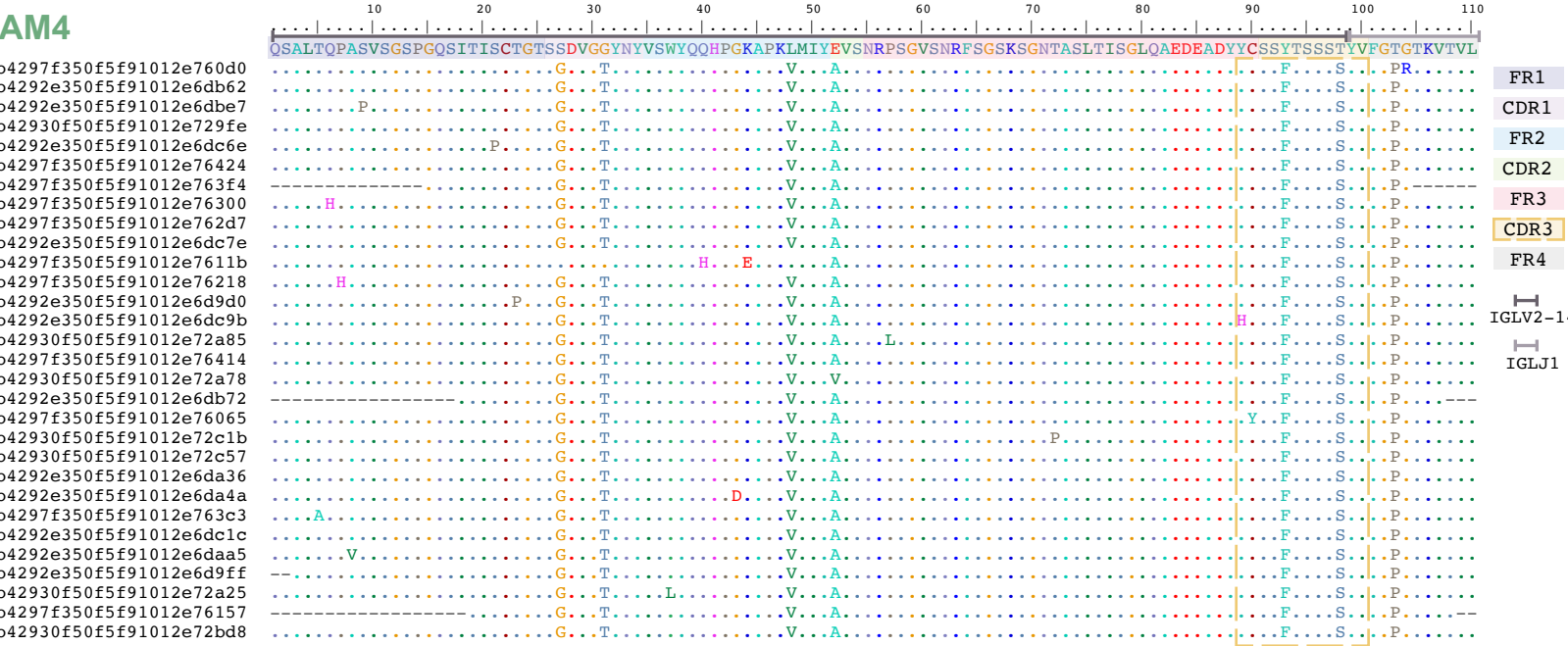

Supplement: S6 Fig — Somatic variants of the dominant clone were aligned to inferred germline genes to create a multiple sequence alignment. (PDF) [file pone.0235713.s008.pdf]
