# Supplementary material for: Diverse patterns of antibody variable gene repertoire disruption in patients with amyloid light chain (AL) amyloidosis
Source: PLoS One. 2020 Jul 7;15(7):e0235713. doi: 10.1371/journal.pone.0235713 (PMC7340310; doi:10.1371/journal.pone.0235713)
Supplement: S7 Fig — Somatic variants of the dominant clone were aligned to inferred germline genes to create a multiple sequence alignment. (PDF) [file pone.0235713.s009.pdf]

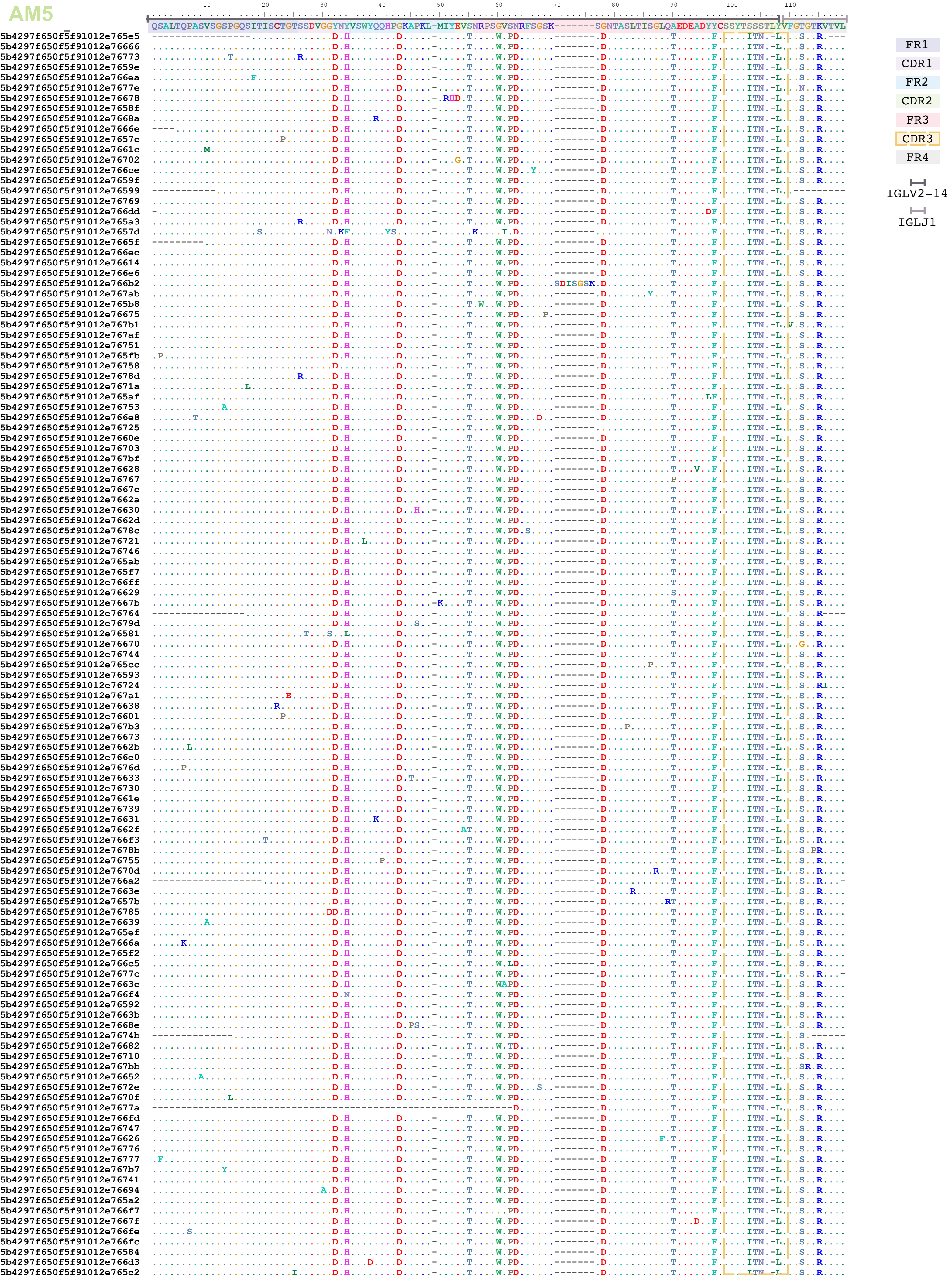
AM5
10 20 30 40 50 60 70 80 90 100 110
QSALTQPASVSGSPGQSITISCTGTSSDVGGYNYVSWYQQHPGKAPKL-MIYEVSNRPSGVSNRFSGSK-------SGNTASLTISGLQAEDEADYYCSSYTSSSTLYVFGTGTKVTVL
FR1
CDR1
FR2
CDR2
FR3
CDR3
FR4
IGLV2-14
IGLJ1

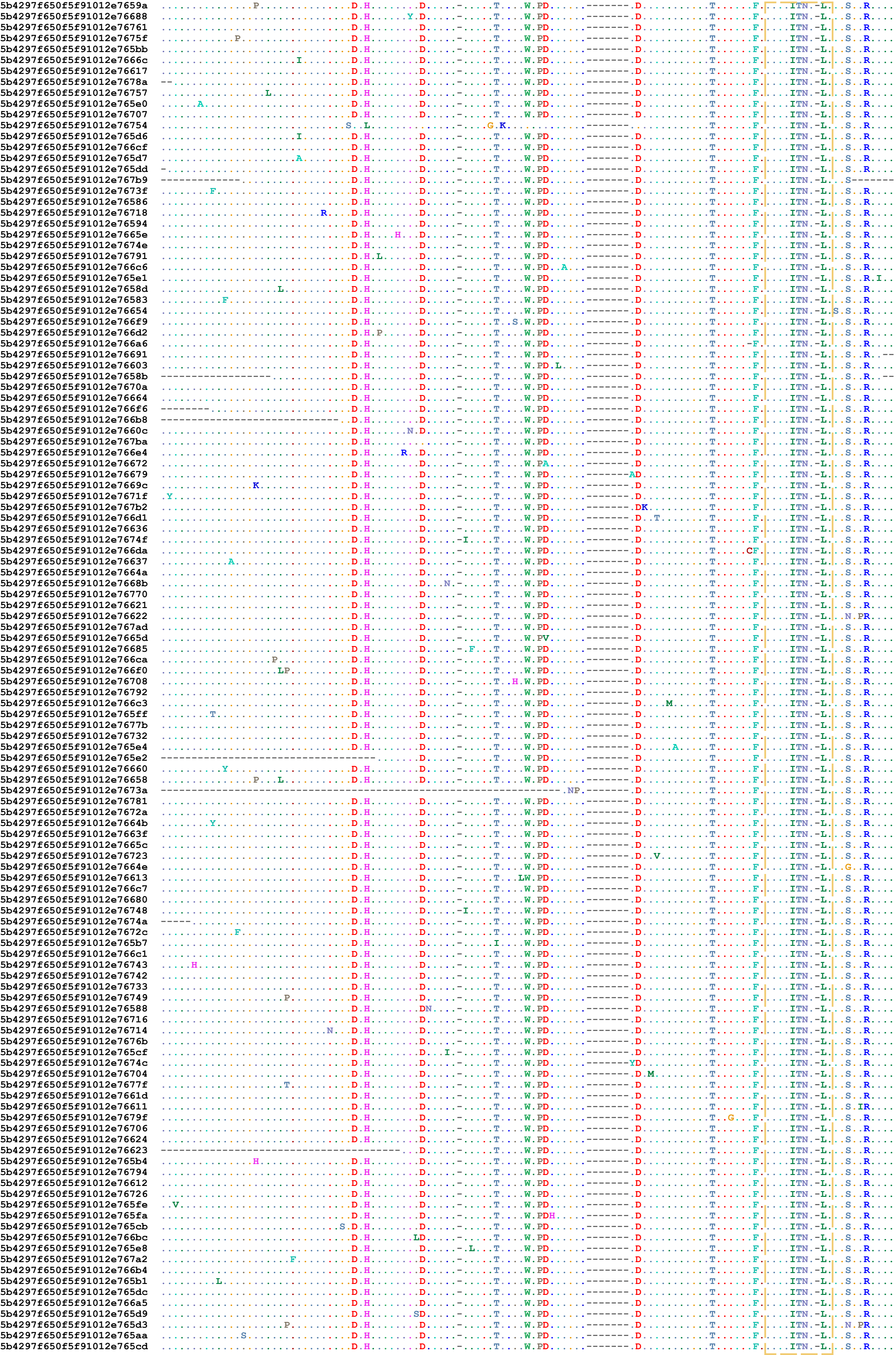
5b4297f650f5f91012e7659a
5b4297f650f5f91012e76688
5b4297f650f5f91012e76761
5b4297f650f5f91012e7675f
5b4297f650f5f91012e765bb
5b4297f650f5f91012e7666c
5b4297f650f5f91012e76617
5b4297f650f5f91012e7678a
5b4297f650f5f91012e76757
5b4297f650f5f91012e765e0
5b4297f650f5f91012e76707
5b4297f650f5f91012e76754
5b4297f650f5f91012e765d6
5b4297f650f5f91012e766cf
5b4297f650f5f91012e765d7
5b4297f650f5f91012e765dd
5b4297f650f5f91012e767b9
5b4297f650f5f91012e7673f
5b4297f650f5f91012e76586
5b4297f650f5f91012e76718
5b4297f650f5f91012e76594
5b4297f650f5f91012e7665e
5b4297f650f5f91012e7674e
5b4297f650f5f91012e76791
5b4297f650f5f91012e766c6
5b4297f650f5f91012e765e1
5b4297f650f5f91012e7658d
5b4297f650f5f91012e76583
5b4297f650f5f91012e76654
5b4297f650f5f91012e766f9
5b4297f650f5f91012e766d2
5b4297f650f5f91012e766a6
5b4297f650f5f91012e76691
5b4297f650f5f91012e76603
5b4297f650f5f91012e7658b
5b4297f650f5f91012e7670a
5b4297f650f5f91012e76664
5b4297f650f5f91012e766f6
5b4297f650f5f91012e766b8
5b4297f650f5f91012e7660c
5b4297f650f5f91012e767ba
5b4297f650f5f91012e766e4
5b4297f650f5f91012e76672
5b4297f650f5f91012e76679
5b4297f650f5f91012e7669c
5b4297f650f5f91012e7671f
5b4297f650f5f91012e767b2
5b4297f650f5f91012e766d1
5b4297f650f5f91012e76636
5b4297f650f5f91012e7674f
5b4297f650f5f91012e766da
5b4297f650f5f91012e76637
5b4297f650f5f91012e7664a
5b4297f650f5f91012e7668b
5b4297f650f5f91012e76770
5b4297f650f5f91012e76621
5b4297f650f5f91012e76622
5b4297f650f5f91012e767ad
5b4297f650f5f91012e7665d
5b4297f650f5f91012e76685
5b4297f650f5f91012e766ca
5b4297f650f5f91012e766f0
5b4297f650f5f91012e76708
5b4297f650f5f91012e76792
5b4297f650f5f91012e766c3
5b4297f650f5f91012e765ff
5b4297f650f5f91012e7677b
5b4297f650f5f91012e76732
5b4297f650f5f91012e765e4
5b4297f650f5f91012e765e2
5b4297f650f5f91012e76660
5b4297f650f5f91012e76658
5b4297f650f5f91012e7673a
5b4297f650f5f91012e76781
5b4297f650f5f91012e7672a
5b4297f650f5f91012e7664b
5b4297f650f5f91012e7663f
5b4297f650f5f91012e7665c
5b4297f650f5f91012e76723
5b4297f650f5f91012e7664e
5b4297f650f5f91012e76613
5b4297f650f5f91012e766c7
5b4297f650f5f91012e76680
5b4297f650f5f91012e76748
5b4297f650f5f91012e7674a
5b4297f650f5f91012e7672c
5b4297f650f5f91012e765b7
5b4297f650f5f91012e766c1
5b4297f650f5f91012e76743
5b4297f650f5f91012e76742
5b4297f650f5f91012e76733
5b4297f650f5f91012e76749
5b4297f650f5f91012e76588
5b4297f650f5f91012e76716
5b4297f650f5f91012e76714
5b4297f650f5f91012e7676b
5b4297f650f5f91012e765cf
5b4297f650f5f91012e7674c
5b4297f650f5f91012e76704
5b4297f650f5f91012e7677f
5b4297f650f5f91012e7661d
5b4297f650f5f91012e76611
5b4297f650f5f91012e7679f
5b4297f650f5f91012e76706
5b4297f650f5f91012e76624
5b4297f650f5f91012e76623
5b4297f650f5f91012e765b4
5b4297f650f5f91012e76794
5b4297f650f5f91012e76612
5b4297f650f5f91012e76726
5b4297f650f5f91012e765fe
5b4297f650f5f91012e765fa
5b4297f650f5f91012e765cb
5b4297f650f5f91012e766bc
5b4297f650f5f91012e765e8
5b4297f650f5f91012e767a2
5b4297f650f5f91012e766b4
5b4297f650f5f91012e765b1
5b4297f650f5f91012e765dc
5b4297f650f5f91012e766a5
5b4297f650f5f91012e765d9
5b4297f650f5f91012e765d3
5b4297f650f5f91012e765aa
5b4297f650f5f91012e765cd

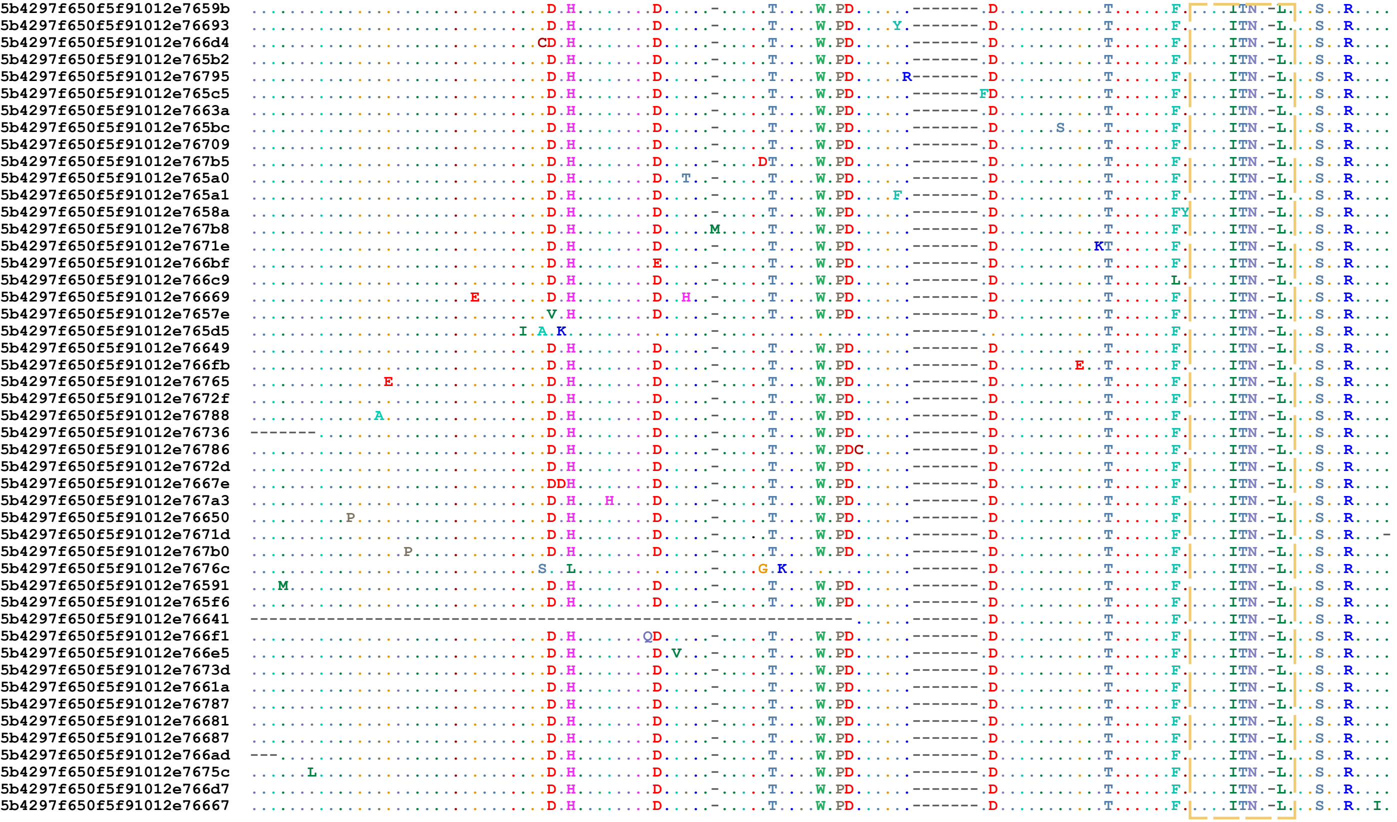
5b4297f650f5f91012e7659b
5b4297f650f5f91012e76693
5b4297f650f5f91012e766d4
5b4297f650f5f91012e765b2
5b4297f650f5f91012e76795
5b4297f650f5f91012e765c5
5b4297f650f5f91012e7663a
5b4297f650f5f91012e765bc
5b4297f650f5f91012e76709
5b4297f650f5f91012e767b5
5b4297f650f5f91012e765a0
5b4297f650f5f91012e765a1
5b4297f650f5f91012e7658a
5b4297f650f5f91012e767b8
5b4297f650f5f91012e7671e
5b4297f650f5f91012e766bf
5b4297f650f5f91012e766c9
5b4297f650f5f91012e76669
5b4297f650f5f91012e7657e
5b4297f650f5f91012e765d5
5b4297f650f5f91012e76649
5b4297f650f5f91012e766fb
5b4297f650f5f91012e76765
5b4297f650f5f91012e7672f
5b4297f650f5f91012e76788
5b4297f650f5f91012e76736
5b4297f650f5f91012e76786
5b4297f650f5f91012e7672d
5b4297f650f5f91012e7667e
5b4297f650f5f91012e767a3
5b4297f650f5f91012e76650
5b4297f650f5f91012e7671d
5b4297f650f5f91012e767b0
5b4297f650f5f91012e7676c
5b4297f650f5f91012e76591
5b4297f650f5f91012e765f6
5b4297f650f5f91012e76641
5b4297f650f5f91012e766f1
5b4297f650f5f91012e766e5
5b4297f650f5f91012e7673d
5b4297f650f5f91012e7661a
5b4297f650f5f91012e76787
5b4297f650f5f91012e76681
5b4297f650f5f91012e76687
5b4297f650f5f91012e766ad
5b4297f650f5f91012e7675c
5b4297f650f5f91012e766d7
5b4297f650f5f91012e76667
