# Supplementary material for: Diverse patterns of antibody variable gene repertoire disruption in patients with amyloid light chain (AL) amyloidosis
Source: PLoS One. 2020 Jul 7;15(7):e0235713. doi: 10.1371/journal.pone.0235713 (PMC7340310; doi:10.1371/journal.pone.0235713)
Supplement: S8 Fig — Somatic variants of the dominant clone were aligned to inferred germline genes to create a multiple sequence alignment. (PDF) [file pone.0235713.s010.pdf]

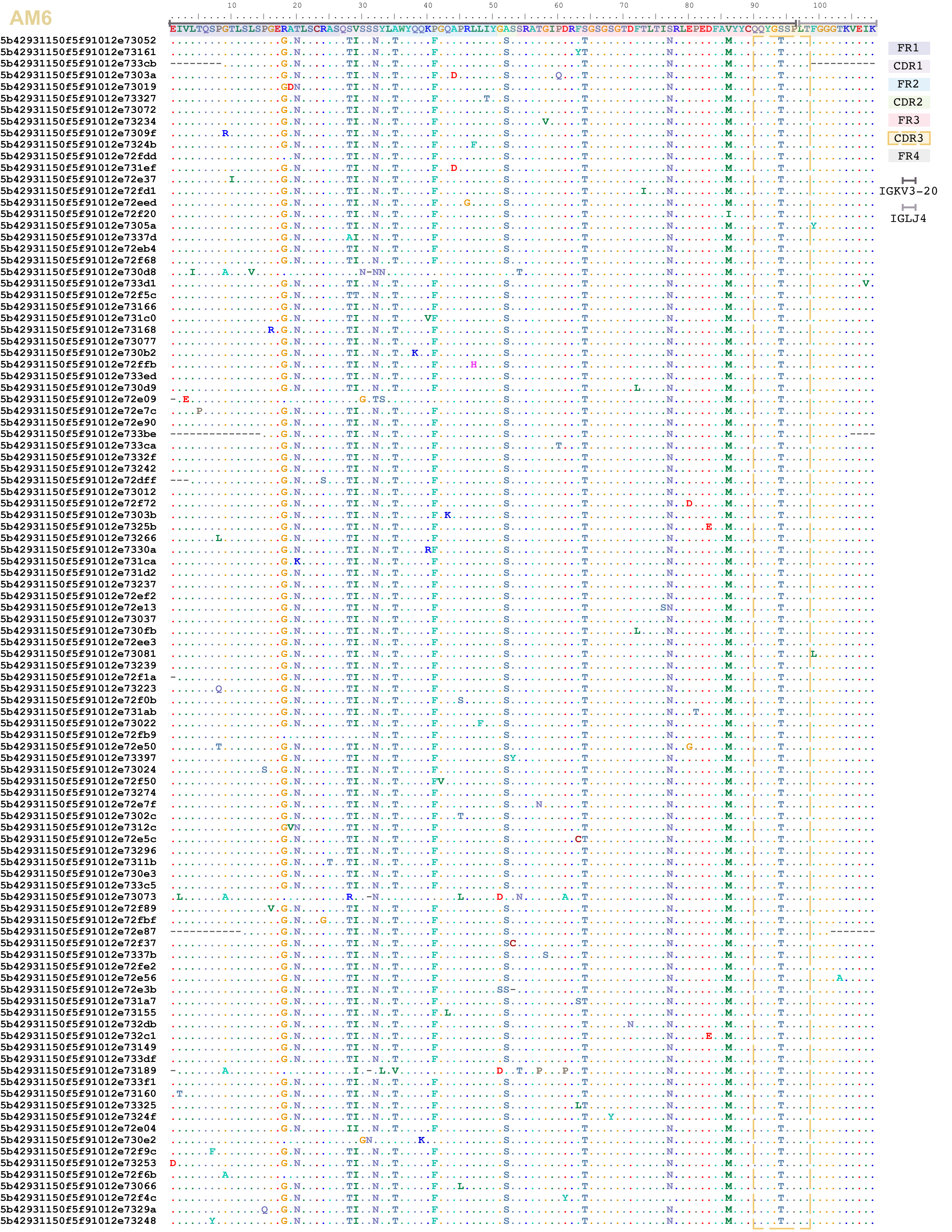
AM6
EIVLTQSPGTLSLSPGERATLSCRASQSVSSSYLAWYQQKPGQAPRLLIYGASSRATGIPDRFSGSGSGTDFTLTISRLEPEDFAVYYCQQYGSSPLTFGGGTKVEIK
FR1
CDR1
FR2
CDR2
FR3
CDR3
FR4
IGKV3-20
IGLJ4

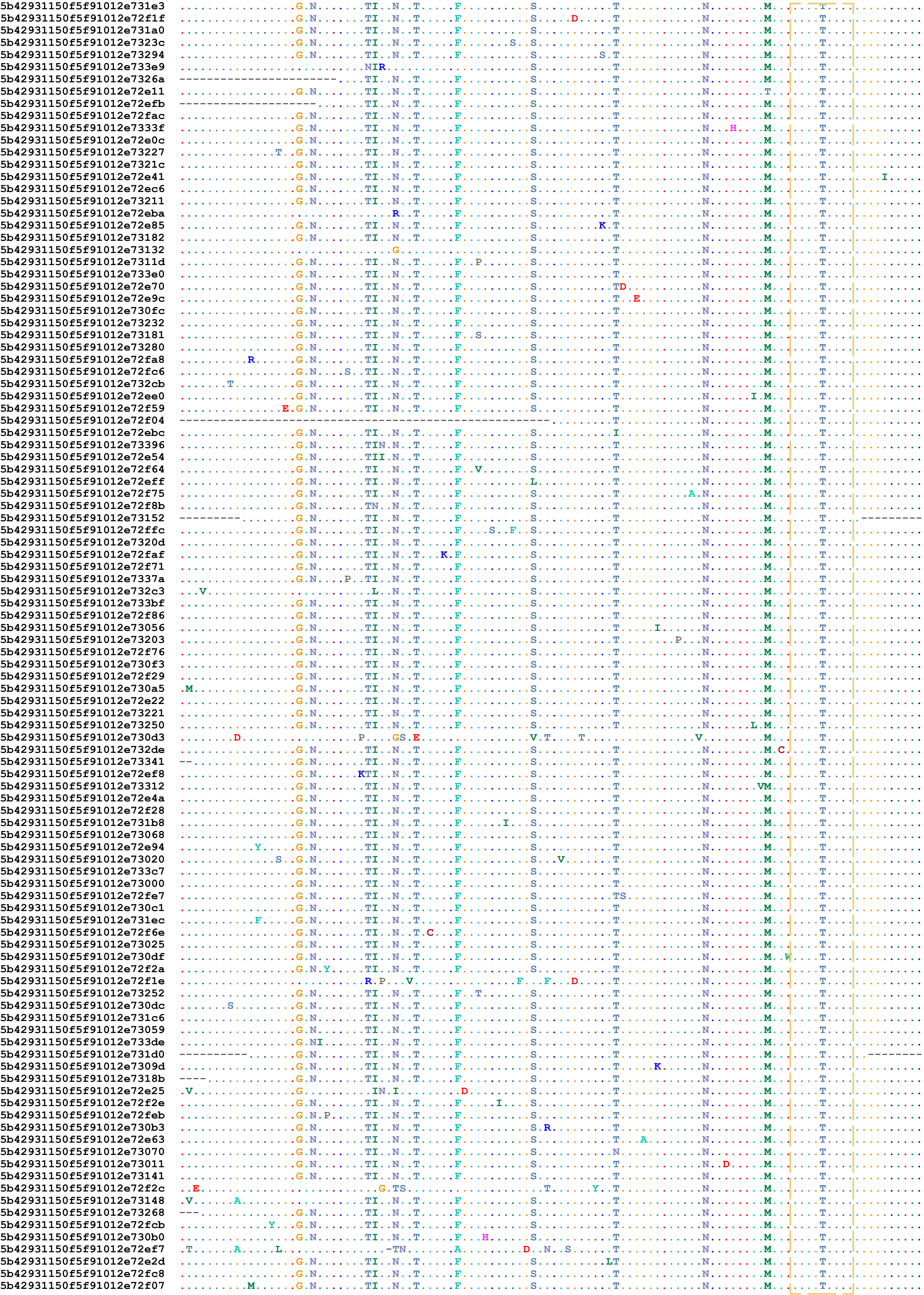
5b42931150f5f91012e731e3
5b42931150f5f91012e72f1f
5b42931150f5f91012e731a0
5b42931150f5f91012e7323c
5b42931150f5f91012e73294
5b42931150f5f91012e733e9
5b42931150f5f91012e7326a
5b42931150f5f91012e72e11
5b42931150f5f91012e72efb
5b42931150f5f91012e72fac
5b42931150f5f91012e7333f
5b42931150f5f91012e72e0c
5b42931150f5f91012e73227
5b42931150f5f91012e7321c
5b42931150f5f91012e72e41
5b42931150f5f91012e72ec6
5b42931150f5f91012e73211
5b42931150f5f91012e72eba
5b42931150f5f91012e72e85
5b42931150f5f91012e73182
5b42931150f5f91012e73132
5b42931150f5f91012e7311d
5b42931150f5f91012e733e0
5b42931150f5f91012e72e70
5b42931150f5f91012e72e9c
5b42931150f5f91012e730fc
5b42931150f5f91012e73232
5b42931150f5f91012e73181
5b42931150f5f91012e73280
5b42931150f5f91012e72fa8
5b42931150f5f91012e72fc6
5b42931150f5f91012e732cb
5b42931150f5f91012e72ee0
5b42931150f5f91012e72f59
5b42931150f5f91012e72f04
5b42931150f5f91012e72ebc
5b42931150f5f91012e73396
5b42931150f5f91012e72e54
5b42931150f5f91012e72f64
5b42931150f5f91012e72eff
5b42931150f5f91012e72f75
5b42931150f5f91012e72f8b
5b42931150f5f91012e73152
5b42931150f5f91012e72ffc
5b42931150f5f91012e7320d
5b42931150f5f91012e72faf
5b42931150f5f91012e72f71
5b42931150f5f91012e7337a
5b42931150f5f91012e732c3
5b42931150f5f91012e733bf
5b42931150f5f91012e72f86
5b42931150f5f91012e73056
5b42931150f5f91012e73203
5b42931150f5f91012e72f76
5b42931150f5f91012e730f3
5b42931150f5f91012e72f29
5b42931150f5f91012e730a5
5b42931150f5f91012e72e22
5b42931150f5f91012e73221
5b42931150f5f91012e73250
5b42931150f5f91012e730d3
5b42931150f5f91012e732de
5b42931150f5f91012e73341
5b42931150f5f91012e72ef8
5b42931150f5f91012e73312
5b42931150f5f91012e72e4a
5b42931150f5f91012e72f28
5b42931150f5f91012e731b8
5b42931150f5f91012e73068
5b42931150f5f91012e72e94
5b42931150f5f91012e73020
5b42931150f5f91012e733c7
5b42931150f5f91012e73000
5b42931150f5f91012e72fe7
5b42931150f5f91012e730c1
5b42931150f5f91012e731ec
5b42931150f5f91012e72f6e
5b42931150f5f91012e73025
5b42931150f5f91012e730df
5b42931150f5f91012e72f2a
5b42931150f5f91012e72f1e
5b42931150f5f91012e73252
5b42931150f5f91012e730dc
5b42931150f5f91012e731c6
5b42931150f5f91012e73059
5b42931150f5f91012e733de
5b42931150f5f91012e731d0
5b42931150f5f91012e7309d
5b42931150f5f91012e7318b
5b42931150f5f91012e72e25
5b42931150f5f91012e72f2e
5b42931150f5f91012e72feb
5b42931150f5f91012e730b3
5b42931150f5f91012e72e63
5b42931150f5f91012e73070
5b42931150f5f91012e73011
5b42931150f5f91012e73141
5b42931150f5f91012e72f2c
5b42931150f5f91012e73148
5b42931150f5f91012e73268
5b42931150f5f91012e72fcb
5b42931150f5f91012e730b0
5b42931150f5f91012e72ef7
5b42931150f5f91012e72e2d
5b42931150f5f91012e72fc8
5b42931150f5f91012e72f07

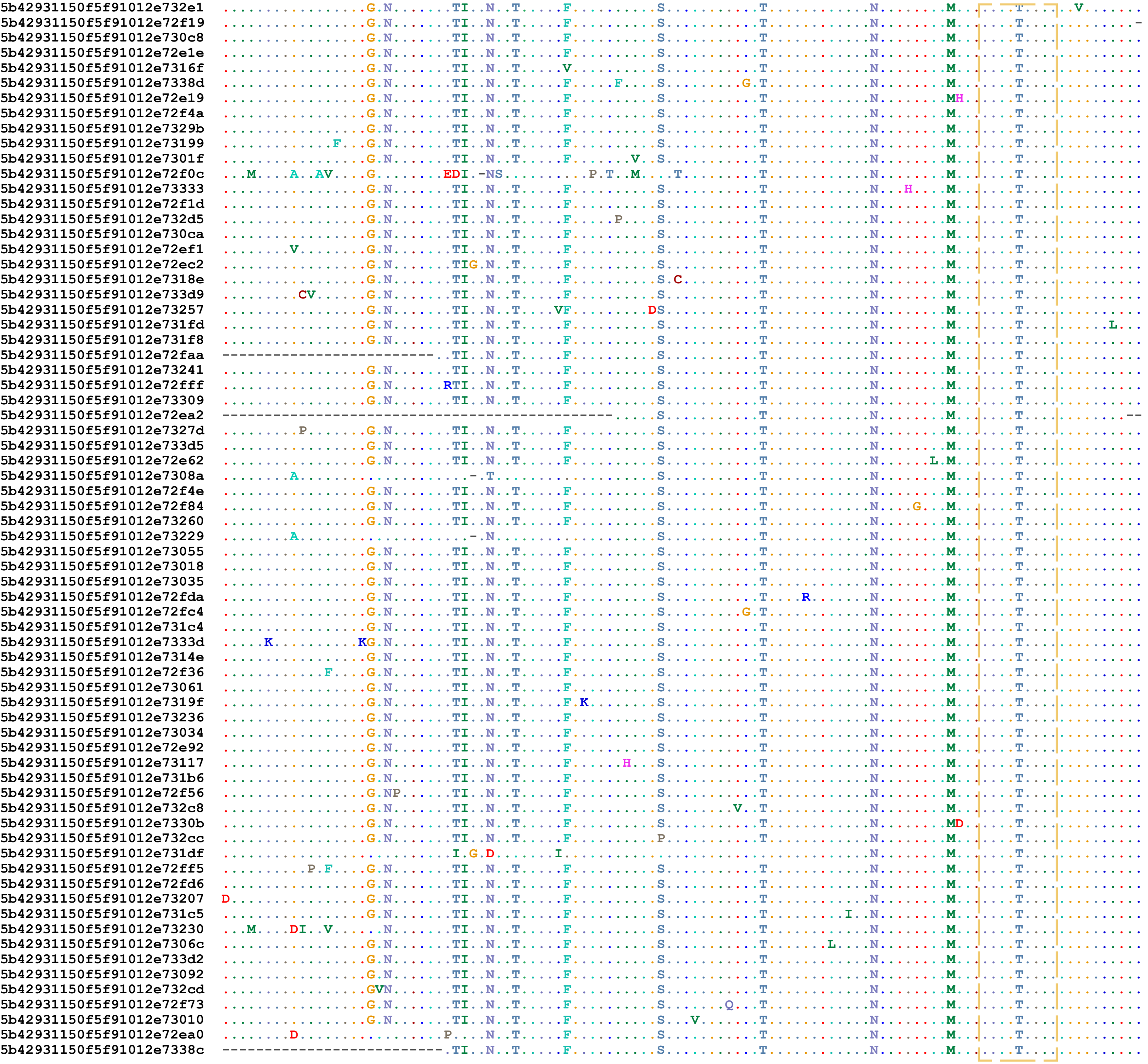
5b42931150f5f91012e732e1
5b42931150f5f91012e72f19
5b42931150f5f91012e730c8
5b42931150f5f91012e72e1e
5b42931150f5f91012e7316f
5b42931150f5f91012e7338d
5b42931150f5f91012e72e19
5b42931150f5f91012e72f4a
5b42931150f5f91012e7329b
5b42931150f5f91012e73199
5b42931150f5f91012e7301f
5b42931150f5f91012e72f0c
5b42931150f5f91012e73333
5b42931150f5f91012e72f1d
5b42931150f5f91012e732d5
5b42931150f5f91012e730ca
5b42931150f5f91012e72ef1
5b42931150f5f91012e72ec2
5b42931150f5f91012e7318e
5b42931150f5f91012e733d9
5b42931150f5f91012e73257
5b42931150f5f91012e731fd
5b42931150f5f91012e731f8
5b42931150f5f91012e72faa
5b42931150f5f91012e73241
5b42931150f5f91012e72fff
5b42931150f5f91012e73309
5b42931150f5f91012e72ea2
5b42931150f5f91012e7327d
5b42931150f5f91012e733d5
5b42931150f5f91012e72e62
5b42931150f5f91012e7308a
5b42931150f5f91012e72f4e
5b42931150f5f91012e72f84
5b42931150f5f91012e73260
5b42931150f5f91012e73229
5b42931150f5f91012e73055
5b42931150f5f91012e73018
5b42931150f5f91012e73035
5b42931150f5f91012e72fda
5b42931150f5f91012e72fc4
5b42931150f5f91012e731c4
5b42931150f5f91012e7333d
5b42931150f5f91012e7314e
5b42931150f5f91012e72f36
5b42931150f5f91012e73061
5b42931150f5f91012e7319f
5b42931150f5f91012e73236
5b42931150f5f91012e73034
5b42931150f5f91012e72e92
5b42931150f5f91012e73117
5b42931150f5f91012e731b6
5b42931150f5f91012e72f56
5b42931150f5f91012e732c8
5b42931150f5f91012e7330b
5b42931150f5f91012e732cc
5b42931150f5f91012e731df
5b42931150f5f91012e72ff5
5b42931150f5f91012e72fd6
5b42931150f5f91012e73207
5b42931150f5f91012e731c5
5b42931150f5f91012e73230
5b42931150f5f91012e7306c
5b42931150f5f91012e733d2
5b42931150f5f91012e73092
5b42931150f5f91012e732cd
5b42931150f5f91012e72f73
5b42931150f5f91012e73010
5b42931150f5f91012e72ea0
5b42931150f5f91012e7338c
