# Supplementary material for: Diverse patterns of antibody variable gene repertoire disruption in patients with amyloid light chain (AL) amyloidosis
Source: PLoS One. 2020 Jul 7;15(7):e0235713. doi: 10.1371/journal.pone.0235713 (PMC7340310; doi:10.1371/journal.pone.0235713)
Supplement: S9 Fig — Somatic variants of the dominant clone were aligned to inferred germline genes to create a multiple sequence alignment. (PDF) [file pone.0235713.s011.pdf]

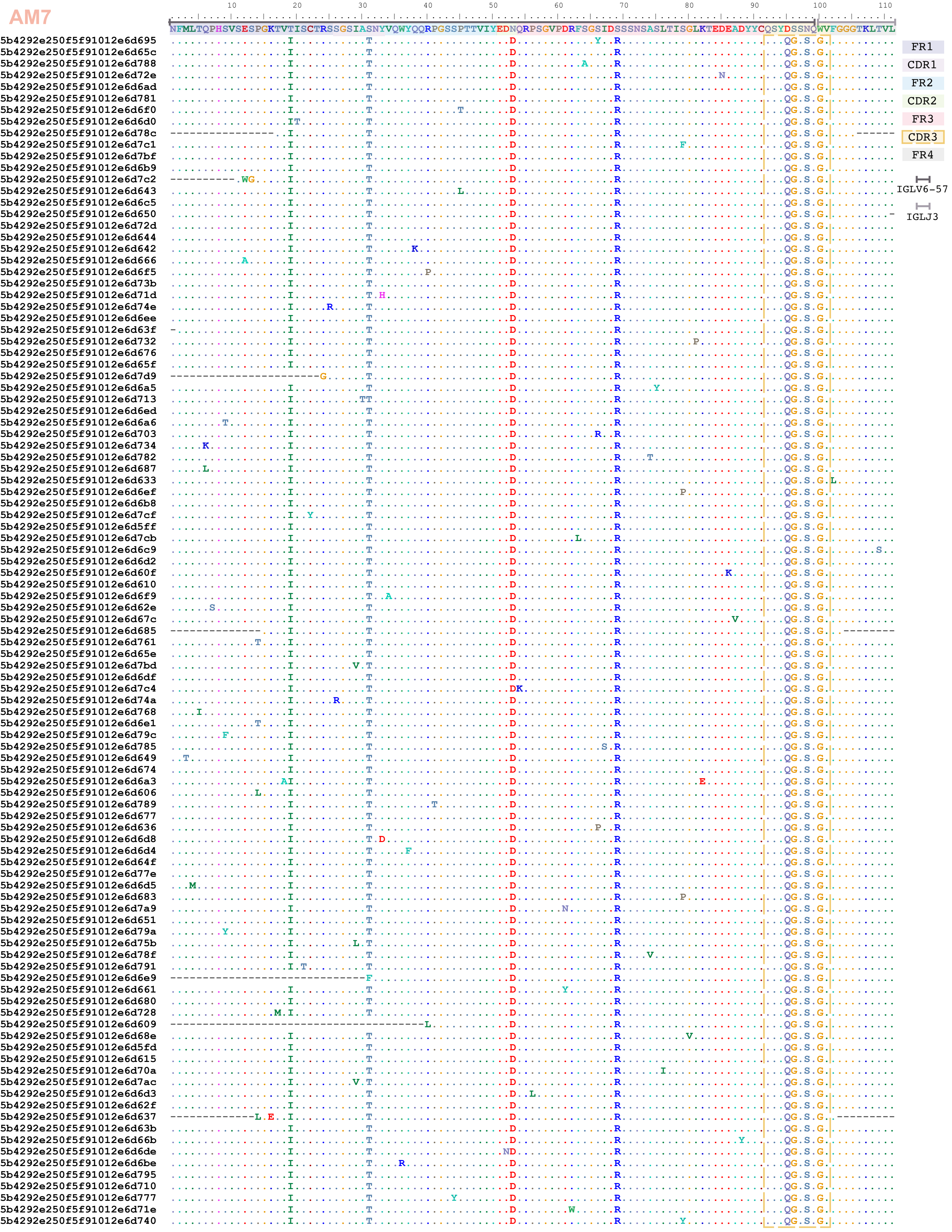
AM7
10 20 30 40 50 60 70 80 90 100 110
NFMLTQPHSVSESPGKTVTISCTRSSGSIASNYVQWYQQRPGSSPTTVIYEDNQRPSGVPDRFSGSIDSSSNSASLTISGLKTEDEADYYCQSYDSSNQWVFGGGTKLTVL
FR1
CDR1
FR2
CDR2
FR3
CDR3
FR4
IGLV6-57
IGLJ3

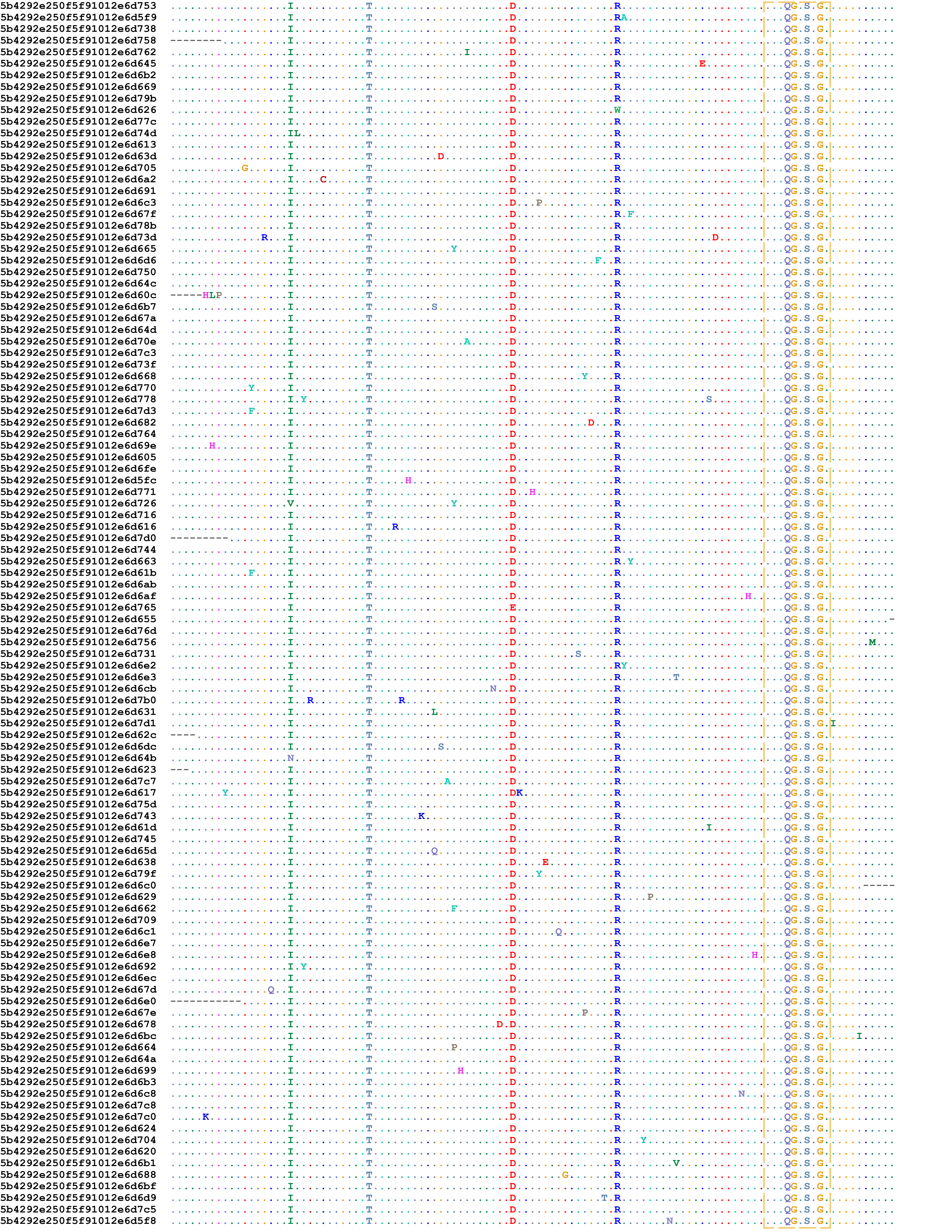
5b4292e250f5f91012e6d753
5b4292e250f5f91012e6d5f9
5b4292e250f5f91012e6d738
5b4292e250f5f91012e6d758
5b4292e250f5f91012e6d762
5b4292e250f5f91012e6d645
5b4292e250f5f91012e6d6b2
5b4292e250f5f91012e6d669
5b4292e250f5f91012e6d79b
5b4292e250f5f91012e6d626
5b4292e250f5f91012e6d77c
5b4292e250f5f91012e6d74d
5b4292e250f5f91012e6d613
5b4292e250f5f91012e6d63d
5b4292e250f5f91012e6d705
5b4292e250f5f91012e6d6a2
5b4292e250f5f91012e6d691
5b4292e250f5f91012e6d6c3
5b4292e250f5f91012e6d67f
5b4292e250f5f91012e6d78b
5b4292e250f5f91012e6d73d
5b4292e250f5f91012e6d665
5b4292e250f5f91012e6d6d6
5b4292e250f5f91012e6d750
5b4292e250f5f91012e6d64c
5b4292e250f5f91012e6d60c
5b4292e250f5f91012e6d6b7
5b4292e250f5f91012e6d67a
5b4292e250f5f91012e6d64d
5b4292e250f5f91012e6d70e
5b4292e250f5f91012e6d7c3
5b4292e250f5f91012e6d73f
5b4292e250f5f91012e6d668
5b4292e250f5f91012e6d770
5b4292e250f5f91012e6d778
5b4292e250f5f91012e6d7d3
5b4292e250f5f91012e6d682
5b4292e250f5f91012e6d764
5b4292e250f5f91012e6d69e
5b4292e250f5f91012e6d605
5b4292e250f5f91012e6d6fe
5b4292e250f5f91012e6d5fc
5b4292e250f5f91012e6d771
5b4292e250f5f91012e6d726
5b4292e250f5f91012e6d716
5b4292e250f5f91012e6d616
5b4292e250f5f91012e6d7d0
5b4292e250f5f91012e6d744
5b4292e250f5f91012e6d663
5b4292e250f5f91012e6d61b
5b4292e250f5f91012e6d6ab
5b4292e250f5f91012e6d6af
5b4292e250f5f91012e6d765
5b4292e250f5f91012e6d655
5b4292e250f5f91012e6d76d
5b4292e250f5f91012e6d756
5b4292e250f5f91012e6d731
5b4292e250f5f91012e6d6e2
5b4292e250f5f91012e6d6e3
5b4292e250f5f91012e6d6cb
5b4292e250f5f91012e6d7b0
5b4292e250f5f91012e6d631
5b4292e250f5f91012e6d7d1
5b4292e250f5f91012e6d62c
5b4292e250f5f91012e6d6dc
5b4292e250f5f91012e6d64b
5b4292e250f5f91012e6d623
5b4292e250f5f91012e6d7c7
5b4292e250f5f91012e6d617
5b4292e250f5f91012e6d75d
5b4292e250f5f91012e6d743
5b4292e250f5f91012e6d61d
5b4292e250f5f91012e6d745
5b4292e250f5f91012e6d65d
5b4292e250f5f91012e6d638
5b4292e250f5f91012e6d79f
5b4292e250f5f91012e6d6c0
5b4292e250f5f91012e6d629
5b4292e250f5f91012e6d662
5b4292e250f5f91012e6d709
5b4292e250f5f91012e6d6c1
5b4292e250f5f91012e6d6e7
5b4292e250f5f91012e6d6e8
5b4292e250f5f91012e6d692
5b4292e250f5f91012e6d6ec
5b4292e250f5f91012e6d67d
5b4292e250f5f91012e6d6e0
5b4292e250f5f91012e6d67e
5b4292e250f5f91012e6d678
5b4292e250f5f91012e6d6bc
5b4292e250f5f91012e6d664
5b4292e250f5f91012e6d64a
5b4292e250f5f91012e6d699
5b4292e250f5f91012e6d6b3
5b4292e250f5f91012e6d6c8
5b4292e250f5f91012e6d7c8
5b4292e250f5f91012e6d7c0
5b4292e250f5f91012e6d624
5b4292e250f5f91012e6d704
5b4292e250f5f91012e6d620
5b4292e250f5f91012e6d6b1
5b4292e250f5f91012e6d688
5b4292e250f5f91012e6d6bf
5b4292e250f5f91012e6d6d9
5b4292e250f5f91012e6d7c5
5b4292e250f5f91012e6d5f8

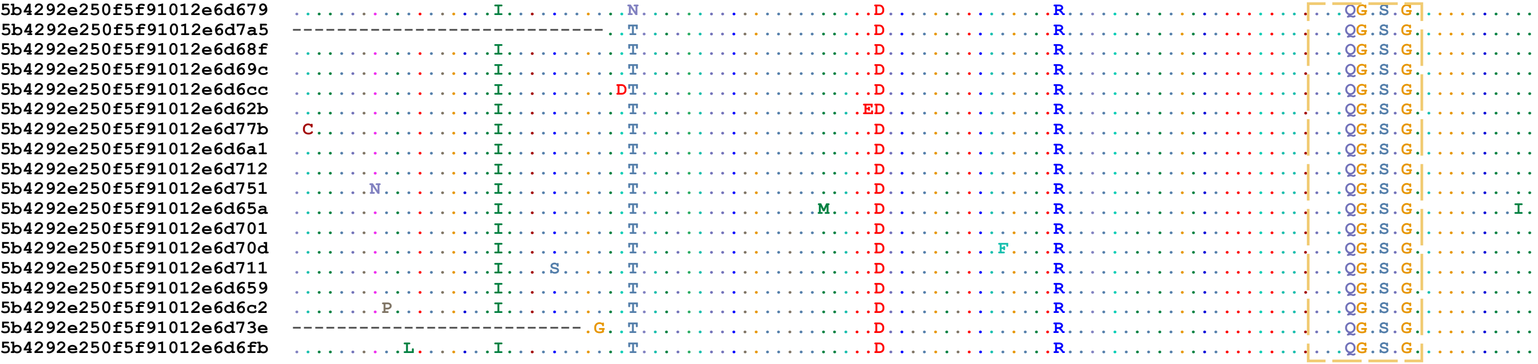
5b4292e250f5f91012e6d679
5b4292e250f5f91012e6d7a5
5b4292e250f5f91012e6d68f
5b4292e250f5f91012e6d69c
5b4292e250f5f91012e6d6cc
5b4292e250f5f91012e6d62b
5b4292e250f5f91012e6d77b
5b4292e250f5f91012e6d6a1
5b4292e250f5f91012e6d712
5b4292e250f5f91012e6d751
5b4292e250f5f91012e6d65a
5b4292e250f5f91012e6d701
5b4292e250f5f91012e6d70d
5b4292e250f5f91012e6d711
5b4292e250f5f91012e6d659
5b4292e250f5f91012e6d6c2
5b4292e250f5f91012e6d73e
5b4292e250f5f91012e6d6fb
